# Unraveling the Regulatory Mechanisms Underlying Tissue-Dependent Genetic Variation of Gene Expression

**DOI:** 10.1371/journal.pgen.1002431

**Published:** 2012-01-19

**Authors:** Jingyuan Fu, Marcel G. M. Wolfs, Patrick Deelen, Harm-Jan Westra, Rudolf S. N. Fehrmann, Gerard J. te Meerman, Wim A. Buurman, Sander S. M. Rensen, Harry J. M. Groen, Rinse K. Weersma, Leonard H. van den Berg, Jan Veldink, Roel A. Ophoff, Harold Snieder, David van Heel, Ritsert C. Jansen, Marten H. Hofker, Cisca Wijmenga, Lude Franke

**Affiliations:** 1 Department of Genetics, University Medical Center Groningen, University of Groningen, Groningen, The Netherlands; 2 Department of Epidemiology, University Medical Center Groningen, University of Groningen, Groningen, The Netherlands; 3 Department of Pathology and Medical Biology, Molecular Genetics, University Medical Center Groningen, University of Groningen, Groningen, The Netherlands; 4 Hanze University Groningen, Groningen, The Netherlands; 5 Department of Medical Oncology, University Medical Center Groningen, University of Groningen, Groningen, The Netherlands; 6 Department of Surgery, University Hospital Maastricht and Nutrition and Toxicology Research Institute (NUTRIM), Maastricht University, Maastricht, The Netherlands; 7 Department of Pulmonology, University Medical Centre Groningen, University of Groningen, Groningen, The Netherlands; 8 Department of Gastroenterology and Hepatology, University Medical Center Groningen, University of Groningen, Groningen, The Netherlands; 9 Department of Neurology, Rudolf Magnus Institute of Neuroscience, University Medical Center Utrecht, Utrecht, The Netherlands; 10 Department of Medical Genetics, University Medical Center Utrecht, Utrecht, The Netherlands; 11 Blizard Institute of Cell and Molecular Science, Barts and The London School of Medicine and Dentistry, Queen Mary University of London, London, United Kingdom; 12 Groningen Bioinformatics Centre, Groningen Biomolecular Sciences and Biotechnology Institute, University of Groningen, Haren, The Netherlands; Georgia Institute of Technology, United States of America

## Abstract

It is known that genetic variants can affect gene expression, but it is not yet completely clear through what mechanisms genetic variation mediate this expression. We therefore compared the *cis*-effect of single nucleotide polymorphisms (SNPs) on gene expression between blood samples from 1,240 human subjects and four primary non-blood tissues (liver, subcutaneous, and visceral adipose tissue and skeletal muscle) from 85 subjects. We characterized four different mechanisms for 2,072 probes that show tissue-dependent genetic regulation between blood and non-blood tissues: on average 33.2% only showed *cis*-regulation in non-blood tissues; 14.5% of the eQTL probes were regulated by different, independent SNPs depending on the tissue of investigation. 47.9% showed a different effect size although they were regulated by the same SNPs. Surprisingly, we observed that 4.4% were regulated by the same SNP but with opposite allelic direction. We show here that SNPs that are located in transcriptional regulatory elements are enriched for tissue-dependent regulation, including SNPs at 3′ and 5′ untranslated regions (*P* = 1.84×10^−5^ and 4.7×10^−4^, respectively) and SNPs that are synonymous-coding (*P* = 9.9×10^−4^). SNPs that are associated with complex traits more often exert a tissue-dependent effect on gene expression (*P* = 2.6×10^−10^). Our study yields new insights into the genetic basis of tissue-dependent expression and suggests that complex trait associated genetic variants have even more complex regulatory effects than previously anticipated.

## Author Summary

Gene expression can be affected by genetic variation, e.g. single nucleotide polymorphisms (SNPs). These are called expression-affecting SNPs or eSNPs. Gene expression levels are known to vary across different tissues in the same individual, despite the fact that genetic variation is the same in these tissues. We explored the different mechanisms by which genetic variants can mediate tissue-dependent gene expression. We observed that the genetic variants that associated with complex traits are more likely to affect gene expression in a tissue-dependent manner. Our results suggest that complex traits are even more complex than we had anticipated, and they underline the great importance of using expression data from tissues relevant to the disease being studied in order to further the understanding of the biology underlying the disease association.

## Introduction

It has become clear that human genetic variants, such as single nucleotide polymorphisms (SNPs), can in *cis* affect the expression of nearby genes [1], [2]. Many loci exist that contain genetic variants that affect gene expression (expression quantitative loci, eQTL, usually assessed by investigating single nucleotide polymorphisms (SNPs) and expression probes that are within 250 kb up to 1 Mb apart). These *cis*-eQTL analyses have been performed in many different human tissues and cell types, including lymphoblastoid cell lines (LCL) [3], [4], liver [5]–[7], blood [8], [9], brain [10], [11], adipose tissues [6], [8], skin [12], [13] and primary fibroblasts [12]. However, considerable heterogeneity of *cis*-eQTL effects is possible between different tissues: A recent study reported that the proportion of heritability due to gene expression attributable to *cis*-regulation differs between tissues (37% in blood and 24% in adipose tissue) [14]. By comparing the overlap of significant *cis*-eQTL at a predefined threshold, estimates on the tissue-dependence of *cis*-eQTL were between 30% (liver, adipose tissues) and 70–80% (LCLs, fibroblasts, T cells) [8], [9], [15], [16]. However, due to statistical power issues, it is likely that the tissue-dependency of *cis*-eQTL has been overestimated by studies solely assessing the overlap of *cis*-eQTL between tissues based on a certain threshold. Realizing this problem, Ding *et al.* used a refined statistical method to estimate the percentage of overlap by adding a power parameter to the model [12]. They reported that only 30% of *cis*-eQTL in LCLs were not shared with fibroblast *cis*-eQTL. Similarly, a recent study by Nica *et al.* [13] examined the tissue-dependence of *cis*-eQTL in three human tissues (LCL, skin and fat) in a continuous manner by quantifying the proportion of overlap of *cis*-eQTL from the enrichment of low *P*-values. They observed that 29% of *cis*-eQTL appear to be exclusively tissue-dependent, and also observed that the effect sizes of 10–20% of the *cis*-eQTL present in multiple tissues differ per tissue type. These observations are in line with a large-scale transcriptomic analysis of 46 human tissues, which found that while only 6.0% of genes were ubiquitously expressed across all the assessed tissues, 3.1% genes were only expressed in a single tissue [17].

To gain a better understanding of this subtle regulation of tissue-dependent regulation and to address the question of how genetic variants mediate tissue-dependent expression, we compared *cis*-regulation between whole peripheral blood from a large cohort of 1,240 individuals and four smaller primary human tissues (liver, subcutaneous adipose tissue (SAT), visceral adipose tissue (VAT) and skeletal muscle) obtained from a set of 85 subjects. We first applied a robust sampling procedure to estimate accurately how often genes showed different *cis*-eQTL effects between tissues. We then investigated in what way genes are differently associated with SNPs in different tissues. Finally, we assessed various functional properties for the SNPs involved in tissue-dependent *cis*-regulation and their association with complex traits.

## Results

### Cis-eQTL Mapping in Five Primary Tissues

For this study, we collected data for four different tissues from a set of 85 unrelated obese Dutch subjects. We successfully collected data on 74 liver samples, 62 muscle samples, 83 subcutaneous adipose tissue (SAT) samples and 77 visceral adipose tissue (VAT) samples (for 48 individuals all four tissues were available). The fifth tissue, blood, was collected from a different group of 1,240 unrelated Dutch individuals (Table S1). The gene expression levels in all five tissues were profiled using the same Illumina HumanHT12 v3 platform (see Materials and Methods). After normalization, we further removed strong expression differences between these tissues by removing the 50 principal components from this dataset and using the residuals for further analysis (described in [18] and Materials and Methods, Figure S1). We first performed *cis*-eQTL analysis in each of these datasets separately, by testing the correlation between SNPs and probes that were mapping within 1 Mb distance. At a false-discovery rate (FDR) of 0.05 level, we identified a non-overlapping set of 195,078 probe-SNP pairs that were significant in at least one of the tissues under study: 4,700 probe-SNP pairs were significantly associated in liver, 7,161 pairs significantly in SAT, 5,323 pairs significantly in VAT, 1,971 pairs significantly in muscle, and 190,278 pairs significant in blood (Figure S2). Owing to the much larger sample size, 182,569 probe-SNP pairs (93.6%) were solely detected in blood, while only 601 probe-SNP pairs (0.31%) were significant in each of the five different tissues (Figure S3). Although a previous study showed that the heritability of gene expression levels are higher in blood (37%) compared to adipose tissue (24%) [14], we believe that the large difference in the detected probe-SNP pairs between blood and non-blood tissues is due to statistical power issues that result from substantial sample size differences. As we had initially run *cis*-eQTL analyses in each of the tissues separately, we subsequently conducted a weighted Z-score meta-analysis across the four non-blood tissues and detected 23,878 probe-SNP pairs at FDR of 0.05. Out of these, 23.2% (5,550 out of 23,878 probe-SNP pairs) had not been identified in any of the single-tissue analyses (Figure S4). In total, the single-tissue analyses and meta-analysis yielded a non-overlapping set of 200,629 significant probe-SNP pairs, corresponding to 103,968 unique expression altering SNPs (eSNPs) and 11,618 probes (*eProbes*) that represent 8,561 unique genes (*eGenes*) (Figure S2).

### Cis-eQTL Effects Differ per Tissue Type

To assess the tissue-dependency of the *cis*-eQTL, we compared the Spearman correlation of each probe-SNP pair between tissues. However, due to the small sample sizes of the non-blood datasets we had very limited statistical power to determine whether there were *cis*-eQTL effect differences between non-blood tissues. We therefore confined ourselves to comparisons between the large blood dataset and each of the smaller non-blood tissues. To correct for sample size differences, we employed a resampling procedure, permitting us to derive an empirical distribution of association Z-scores (calculated based on the Spearman correlation) of each probe-SNP pair in blood of the same sample size as in non-blood tissues (see Materials and Methods; Figure S5). We observed that 18,456 pairs (9.2% of 200,629 probe-SNP pairs) showed a significantly different Z-score between blood and at least one of the non-blood tissues at *P*<6.23×10^−8^ (corresponding to a conservative Bonferroni-corrected *P*<0.05), implying a discordant association between blood and non-blood tissues. The remaining 182,173 probe-SNP pairs, which we called “concordant association”, had similar association Z-scores between the tissues under study (Figure S2). The “discordant associations” accounted for 15.4% of the *e*SNPs (15,974 out of 103,968 *e*SNPs), 28.7% of the *eProbes* (3,330 out of 11,618 *eProbes*), and 34.1% of the unique *eGenes* (2,919 out of 8,561 *eGenes*) (Table S2 and Figure S2). We further assessed for each probe-SNP pair, whether the discordance was detected between blood and multiple non-blood tissues, or only between blood and one specific non-blood tissue. We observed that 14,388 probe-SNP pairs (78.0% of the 18,456 discordant probe-SNP pairs) only showed a discordant effect between blood and one specific non-blood tissue. Only 125 probe-SNP pairs (corresponding to 31 *eProbes*) showed a discordant association in all four comparisons, suggesting similar regulation in the four non-blood tissues but markedly different regulation in blood (Figure S6). As such these results reveal there are considerable differences in the genetically determined regulation of gene expression between liver, SAT, VAT and muscle tissues, even though the RNA from these tissues had been derived from the same individuals at was collected at exactly the same time.

To ensure that our sampling procedure was robust, we used the same procedure to assess how often our method incorrectly concluded that a probe-SNP Spearman correlation differed between two independent eQTL datasets in the same peripheral blood tissue: We used the 1,240 blood samples as discovery set and used an independent set of 229 blood samples as validation whose expression was profiled using Illumina H8-v2 chips, [18], [19], see Methods and Materials. In this analysis, our method incorrectly deemed that 0.45% of the probe-SNP pairs showed a significant difference at the previously used P<6.23×10^−8^ level (Figure S7). In our comparisons between blood and non-blood tissues we had observed that 9.2% of the probe-SNP pairs showed a discordant effect, which is substantially higher and indicates that the number of discordant associations that we identified when comparing different tissues are not expected by chance (Fisher's exact test: OR = 20.6 and P<10^−300^). We also assessed whether imputation accuracy differences between datasets might confound some of the results, but did not find evidence this to be the case (see Materials and Methods).

### Properties of eSNPs

For the significant 200,629 probe-SNP pairs, we observed that for 146,480 pairs (73.0%) the eSNPs were located within 250 kb distance of the *eProbe* while 54,149 probe-SNP pairs (27.0%) mapped between 250 kb and 1 Mb apart. Consistent with a previous study [15], we observed that eSNPs at a larger distance from the probes tend to have smaller effects (Figure S8). However, we realize that due to extensive LD many different SNPs are usually significantly correlated with one single *cis*-eQTL probe. To address this, we performed step-wise conditional analyses in each tissue type to ascertain whether there were multiple SNPs that independently affected the expression levels of the same probe. We observed this for 26.8% of the *eProbes* in the large blood dataset (Table S3), (where for 2,794 out 10,443 *eProbes* we had detected multiple independent eSNPs): We observed that the secondary, tertiary and quaternary eSNPs usually map further away from the probe (Wilcoxon test P = 2.25×10^−66^, Figure S9), potentially reflecting some regulatory elements such as enhancers that usually reside further away from genes. In the non-blood tissues, we lacked statistical power to detect many secondary and tertiary effects (Table S3).

Interestingly, there was a very high overlap between the discordant *eProbes* (detected in our comparison across tissues) and the *eProbes* with multiple independent effects in blood (detected in the aforementioned analysis that solely used blood samples). Out of the 10,443 *eProbes* in blood, 2,528 *eProbes* had discordant association and 7,915 *eProbes* had concordant *association*. We observed that 47.5% of the discordant *eProbes* had multiple independent eSNPs present in blood (1,202 out of 2,528); whereas only 20.1% of the concordant *eProbes* had multiple independent eSNPs (1,592 out of 8,219, Fisher's exact test P = 3.85×10^−81^). This observation suggests that for *eProbes*: 1) different independent eSNPs can exist and 2) these independent eSNPs can exert an effect in one tissue while they do not exert an effect in another tissue.

We subsequently analyzed the most significant eSNP per *eProbe* per tissue and the top eSNP per *eProbe* from the meta-analysis of four non-blood tissues. In total, we ended up with 13,603 probe-SNP pairs (12,549 top eSNPs, that were affecting 11,575 probes pairs) these six analyses. Among them, 2,612 probe-SNP pairs (19.2%) showed a discordant effect among tissues at P = 6.23×10^−8^ level (genome-wide test level), accounting for 2,466 (19.7%) unique eSNPs.

**Figure 1:**
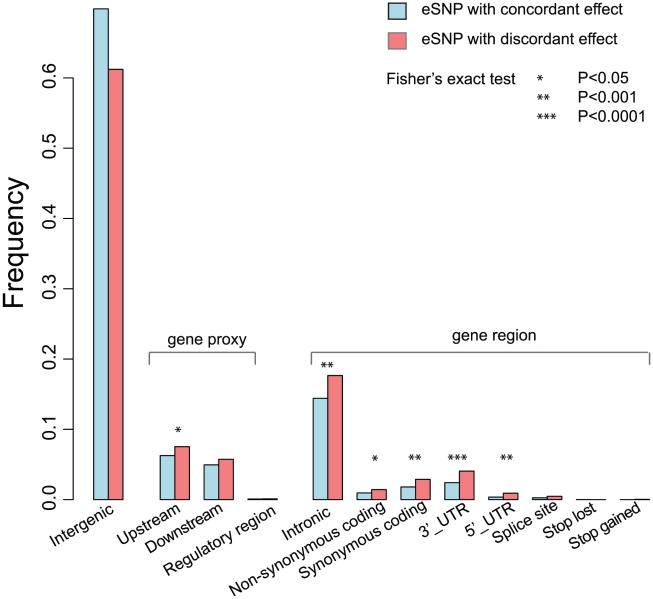
Functional Properties of eSNPs with tissue-dependent effect and concordant effect. The bar plot shows the frequency of the eSNP per function property. The eSNPs were annotated using the web-based tool of SNP Annotation and Proxy Search (SNAP; http://www.broadinstitute.org/mpg/snap/), based on the HapMap CEU population panel (release 22) and genome build 36.3. The asterisks indicate the significance of Fisher's exact test by comparing the eSNPs with concordant effect and with discordant effect, as given in the legend.

We found that the top eSNPs with discordant effect had a significantly higher minor allele frequency (MAF) than the concordant top eSNPs (Wilcoxon test P = 8.27×10^−21^). The eSNPs at a smaller distance from the *eProbe* (≤250 kb) were more likely to show a discordant effect compared to the eSNPs at larger distance (250 kb–1 Mb distance, OR = 1.62, P = 3.6×10^−22^, Figure S10). Although we acknowledge that the top eSNPs do not necessarily reflect the true causal variants, we annotated the functional properties of the top eSNPs to understand the potential roles of the eSNPs (irrespective of whether these reflect concordant or discordant *eProbes*). We observed that the most of the eSNPs were located in intragenic regions (67.0%) and intronic regions (14.9%), where their function often remains undetermined. Interestingly, eSNPs with discordant effect were (compared to concordant eSNPs) significantly enriched for synonymous-coding SNPs (Fisher's exact *P* value 9.9×10^−4^), and more often mapped in the 3′ and 5′ untranslated regions (UTRs, Fisher's exact *P* values 1.84×10^−5^ and 4.7×10^−4^, respectively) (Figure 1).

As shown before, we observed that SNPs, associated with complex traits and diseases, are more likely to be eSNPs [2], [6], [8], [18], [19]. We subsequently analysed 1,954 trait-associated SNPs (at *P*<5×10^−8^, retrieved from the GWAS catalog per 16 September 2011) [20] and observed that 907 trait-associated SNPs (46.4%) were eSNPs. Of these, 261 trait-associated eSNPs (28.7%) showed discordant effects on gene expression, which is significantly higher than what we observed for all 103,968 trait- and non-trait-associated eSNPs (15.4% discordant, Fisher's exact test *P* = 1.10×10^−33^) and also significantly higher than if we compare this to only the 12,549 top eSNPs (19.7% discordant, Fisher's exact test *P* = 2.6×10^−10^).

### Four Categories of Tissue-Dependent *Cis*-Regulation

As we have shown above, discordant *eProbes* are more likely to be influenced by multiple independent eSNPs. However, solely assessing the discordance of a single SNP-probe pair does not provide an extensive landscape of the tissue-dependent genetic determinants of gene expression. To gain further insight into this, we created ‘association profiles’ for the discordant *eProbes* and compared these across tissues. An association profile refers to the association Z-scores of all tested SNPs within 1 Mb distance of the *eProbe* under study (see Materials and Methods), and takes into account multiple SNPs and linkage disequilibrium. We created such association profiles for 2,007 discordant eProbes 52 (521 *eProbes* from liver, 708 *eProbes* from SAT, 526 *eProbes* from VAT, and 252 *eProbes* from muscle, Figure S2).

**Figure 2:**
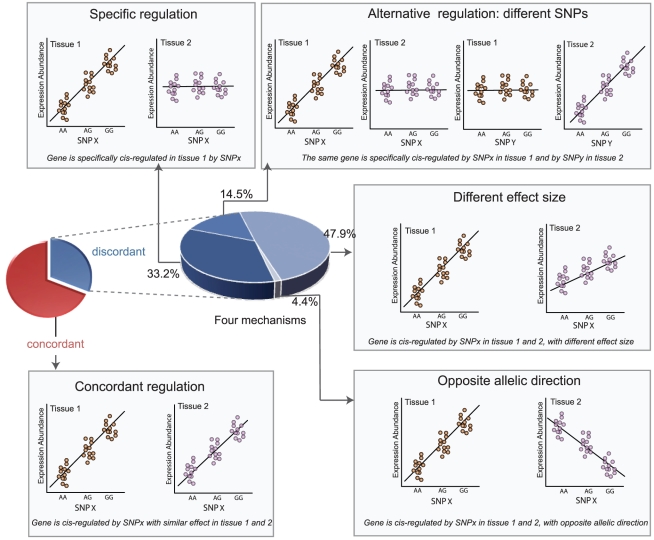
*cis*-regulation of gene expression between tissues. The associated probe-SNP pairs were classified to be concordant *or* discordant between tissues. The small pie plot shows the proportion of probes that have only concordant *association* (red part) or at least one discordant association (blue part). The probes with discordant association were under tissue-dependent regulation and we characterized four different mechanisms: specific regulation, alternative regulation, different effect size and opposite effect sizes. Their proportions are shown in the large blue pie plot. The concordant *cis*-regulation and the four different mechanisms are illustrated by the correlation between SNP genotypes (AA, AG and GG) and gene expression levels in two tissues: brown dots represent the expression of a gene in tissue 1 and purple dots the expression of a gene in tissue 2.

**Figure 3:**
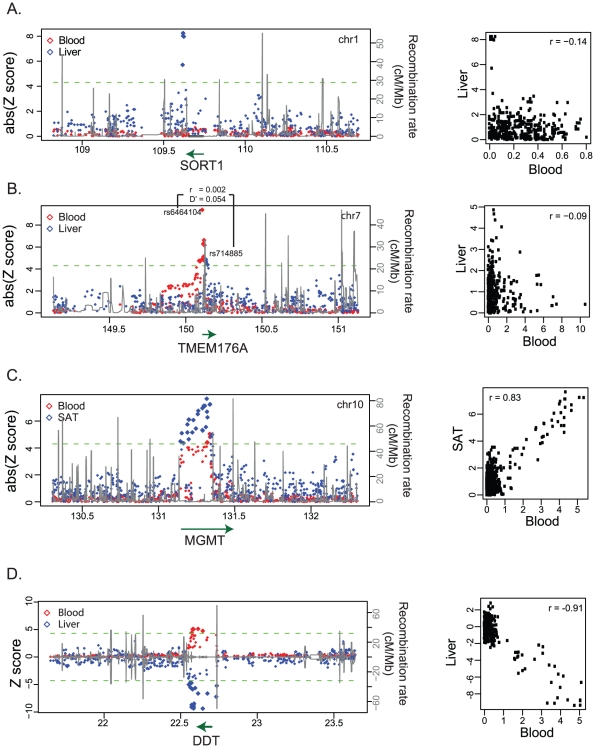
Case examples for tissue-dependent *cis*-regulation. (A) The liver-specific regulation of the SORT1 gene. (B) The alternative regulation of the TMEM176A gene in blood and liver. (C) The *cis*-regulation for the MGMT gene had different effect sizes in blood and SAT. (D) The *cis*-regulation for the DDT gene show opposite allelic direction between blood and liver. For each gene, the left panel shows the *cis*-eQTL association profile in the corresponding tissue (liver or SAT, in blue) vs the association profile in blood (red). The x-axis is the genome position based on genome build 36.3 (in Mb). The y-axis at the left is the association strength in terms of *Z-score*. The *Z-score* in blood has been weighted by the square root of the sample size, corresponding to the compared tissue. The dashed green line indicates the significance level of association at FDR 0.05. We use the absolute *Z-score*s to show the association in (A–C), but use the *Z-score*s in (D) for a better illustration of allelic direction. We assigned the association Z-scores in blood a negative value. If the allelic direction in SAT is the same as that in blood, the *Z-score* in SAT is negative too; otherwise, the *Z-score* in SAT is positive. The black line shows the recombination rate at this locus based on the HapMap II CEU panel and the scale is indicated on the right-hand y-axis. The green line with arrow at the bottom shows the genome position of the gene and the arrow indicates the transcription direction. The right panel shows the correlation of the *Z-score*s between two tissues. The r-value indicates the correlation coefficient of the Pearson correlation.

Upon inspection of these association profiles for the discordant *eProbes*, we identified four main different categories of tissue-dependent genetic regulation of gene expression. If the association profiles for one single *eProbe* did not correlate at all between two tissues, we further checked whether the *eProbe* was significant in both tissues: If the probe had a significant association in one tissue but not in the other, we deemed this “specific *cis*-regulation”. If instead the *eProbe* was significant in both tissues, but was associated to different (unlinked) eSNPs in the different tissues, we deemed it “alternative *cis*-regulation” between tissues. For those association profiles where two tissues showed a correlation, we checked the direction and the effect size of allelic effect on gene expression. If the allelic direction was the same and the effect size was different, we concluded the *eProbe* belonged to the category “different effect size”. If the allelic direction was instead opposite, the probes had tissue-dependent regulation with an “opposite allelic direction” (see Materials and Methods). We discuss each of these four categories in detail below and in Figure 2 and Figure 3.

#### Specific regulation

Specific *cis*-regulation refers to a gene that is *cis*-regulated in only one specific tissue. We found this type of regulation is a common phenomenon as it accounted for on average 33.2% of the discordant *eProbes* (Figure 2). One well-established example is the *SORT1* gene at the 1p13 cholesterol locus, to which SNPs map that affect low-density lipoprotein cholesterol (LDL-C) and the risk of myocardial infarction (MI) in humans [21], [22]. Recently, it was shown that the functional variant rs12740374 alters the binding site for C/EBP transcription factors and consequently alters the hepatic expression of the *SORT1* gene [23]. Our data replicated this specific *cis*-regulation in liver (Figure 3A). The association Z-score for rs12740374 with *SORT1* expression variation in liver was 8.24 (*N* = 74, *P* = 1.41×10^−15^) but in blood we observed no effect (*Z*-score = 0.07, *N* = 1,240, *P* = 0.8), nor did we observe any associations in SAT, VAT or muscle, and the association profiles for this gene show no correlation between different tissues (all spearman correlation *P* values>0.39). Thus, in our data, rs12740374 only exerts an effect on *SORT1* gene expression in liver, although we did observe that *SORT1* was expressed abundantly in all tissues.

#### Alternative regulation

Alternative regulation between tissues refers to a gene that is *cis*-associated with a SNP in a particular tissue and associated with a different, independent SNP in another tissue. Such an alternative *cis*-regulation is also a common phenomenon, as we found it applied to on average 14.5% of the probes with tissue-dependent regulation (Figure 2). One particular example is the trans-membrane gene *TMEM176A*, also known as hepatocellular carcinoma-associated antigen 112. The expression of *TMEM176A* was associated with intronic SNP rs714885 in liver (*N* = 74, *P* = 5.7×10^−6^) but with the 19.5 kb upstream SNP rs6464104 in blood (*N* = 1,240, *P* = 5.07×10^−132^) (Figure 3B). These two SNPs are unlinked variants (r^2^ = 0.002 and D′ = 0.054 based on the HapMap phase II CEU panel). We observed the same alternative association for different probes of *TMEM176A* in an independent liver eQTL dataset (profiled using a custom ink-jet microarrays [7] and in the aforementioned independent blood eQTL dataset that was profiled using Illumina HumanRef-8 v2 BeadChips) (Table S4) [18], [19]. This clearly shows that 1) multiple, unrelated variants can sometimes affect exactly the same gene, and 2) these independent variants sometimes only exert an effect on the gene expression in a particular tissue.

#### Different effect size

The different effect size refers to a common phenomenon that a gene is associated with the same SNP with alleles that have the same direction of effect but with a different magnitude in different tissues (Figure 2). For *eProbe* that showed this, we observed a significantly positive correlation between the association profiles of the tissues. We observed it applies to on average 47.9% of the probes that show tissue-dependent regulation (Figure S2), in line with a previous report [13]. One example is the O-6-methylguanin-DNA-methyltransferase (*MGMT*) gene that plays an important role in DNA repair and which suppresses tumor development [24]. We observed a *cis*-eQTL for *MGMT* across each of the five tissues. However, the effect size in blood was substantially smaller than that in SAT tissues (Figure 3C).

#### Opposite allelic direction

Surprisingly, we observed that some genes were associated with the same SNPs in different tissues but with alleles having an opposite effect on the gene expression between tissues. For a probe under this regulation, we then also observed a strong negative correlation between its association profiles across different tissues. This “opposite allelic direction” mechanism accounted for on average 4.4% of the probes under tissue-dependent regulation (Figure 2), which is much less common than the three previous mechanisms. However, this is still much more often than would be expected by chance, as determined by a comparison between two blood datasets in which we found the allelic directions were nearly always identical (Figure S7). One striking opposite allelic direction was observed to D-dopachrome tautomerase (*DDT*), which showed completely opposite effects between blood and liver (Figure 3D). Consistently, we found this opposite effect in the independent liver [7] and blood dataset,(H8v2), even when different probes were assessed. The minor allele rs5751777-C was associated with higher expression in liver (*P* = 9.95×10^−22^ in the discovery set and *P* = 2.86×10^−211^ in the validation set), but with lower expression in blood (*P* = 3.98×10^−119^ in the discovery set and *P* = 4.37×10^−24^ in the validation set) (Table S5). Strikingly, this opposite allelic direction was also observed when comparing liver with SAT, VAT and muscle, tissues that were all obtained from exactly the same set of individuals (Figure S11).

Another notable gene with an opposite allelic direction is *ORMDL3*. Although its function remains unclear, genetic variants near *ORMDL3* are associated with various immune-related diseases, including asthma, type 1 diabetes, Crohn's diseases, ulcerative colitis and primary biliary cirrhosis [25]–[29]. *ORMDL3* had a genome-wide significant *cis*-eQTL in blood and its association in SAT was showing near-genome-wide significance (Figure S12). All disease-associated SNPs in this locus showed association in *cis* with the expression level of *ORMDL3* (Table S6), including the functional variant rs12936231 that has been implicated to play a causal role in chromatin remodeling [30]. The risk alleles for asthma and preventive alleles for other autoimmune diseases showed consistent up-regulation in blood (and were also reported in LCLs) [25], [30]. However, to our surprise, the effect in SAT was completely reversed, leading to down-regulation.

Although we have only provided a few examples here, these observations indicate that conclusions drawn about mechanistic up- or down-regulation from a single tissue cannot necessarily be translated to other tissues, as they may sometimes lead to completely different conclusions depending on the tissues studied. In the supplementary material (Tables S7, S8, S9, S10 and Figures S13, S14, S15, S16), we have summarized the observed tissue-dependent regulation for 156 genes that have been reported to be associated with complex traits at P = 5×10^−8^ (based on the genes, mentioned in the Catalog of Published Genome-wide Association Studies, as of 16/09/2011). Some of these plots also show that the genetic regulation of gene expression is sometimes even more complicated than what we have described here: some genes can have multiple *cis*-eQTL that were either shared or specific to the tissues, e.g, the association of *MTMR3* gene that was associated with lung cancer [31], Nephrophaty [32], and inflammatory bowel disease [33], [34] (Figure S17).

**Figure 4:**
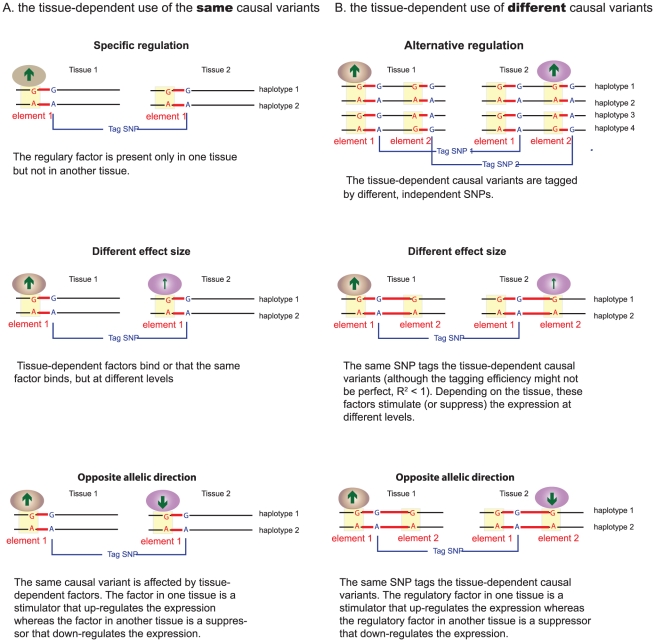
Molecular models of tissue-dependent *cis*-regulation. The observed tissue-dependent *cis*-regulations can be explained by two molecular models: (A) the tissue-dependent use of the same causal variants, or (B) the use of tissue-dependent causal variants. The ovals indicate the two regulatory factors (e.g., transcription factors) that play regulatory roles in different tissues (brown in tissue 1 and purple in tissue 2). These factors can recognize the same or different *cis*-elements (the yellow region). The genetic variants are shown as SNPs with A/G alleles. The SNPs in red are causal variants and the SNPs in blue are tag SNPs. The red line between them indicates the linkage disequilibrium. The arrows indicate the effect of regulatory factors, here the up arrows represent expression stimulators and the down arrows expression suppressors. The size of the arrows indicates the size of the differences between the expression of A and G alleles, i.e., the *cis*-eQTL effect size.

The four categories of tissue-dependent *cis*-regulation we have observed can be explained by two molecular models: 1) the tissue-dependent use of the same causal variant, i.e., the same eSNPs tag the same causal variant that is activated differentially by tissue-dependent factors; 2) the tissue-dependent causal variants, i.e., the same or different eSNPs tag different causal variants upon the tissues under study. The extent of the linkage disequilibrium (LD) between the causal variants and tag eSNPs, and the direction of effect of the regulatory factors (e.g., stimulating or suppressing the expression) and the size of their effects could lead to the observations of different categories (Figure 4).

## Discussion

Gene expression levels are partly determined by genetic variation, and eQTL mapping in different cell types and tissues has identified many *cis*-eQTL. However, the effect of *cis*-eQTL is strongly dependent upon the studied tissue. In this study, we compared the genetic architecture of gene expression regulation in blood and four non-blood primary tissues. We detected that the majority (71.3%) of the detected probes under genetic control (*eProbes*) show a concordant association across tissues. However, the remaining 28.7% of the *eProbes* show discordant, tissue-dependent regulation. Strikingly, many of those discordantly associated *eProbes* are affected by multiple, independent eSNPs. We followed up the genes under tissue-dependent regulation and identified four different mechanisms: specific regulation, alternative regulation, different effect size, and opposite allelic direction. We are the first to provide a comprehensive landscape of the different mechanisms of tissue-dependent *cis*-regulation. Of the four mechanisms identified, the opposite allelic direction mechanism, where alleles can have opposing effects on gene expression between tissues is of particular interest: Although this mechanism is less common than the other three, it has important implications for inferring the transcriptional effects of alleles from other tissue data, especially on the susceptibility risk alleles for complex diseases. The use of different tissues could result in completely the opposite conclusion! This finding highlights the great importance of investigating disease-relevant tissues in order to correctly characterize the functional effects of disease-associated variants.

We observed that SNPs at various transcriptional regulatory regions more often than expected exert tissue-dependent regulation, although most of the eSNPs were located at intergenic and gene intronic regions where functions remain undefined. However, we must emphasize that the causal variants remained undefined. Furthermore, because of the LD structure, although the same eSNPs can be associated with the expression of the same gene in different tissues, this does not necessarily mean that the same regulatory variants act in the different tissues. We have proposed two molecular models and suggested that tissue-dependent *cis*-regulations can be explained by the tissue-dependent use of the same causal variants or by the use of different tissue-dependent causal variants. Further fine-mapping and functional analyses are needed to identify the causal variants and to understand how they are used in different tissues due to the limited resolution of *cis*-eQTL mapping: It is known that the size of regulatory *cis*-elements generally is only a few base pairs (i.e., the binding sites of transcription factors or microRNAs), whereas the size of linkage disequilibrium blocks is generally in a range of 10–100 kb [35]. Furthermore, as the molecular models that we have proposed are quite simple, we cannot exclude other molecular mechanisms acting in these processes, e.g., the competition of different regulatory factors and binding sites in different tissues, or the role of tissue-specific methylation [36], [37] and chromatin remodeling [38], etc.

It is well known that trait-associated SNPs are more likely to have effects on gene expression but, to our surprise, we found that they are also more likely to exert tissue-dependent effects. This observation adds an extra layer of complexity to complex traits.

We acknowledge that our study has some limitations: We compared *cis*-regulation between peripheral blood and four rather small non-blood tissues. We lacked statistical power to compare the cis-regulations between two non-blood tissues well. Secondly, the identified discordant eQTLs are determined by the limited tissues that we studied Thirdly, although we corrected for substantial expression differences across samples by employing principal component analysis, it is still possible that some of the observed tissue-dependent *cis*-regulation can be due to the tissue heterogeneity (i.e. different proportions of cell types per tissue). Likewise it is also possible that some of the identified discordant *cis*-eQTL could be due to differences in the base-line expression between tissues. However, we observed this to be the case for both concordant and discordant *cis*-eQTL when investigating the original (non-PCA corrected) expression data (see Table S11).

Nevertheless our results indicate that natural genetic varation can affect gene expression levels in complex ways. Further analyses using different tissues and specific cell types and using larger sample sizes are required to gain a deeper understanding of the genetic variation of gene expression and to gain better insight into the full complexity of disease.

## Materials and Methods

### Genotyping and Expression Profiling on Liver, Muscle, and Adipose Fat Tissues from the Same Population

#### Subjects

From April 2006 to January 2009, 85 morbidly obese Dutch subjects (23 male and 62 female subjects) with a body mass index (BMI) between 35 and 70 were included in the study. They all underwent elective bariatric surgery at the Department of General Surgery, Maastricht University Medical Centre. Patients with acute or chronic inflammatory diseases (e.g., autoimmune diseases), degenerative diseases, reported alcohol consumption (>10 g/day), and/or using anti-inflammatory drugs were excluded. The average age of the subjects was 43.9 with a range of 17 and 67 years. This study was approved by the Medical Ethical Board of Maastricht University Medical Centre, in line with the guidelines of the 1975 Declaration of Helsinki. Informed consent in writing was obtained from each subject personally. The subject information was provided in Table S1.

#### Genotyping

Venous blood samples were obtained after 8 hours fasting on the morning of surgery. DNA was extracted from this blood using the Chemagic Magnetic Separation Module 1 (Chemagen) integrated with a Multiprobe II Pipeting robot (PerkinElmer). All samples were genotyped using Illumina HumanOmni1-Quad BeadChips that contain 1,140,419 SNPs. Genotyping was performed according to standard protocols from Illumina.

#### RNA profiling in four tissues

Wedge biopsies of liver, visceral adipose tissue (VAT, *omentum majus*), subcutaneous adipose tissue (SAT, abdominal), and muscle (*musculus rectus abdominis*) were taken during surgery. RNA was isolated using the Qiagen Lipid Tissue Mini Kit (Qiagen, Crawley, West Sussex, UK, 74804). Assessment of RNA quality and concentration was done with an Agilent Bioanalyzer (Agilent Technologies, Santa Clara, USA). Starting with 200 ng of RNA, the Ambion Illumina TotalPrep Amplification Kit was used for anti-sense RNA synthesis, amplification, and purification according to the protocol provided by the manufacturer (Ambion, Austin, USA). 750 ng of complementary RNA was hybridized to Illumina HumanHT12 BeadChips and scanned on the Illumina BeadArray Reader. Raw probe intensity data for these samples was extracted using Illumina's BeadStudio Gene expression module v3.2 (No background correction was applied, nor did we remove probes with low expression).

### Genotyping and Expression Profiling on Blood

#### Subjects

The genetical genomics samples for blood were collected from unrelated Dutch individuals in four studies: 324 healthy individuals were collected in the University Medical Centre Utrecht, 414 amyotrophic lateral sclerosis (ALS) patients were collected in the University Medical Centre Utrecht, 49 ulcerative colitis (UC) patients from a part of the inflammatory bowel disease (IBD) cohort of the University Medical Centre Groningen, and 453 patients with chronic obstructive pulmonary disease (COPD) were collected with the NELSON study. All samples were collected after informed consent and approved by local ethical review boards. Individual sample information is provided in Table S1.

#### Genotyping and imputation

DNA from all samples was hybridized to oligonucleotide arrays from Illumina. 324 healthy individuals and 414 ALS patients were genotyped using the Hap370 platform. The 453 COPD patients and 49 UC patients were genotyped on the 610-Quad platform. Because the subjects with liver, muscle, adipose fat tissues were genotyped using more intensive genotyping platform Illumina HumanOmni1-Quad BeadChips, we further used program IMPUTE v2 to impute the genotypes of SNPs that presented in Omni1-Quad chips but not directly genotyped on Hap370 and 610-Quad platform [39]. The reference panel for imputation was the CEU population from HapMap release 22. The directly genotyped SNPs were coded as 0, 1 or 2, while the imputed SNP dosage values were called at a 0.95 confidence level, ranging between 0 and 2. In this way, we obtained the genotype of the same set of 1,140,419 SNPs for all five tissues under study.

#### RNA profiling

Anti-sense RNA was synthesized, amplified and purified using the Ambion Illumina TotalPrep Amplification Kit (Ambion, USA) following the manufacturers' protocol. Complementary RNA was hybridized to Illumina HumanHT-12 arrays and scanned on the Illumina BeadArray Reader. Raw probe intensity data for these samples was extracted using Illumina's BeadStudio Gene expression module v3.2 (No background correction was applied, nor did we remove proves with low expression).

### Genotyping and Expression Profiling in an Independent Blood Dataset of 229 Samples

#### Subjects

To ascertain whether our method for identifying tissue-dependent cis-eQTL was robust, we compared the large peripheral blood with an independent blood eQTL dataset that comprised 229 samples. We have described this cohort in previous studies [9], [18]. In brief, this study comprised 111 English celiac disease patients, 59 Dutch amyotrophic lateral sclerosis patients and 59 Dutch health controls. The peripheral blood (2.5 ml) was collected with the PAXgene system (PreAnalytix GmbH, UK).

#### Genotyping and imputation

The samples were genotyped using the Illumina (Illumina, San Diega, USA) HumanHap300 platform. We further used IMPUTE v2 to impute the genotypes of all HapMap II SNPs. The reference panel for imputation was the CEU population from HapMap release 22. The directly genotyped SNPs were coded as 0, 1 or 2, while the imputed SNP dosage values were called at a 0.95 confidence level, ranging between 0 and 2.

#### RNA profiling

Anti-sense RNA was synthesized amplified and purified using the Ambion Illumina TltalPrep Amplification Kit (Ambion, USA) following the manufacturers' protocol. Complementary RNA was hybridized to Illumina HumanRef-8 v2 arrays (further referred to as H8v2) and scanned on the Illumina BeadArray Reader.

### Normalization and PCA Correction

The raw expression intensities from five tissues were jointly quantile normalized and log_2_ transformed. We further applied a principal component analysis (PCA) on expression correlation matrix and observed that genes are differentially expressed among different tissue types (Figure S1). We argue that the dominant principal components (PCs) will primarily capture sample differences in expression that reflect physiological or environmental variation (e.g., tissue type and phenotype difference) as well as systematic experimental variation (e.g. batch and technical effect). In order to target the difference in the genetic variation of expression among tissues, we removed the global variation in expression among tissues by using the residual expression for each probe in each tissue after removing 50 PCs (identical to what we have described before [18]). Our previous analysis on the same dataset showed that the number of significantly detected *cis*-eQTL probes increased two-fold when 50 PCs were removed from the expression data (see Figure S7 in ref [18]). For the independent blood dataset with 229 subjects, we followed the same quantile normalized and PCA correction.

### Population Stratification and SNP Quality Control

We tested population stratification between the two cohorts using the program PLINK (http://pngu.mgh.harvard.edu/~purcell/plink/strat.shtml). This program uses complete linkage agglomerative clustering, based on pair-wise identity-by-state (IBS) distances. The fact that all the individuals from both cohorts were clustered together indicates there was no population stratification. We also checked the allelic frequencies between the two cohorts by treating the 85 individuals with four tissue samples as cases and the 1,240 individuals for blood samples as controls. For the imputed SNPs, we used the genotype with highest probability as the discrete genotype for QC purposes. We removed SNPs that showed significant differences in allele frequency at *P*<0.01. Then the SNPs were quality controlled for minor allelic frequency >5%, a call rate >95% and an exact Hardy-Weinberg (HWE) *P* value>0.001. To make certain on the directions of the allelic effect on gene expression (up-regulating or down-regulating), we further removed SNPs with two types of transversion alleles (A/T and G/C) and confined our analysis to SNPs with transition alleles (A/G or C/T) and other types of transversion alleles (A/C or G/T). This quality control resulted in 710,035 SNPs for further analysis.

### eQTL Discovery

In order to detect *cis*-eQTLs, analysis was confined to those probe-SNP combinations for which the distance from the probe transcript midpoint to SNP genomic location was ≤1 Mb. For each probe-SNP pair, we used Spearman correlation to detect association between SNPs and the variations of the gene expression in liver, SAT, VAT, muscle and blood, respectively. We calculated the Spearman correlation coefficient and corresponding *P* values and subsequently transformed this into a Z-score. To maximize the power of eQTL discovery in non-blood tissues, we further performed meta-analysis for four non-blood tissues that combines the association signals across the four non-blood tissues under study. An overall, joint *P* value was calculated using a weighted (square root of the dataset sample number) *Z*-method. Please see the ref [40] for a comprehensive overview of this method.

To correct for multiple testing, we controlled the false-discovery rate (FDR) at 0.05: the distribution of observed p-values was used to calculate the FDR, by comparison with the distribution obtained from permuting expression phenotypes relative to genotypes 100 times. At FDR = 0.05 level, the significance *P* value threshold was 1.37×10^−5^ for significantly associated probe-SNP pairs in liver, 2.07×10^−5^ for significant association in SAT, 1.54×10^−5^ for significant association in VAT, 5.64×10^−6^ for significant association in muscle, 4.8×10^−4^ for significant association in blood and 1.10×10^−4^ for significant association in the meta-analysis of four non-blood tissues. For these significant probe-SNP pairs, we termed the corresponding SNP, probe and genes as expression SNP (eSNP), regulated probe (*eProbe*) and regulated genes (*eGenes*), respectively.

### Conditional Regression Analysis to Detect Independent eSNPs

Due to the linkage disequilibrium among the tested SNPs, we usually found numerous eSNPs for each *eProbe*. In order to detect independent eSNPs, we performed conditional regression analysis for the *eProbes* per tissue type. For each *eProbe*, we first regressed out the main effect of the top eSNP. We then subjected the residuals to eQTL mapping to detect potential secondary, independent eSNPs. We again controlled the false discovery at 0.05 by running 100, as described before in the method section “eQTL discovery”. If secondary eSNPs were present, we repeated the entire procedure to detect tertiary eSNPs by regressing out both the primary and secondary effect (using appropriate multivariate regression analysis). This procedure was repeated until no significant associations were detected any more.

### Sampling Approach to Identify Tissue-Dependent eQTL

#### Comparing blood and non-blood tissues

For each of the 200,629 probe-SNP pairs that was significantly associated at FDR 0.05 level, we further assessed whether the detected Z-scores differed per tissue. We used the Z-scores in blood as a reference because the blood samples were independent from other tissue samples and the sample size was much larger. To correct for the sample size difference, we, out of the 1,240 blood samples, randomly selected without replacement the same number of samples for the comparison with liver (*N* = 74), SAT (*N* = 83), VAT (*N* = 77) and muscle (*N* = 62). For a certain probe-SNP pair, we re-calculated the association *Z-score* in blood for the selected sample size. The sampling procedure was repeated 100 times. We subsequently fitted a generalized extreme value distribution (GEVD) for the Z-scores of 100× sampling procedures in blood. GEVD is a flexible model with three parameters: location (γ), scale (β) and shape (α). GEVD can resemble different distributions with different settings of parameters. For example, when α = 0, it resembles the Gumbel types of distributions (Type I); when α>0, it resembles the Frechet types of distributions (Type II); when α<0, it resembles the Weibull types of distributions (Type III). Therefore, fitting the GEVD can permit us to estimate realistic distribution of the Z-scores of this certain probe-SNP pair in blood (Figure S3). We then assessed the deviation of the Z-score of the same probe-SNP pair in the other four tissues from the estimated GEVD in blood and computed *P* value for the difference of Z-scores between tissues. We did this analysis in R (version 2.10.1) using the package evd: Functions for extreme value distributions (version 2.2–4). This analysis was done for each of the 200,629 probe-SNP pairs and between blood and each of four non-blood tissues. Considering the possible dependence of the eQTL effect among tissues, the significance was controlled at the conserved Bonferroni-corrected 0.05, corresponding to a *P* value of 6.23×10^−8^ (0.05/200,629 probe-SNP pairs/4 tissue comparisons). The probe-SNP pairs with a *P*≤6.23×10^−8^ were called “discordant associations”, while probe-SNP pairs with *P*>6.23×10^−8^ were called “concordant associations”. The expression profiling in all five tissues used the same platform. Therefore, the discordant association cannot be explained by the hybridization efficiency. Because all of the tested SNPs were directly genotyped in non-blood tissues but most of them were imputed in blood, we further checked whether the discordance was caused by the imputation. We did not observe that imputation accuracy might confound our results: 69.3% of the discordant eSNPs were imputed in blood whereas 68.0% of the concordant eSNPs were imputed in blood (Fisher's exact test *P* value = 0.60). We also assessed whether there was heterogeneity in effect present when comparing the different subgroups of phenotypes. We did not find evidence this to be the case (see Table S6 in ref [18]).

#### Comparing two independent blood datasets

To further validate the tissue-dependent effect we had detected, we compared the *cis*-eQTL effects between the blood dataset HT12 and H8v2, using the same sampling procedure as described above. Because of the difference of expression platform, we could only make comparisons for those probes that were present in both datasets. We only investigated SNPs that showed similar allele frequencies between the two blood datasets (SNPs with allele frequency *P*<0.01 were excluded from analysis and as the H8v2 dataset contained 111 celiac disease patients that were nearly all HLA-DQ2.2 or HLA-DQ2.5 positive we also excluded the HLA from this analysis). After filtering we could compare 93,656 probe-SNP pairs.

### Enrichment for SNP Properties

The minor allele frequency (MAF) and function properties of eSNPs were annotated by the web-based tool SNP Annotation and Proxy Search (SNAP) (www.broadinstitute.rog/mpg/snap) [41], using the CEU population panel from HapMap release 22. We performed Fisher's exact test to compare the enrichment between eSNPs with a tissue-dependent effect on expression across tissues and eSNPs with a static effect.

### *Cis*-eQTL Analysis of Trait-Associated SNPs

To directly assess the effect of trait-associated SNPs on gene expression, we confined our *cis*-eQTL analysis to 1,954 SNPs (with alleles A/G) that were associated with complex traits at *P*<5.0×10^−8^ in the ‘Catalog of Published Genome-wide Associated Studies’ (per 16 September 2011) [20] and assessed the tissue-dependency of eQTL effect across the tissues, following the same analysis and permutation procedures. The *cis*-eQTL significance threshold *P* values were set at *P* = 4.6×10^−3^ in blood, 2.6×10^−4^ in liver, 2.5×10^−4^ in muscle, 1.8×10^−4^ in VAT and 3.2×10^−5^ in SAT, and 1.1×10^−3^ for the meta-analysis of four non-blood tissue. At these levels, a total of 2,990 probe-SNP pairs were significant in at least one eQTL analysis.

### Characterizing the Tissue-Dependent Mechanisms of *Cis*-Regulation

To characterize the tissue-dependent mechanisms of *cis*-regulation, we reasoned that comparing the association at a single probe-SNP level cannot provide a complete picture of the tissue-dependent genetic determinants of gene expression. To gain further insight into the tissue-dependent *cis*-regulation, we extended analysis for the *eProbes* with discordant *cis*-eQTL that were determined by single probe-SNP comparison and compared their whole association profiles across tissues. The association profile refers to the set of the absolute *Z*-scores of all N number of the tested SNPs within 1 Mb distance from the middle point of probe under study: i.e., {|*Z_1_*|, |*Z_2_|*, |*Z_3_*|, … |*Z_n_*|}. Such a profile can represent the combined association signals of the multiple independent eSNPs and their linkage disequilibrium. Most of the *eProbes* only showed significant association in blood and were not significantly associated in the smaller non-blood tissues. For those *eProbes*, we had limited statistical power to determine whether the association in non-blood tissues is truly absent or is not detected due to power issues. Therefore, we confined our comparison of association profiles to the *eProbes* that were significantly associated in non-blood tissues and compared them to those in blood. To assess the similarity of association profiles across tissues, we computed Pearson correlations coefficient (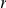) of the association profiles between two tissues. Because the SNPs were likely in strong linkage equilibrium, there is strong dependency among the *Z*-scores within the association profile. To determine the empirical threshold for the significance of the correlation between the association profiles and considering the dependency of the SNPs, we performed permutation analysis by randomly assigning genomes to the individuals per tissue type. We thus obtained the association profiles per probe per tissue for the permuted genotypes. These permuted association profiles retained the same correlation structure among SNPs and the Pearson correlation coefficient between the permuted association profiles (*r_0_*) would mainly explain the correlation among SNPs. We repeated this permutation 100 times and determined the empirical threshold *r_thres_* = 0.21 at FDR 0.05 level using the model (FDR = *n_0_*{*r_0_*≥*r_thres_*}/*n_1_*{*r*≥*r_thres_*), where *r* and *r_0_* refer to the Pearson correlation coefficient of real data and permuted data, respectively; *n* refers to the number of probes where *r≥r_thres_* and *n_0_* refers to the average number of probes where *r_0_≥r_thres_* from 100× permutations.

Based on the correlation of association profiles between tissues, we identified four different categories of tissue-dependent genetic regulation of gene expression. If the association profiles for one single probe did not correlate at all between two tissues (*r*<0.21), we further checked whether the *eProbe* was significant in both tissues: if the probe had a significant association in one tissue but not in the other, we deemed this “specific *cis*-regulation”; if instead the *eProbe* was significant in both tissues, but was associated to different (unlinked) eSNPs in the different tissues, we deemed it “alternative *cis*-regulation”. For those association profiles where two tissues showed a correlation (*r*≥0.21), we checked the direction and the effect size of allelic effect on gene expression: if the allelic direction was the same and the effect size was different, we concluded the *eProbe* belonged to the category “different effect size”; if the allelic direction was instead opposite, the probes had tissue-dependent regulation with an “opposite allelic direction”.

### Differential Expression

For the probes with tissue-dependent *cis*-regulation, we assessed whether they were also differential expressed between the tissues where they showed different *cis*-regulation. To do so, we relied upon the quantile-normalized expression intensity before any removal of the first 50 principal components. For each discordant *eProbe*, we used a Wilcoxon Mann-Whitney U test to assess the differential expression between the tissues. We performed the same analysis for a random set of concordant *eProbes, equal in size to the set of* discordant *eProbes*. The significance of differential expression was controlled at a Bonferroni-corrected *P* value 0.05 level.

### Accession Numbers

Expression data for both blood tissue and four non-blood dataset have been deposited in GEO with accession numbers GSE20142 (1,240 peripheral blood samples, hybridized to HT12 arrays) and GSE22070 (subcutaneous adipose, visceral adipose, muscle and liver samples). The expression data of the validation blood eQTL dataset (229 samples) has been deposited in GEO with accession number GSE203332.

## Supporting Information

Figure S1 The effect of removing principal components from expression data.
(PDF)

Figure S2 Flowchart for the analysis of the tissue-dependent *cis*-eQTL across the five human tissues.
(PDF)

Figure S3 Overlap of the associated probe-SNP pairs across the tissues.
(PDF)

Figure S4 Overlap of the associated probe-SNP pairs across the single-tissue analysis and meta-analysis.
(PDF)

Figure S5 Sampling procedure. We assessed the difference of association strength between blood and four other tissues (liver, SAT, VAT and muscle). As an example, for liver, we randomly sampled 74 subjects out of the 1,240 blood subjects (making the same sample size as for the liver tissue dataset) and re-measured the association strength for each significantly associated probe-SNP pair, in terms of *Z-scores*. This sampling procedure was repeated 100 times. The histogram showed the *Z-scores* distribution of a certain *cis*-eQTL in 74 blood subjects. We then assessed the deviation of the *Z-scores* detected in liver (the red arrow) from the distribution of *Z-scoress* in blood, by fitting the extreme value distribution (EVD) (the red line). The same analysis was performed for comparing blood with SAT, VAT and muscle, by randomly sampling *N* number of blood subjects (*N* = 83 for the SAT sample size; 77 for the VAT sample size, and 62 for the muscle sample size, respectively).
(PDF)

Figure S6 The overlap of discordantly associated probe-SNP pairs.
(PDF)

Figure S7 The comparison of Z-scores between two independent blood datasets. The comparison of *cis*-eQTL effect was confined to the set of 93,656 probe-SNP pairs that have been tested in two independent blood datasets, e.g., a discovery set of 1,240 subjects profiled on the Illumina HT12 expression platform (HT12) and a validation set of 229 subjects profiled on the Illumina H8v2 expression platform (H8v2). The *Z-scores* of *cis*-eQTL in the discovery set were the mean of *Z-scores* from 100× taking a sample of 229 out of the 1,240 blood subjects. The gray dots indicate the concordantly associated probe-SNP pairs between the two blood samples. The red dots indicate the discordantly associated probe-SNP pairs (the false-positive tissue-dependent association). The black line is the diagonal line.
(PDF)

Figure S8 The probes-SNP distance for associated probe-SNP pairs. The distance was calculated by the base pair position (bp) of SNPs minus the bp position of the middle point of the probes.
(PNG)

Figure S9 Probe-SNP distance for 2,794 *eProbes* in blood with multiple independent eSNPs.
(PDF)

Figure S10 The discordant probe-SNP pairs vs. the probe-SNP distance. The histogram shows the number the probe-SNP pairs with different distance. The numbers on each bar show the total number of probe-SNP pairs and the percentage of pairs with discordant association. The 2×2 table for Fisher's exact test is shown.
(PDF)

Figure S11 The direction of allelic effect of rs5751777 on *DDT* expression. The correlation between the genotype of rs5751777 and the expression intensity of *DDT* gene (residual variance after 50 PCs removed) in five tissues. Each dot represents one subject, red for females and blue for males. The X-axis represents the genotypes and the Y-axis represents the expression rank of the probes.
(PDF)

Figure S12 The opposite association of *ORMDL3* gene between blood and SAT. The x-axis is the genome position based on genome build 36.3 (in Mb). The y-axis at the left is the association profiles in terms of *Z-scores*. The *Z-scores* in blood, represented as the red dots, has been weighted by the square root of the sample size, corresponding to the compared tissue. The blue dots represent the *Z*-scores in SAT. The dashed green line indicates the significance level at FDR 0.05. For a better illustration of allelic direction, we assigned the association *Z*-scores in blood a positive value. If the allelic direction in SAT is the same as that in blood, the *Z-scores* in SAT are positive too; otherwise, the *Z-scores* in SAT are negative.
(PDF)

Figure S13 The association profiles of the selected trait-associated genes that show discordant association between blood and liver. The x-axis is the genome position based on genome build 36.3. The y-axis at the left is the association profiles in terms of the *Z*-score. The *Z*-score in blood, represented as the red dots or orange dots. The red dots refer to the Z-scores that have been weighted by the square root of the sample sizes, corresponding to the compared tissue. For the clarity of subtle effect in blood, the weak association in blood was shown as orange dots if the Z-scores have not been weighted by the sample size, i.e., the Z-scores reported in 1,240 subjects. The blue dots represent the Z-scores in liver. The dashed green line indicates the Z-score 3.49, representing the significance level in blood at FDR 0.05. The right panel shows the correlation of the absolute association *Z*-scores between two tissues. The rho-value indicates the correlation coefficient of the Pearson correlation.
(PDF)

Figure S14 The association profiles of the selected trait-associated genes that show discordant association between blood and SAT. The x-axis is the genome position based on genome build 36.3. The y-axis at the left is the association profiles in terms of the *Z*-score. The *Z*-score in blood, represented as the red dots or orange dots. The red dots refer to the Z-scores that have been weighted by the square root of the sample sizes, corresponding to the compared tissue. For the clarity of subtle effect in blood, the weak association in blood was shown as orange dots if the Z-scores have not been weighted by the sample size, i.e., the Z-scores reported in 1,240 subjects. The blue dots represent the Z-scores in SAT. The dashed green line indicates the Z-score 3.49, representing the significance level in blood at FDR 0.05. The right panel shows the correlation of the absolute association *Z*-scores between two tissues. The rho-value indicates the correlation coefficient of the Pearson correlation.
(PDF)

Figure S15 The association profiles of the selected trait-associated genes that show discordant association between blood and VAT. The x-axis is the genome position based on genome build 36.3. The y-axis at the left is the association profiles in terms of the *Z*-score. The *Z*-score in blood, represented as the red dots or orange dots. The red dots refer to the Z-scores that have been weighted by the square root of the sample sizes, corresponding to the compared tissue. For the clarity of subtle effect in blood, the weak association in blood was shown as orange dots if the Z-scores have not been weighted by the sample size, i.e., the Z-scores reported in 1,240 subjects. The blue dots represent the Z-scores in VAT. The dashed green line indicates the Z-score 3.49, representing the significance level in blood at FDR 0.05. The right panel shows the correlation of the absolute association *Z*-scores between two tissues. The rho-value indicates the correlation coefficient of the Pearson correlation.
(PDF)

Figure S16 The association profiles of the selected trait-associated genes that show discordant association between blood and muscle. The x-axis is the genome position based on genome build 36.3. The y-axis at the left is the association profiles in terms of the *Z*-score. The *Z*-score in blood, represented as the red dots or orange dots. The red dots refer to the Z-scores that have been weighted by the square root of the sample sizes, corresponding to the compared tissue. For the clarity of subtle effect in blood, the weak association in blood was shown as orange dots if the Z-scores have not been weighted by the sample size, i.e., the Z-scores reported in 1,240 subjects. The blue dots represent the Z-scores in muscle. The dashed green line indicates the Z-score 3.49, representing the significance level in blood at FDR 0.05. The right panel shows the correlation of the absolute association *Z*-scores between two tissues. The rho-value indicates the correlation coefficient of the Pearson correlation.
(PDF)

Figure S17 Association profiles of MTMR3 in blood and liver. The x-axis is the genome position based on genome build 36.3 (in Mb). The y-axis at the left indicates the association *Z*-score. The *Z*-scores in blood, represented as the red dots, have been weighted by the square root of the sample size, corresponding to the compared tissue. The blue dots represent the Z-scores in SAT. The dashed green line indicates the Z-scores 3.49, representing the significance level in blood at FDR 0.05. The right panel shows the correlation of the absolute association *Z-scores* between two tissues. The *r*-value indicates the correlation coefficient of the Pearson correlation.
(PDF)

Table S1 Characteristics of Samples.
(XLS)

Table S2 The number of discordant *cis*-eQTL between blood and non-blood tissues.
(DOC)

Table S3 The Number of independent eSNPs per probe.
(DOC)

Table S4 Replication of tissue-alternative cis-eQTL of TMEM176A.
(DOC)

Table S5 Replication of cis-eQTL of *DDT* in blood and liver that show opposite allelic direction.
(DOC)

Table S6 Allelic effect of disease-associated SNPs on the expression of *ORMLD3*.
(DOC)

Table S7 The tissue-dependent regulation of 45 trait-associated genes that show discordant association between blood and liver.
(XLS)

Table S8 The tissue-dependent regulation of 50 trait-associated genes that show discordant association between blood and SAT.
(XLS)

Table S9 The tissue-dependent regulation of 46 trait-associated genes that show discordant association between blood and VAT.
(XLS)

Table S10 The tissue-dependent regulation of 19 trait-associated genes that show discordant association between blood and Muscle.
(XLS)

Table S11 The number of the differentially expressed eProbes.
(DOC)
