# Supplementary material for: Unraveling the Regulatory Mechanisms Underlying Tissue-Dependent Genetic Variation of Gene Expression
Source: PLoS Genet. 2012 Jan 19;8(1):e1002431. doi: 10.1371/journal.pgen.1002431 (PMC3261927; doi:10.1371/journal.pgen.1002431)
Supplement: Figure S1 — The effect of removing principal components from expression data. (PDF) [file pgen.1002431.s001.pdf]

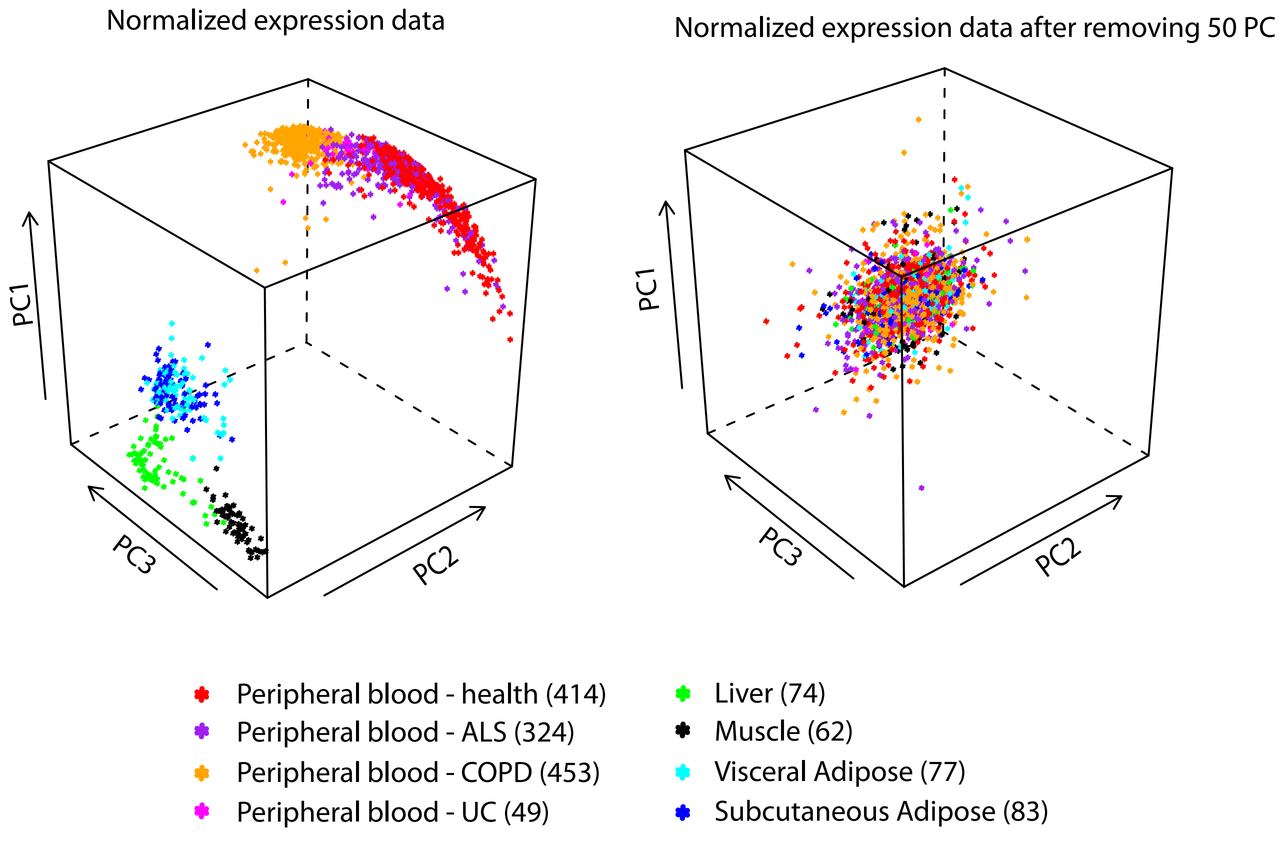
Normalized expression data
Normalized expression data after removing 50 PC
PC1
PC2
PC3
PC1
PC2
PC3
Peripheral blood - health (414)
Peripheral blood - ALS (324)
Peripheral blood - COPD (453)
Peripheral blood - UC (49)
Liver (74)
Muscle (62)
Visceral Adipose (77)
Subcutaneous Adipose (83)
