# Supplementary figures and images for: Unraveling the Regulatory Mechanisms Underlying Tissue-Dependent Genetic Variation of Gene Expression
Source: PLoS Genet. 2012 Jan 19;8(1):e1002431. doi: 10.1371/journal.pgen.1002431 (PMC3261927; doi:10.1371/journal.pgen.1002431)

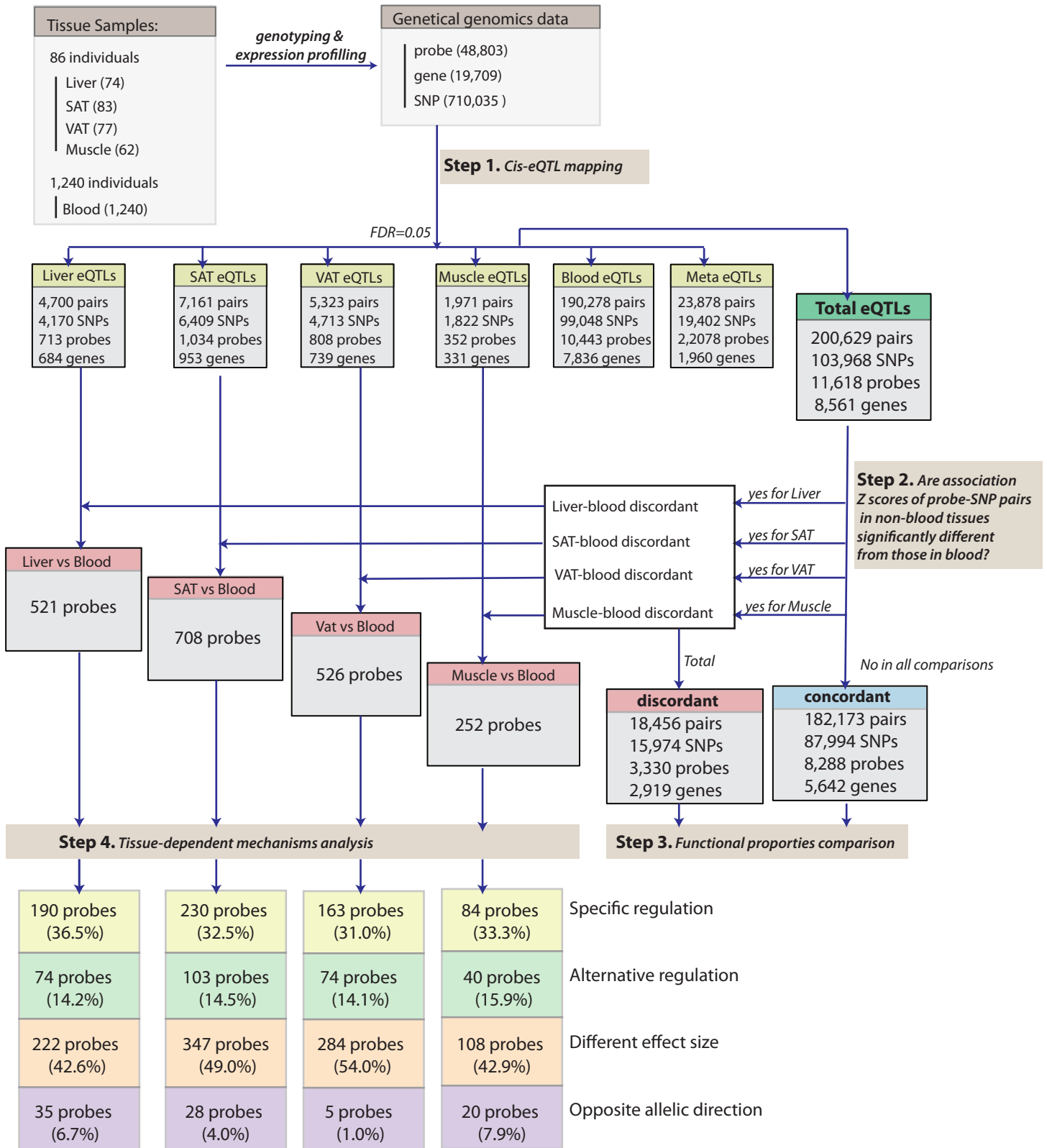

Supplement: Figure S2 — Flowchart for the analysis of the tissue-dependent cis-eQTL across the five human tissues. (PDF) [file pgen.1002431.s002.pdf]

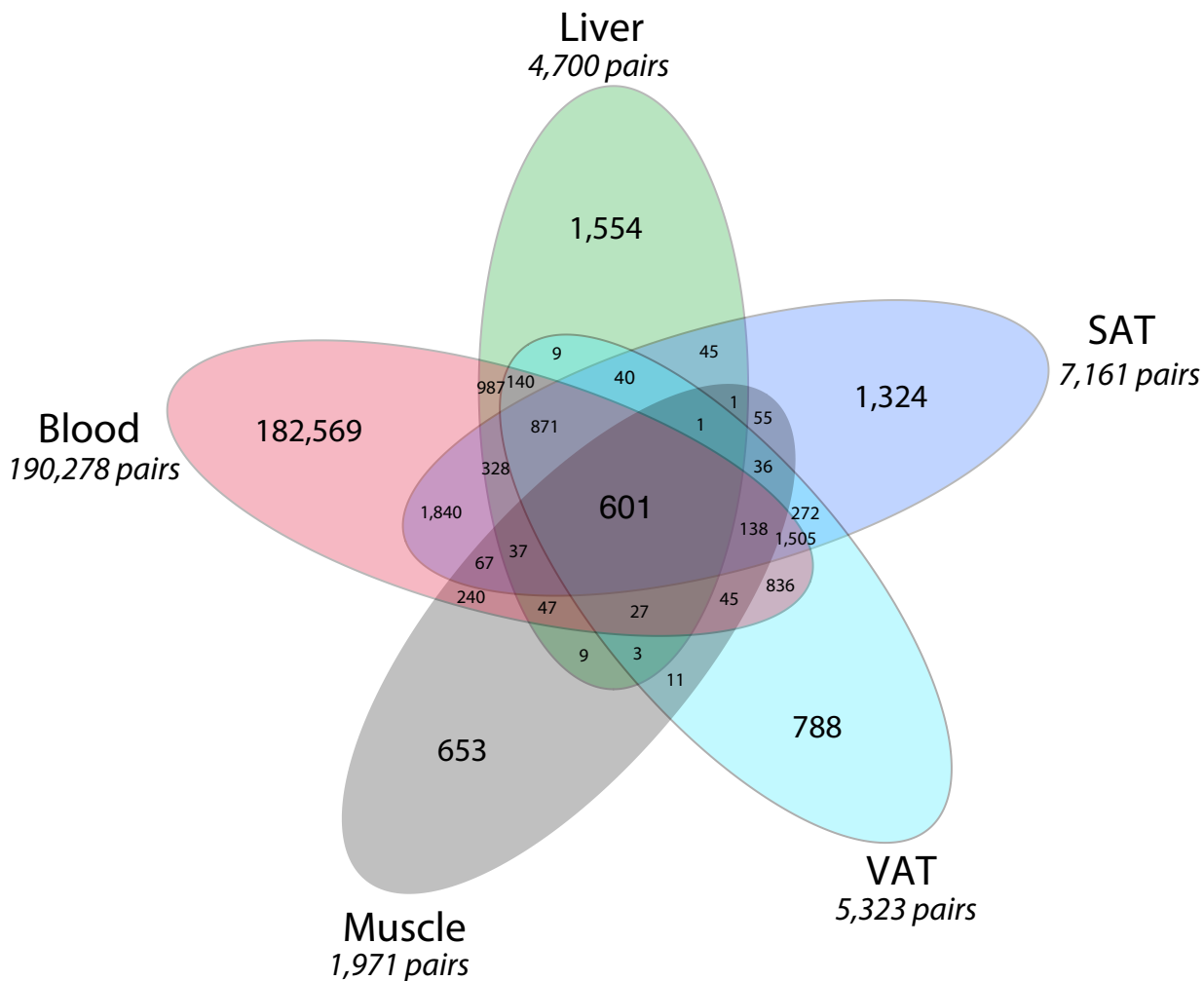

Supplement: Figure S3 — Overlap of the associated probe-SNP pairs across the tissues. (PDF) [file pgen.1002431.s003.pdf]

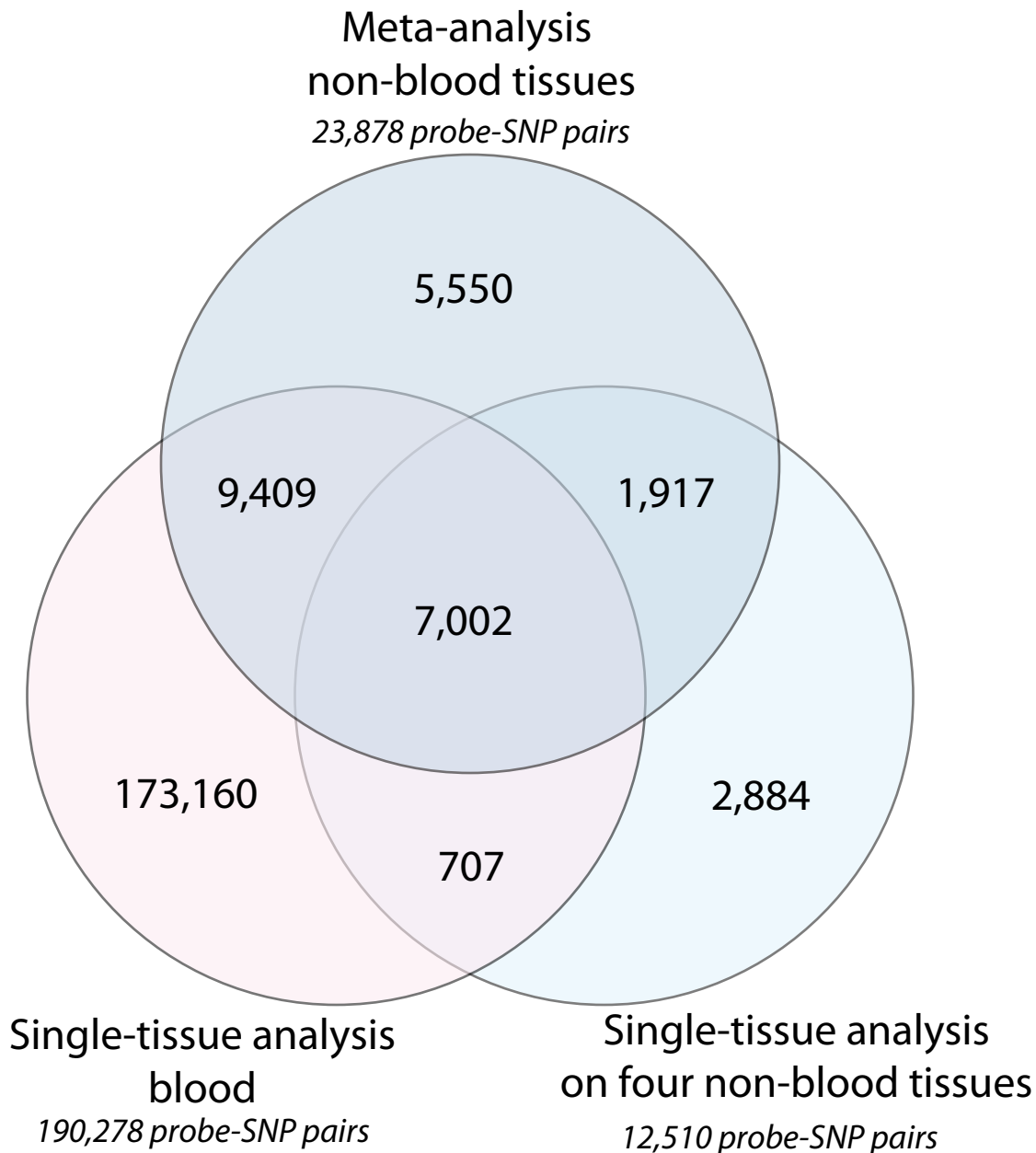

Supplement: Figure S4 — Overlap of the associated probe-SNP pairs across the single-tissue analysis and meta-analysis. (PDF) [file pgen.1002431.s004.pdf]

Density Plot

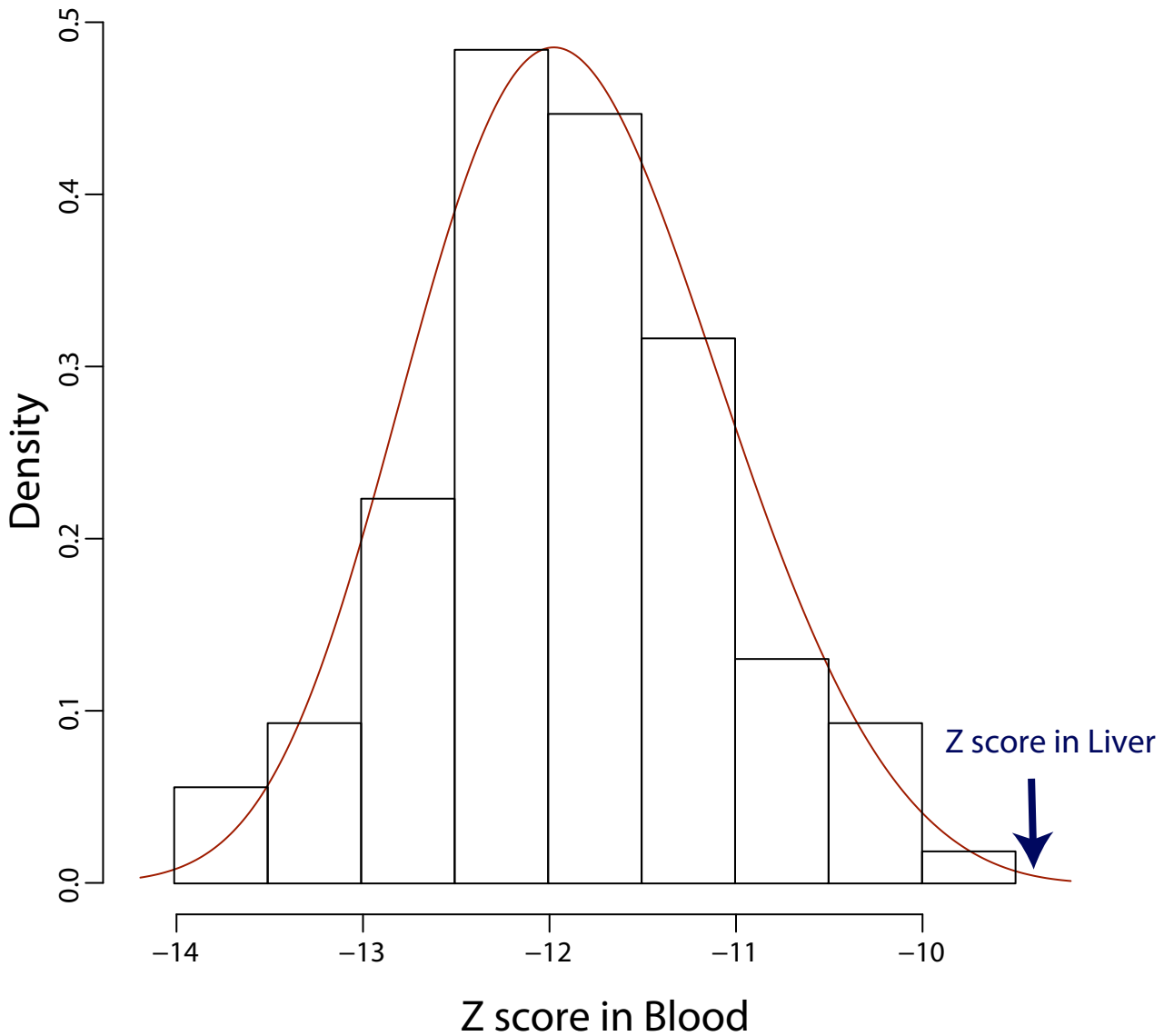

Supplement: Figure S5 — Sampling procedure. We assessed the difference of association strength between blood and four other tissues (liver, SAT, VAT and muscle). As an example, for liver, we randomly sampled 74 subjects out of the 1,240 blood subjects (making the same sample size as for the liver tissue dataset) and re-measured the association strength for each significantly associated probe-SNP pair, in terms of Z-scores. This sampling procedure was repeated 100 times. The histogram showed the Z-scores distribution of a certain cis-eQTL in 74 blood subjects. We then assessed the deviation of the Z-scores detected in liver (the red arrow) from the distribution of Z-scoress in blood, by fitting the extreme value distribution (EVD) (the red line). The same analysis was performed for comparing blood with SAT, VAT and muscle, by randomly sampling N number of blood subjects (N = 83 for the SAT sample size; 77 for the VAT sample size, and 62 for the muscle sample size, respectively). (PDF) [file pgen.1002431.s005.pdf]

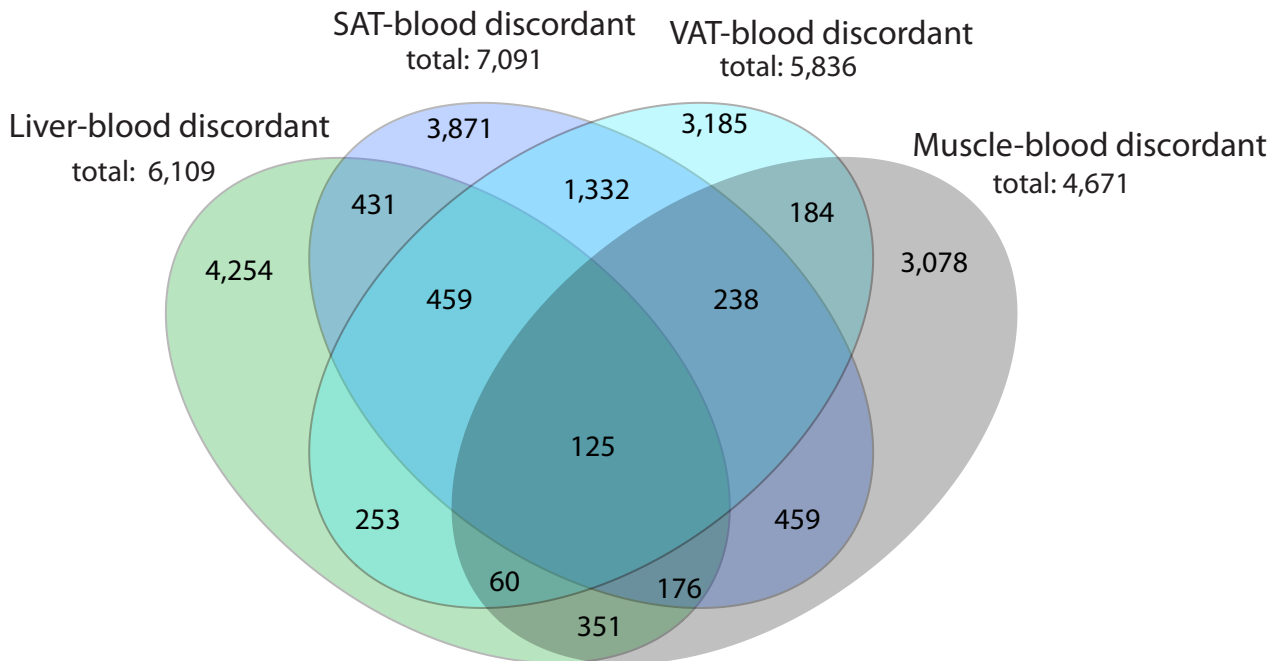

Supplement: Figure S6 — The overlap of discordantly associated probe-SNP pairs. (PDF) [file pgen.1002431.s006.pdf]

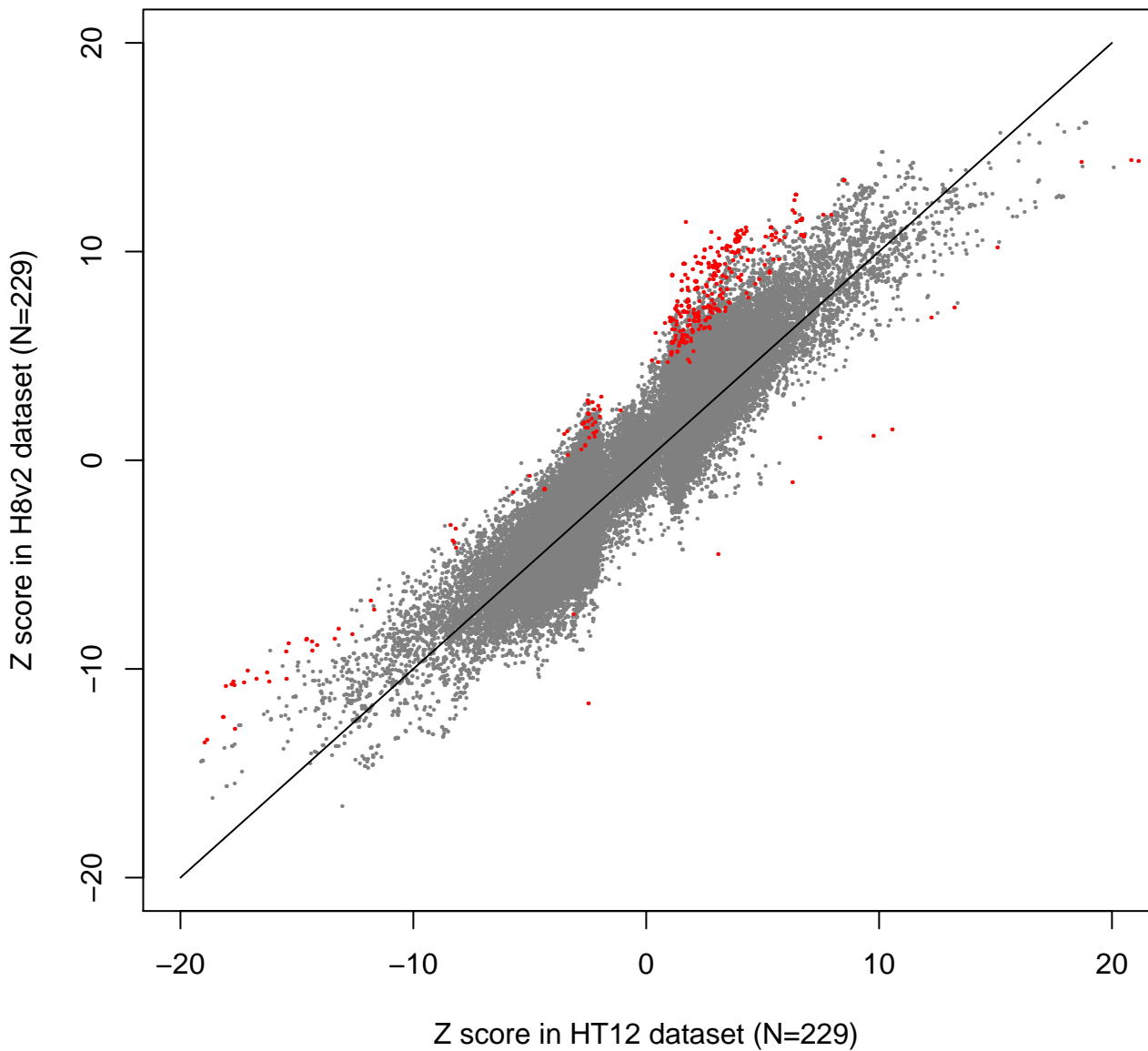

Supplement: Figure S7 — The comparison of Z-scores between two independent blood datasets. The comparison of cis-eQTL effect was confined to the set of 93,656 probe-SNP pairs that have been tested in two independent blood datasets, e.g., a discovery set of 1,240 subjects profiled on the Illumina HT12 expression platform (HT12) and a validation set of 229 subjects profiled on the Illumina H8v2 expression platform (H8v2). The Z-scores of cis-eQTL in the discovery set were the mean of Z-scores from 100× taking a sample of 229 out of the 1,240 blood subjects. The gray dots indicate the concordantly associated probe-SNP pairs between the two blood samples. The red dots indicate the discordantly associated probe-SNP pairs (the false-positive tissue-dependent association). The black line is the diagonal line. (PDF) [file pgen.1002431.s007.pdf]

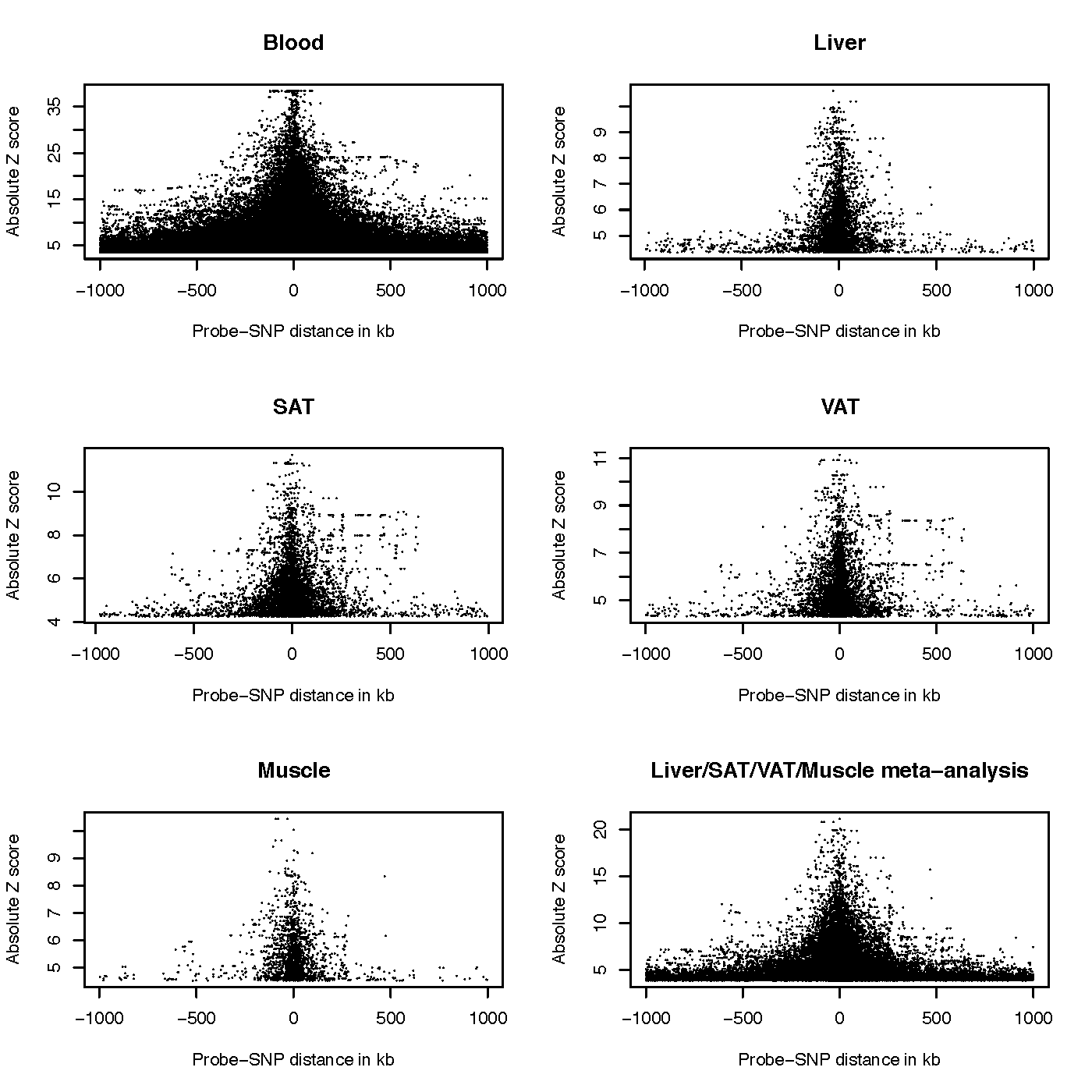

Supplement: Figure S8 — The probes-SNP distance for associated probe-SNP pairs. The distance was calculated by the base pair position (bp) of SNPs minus the bp position of the middle point of the probes. (PNG) [file pgen.1002431.s008.png]

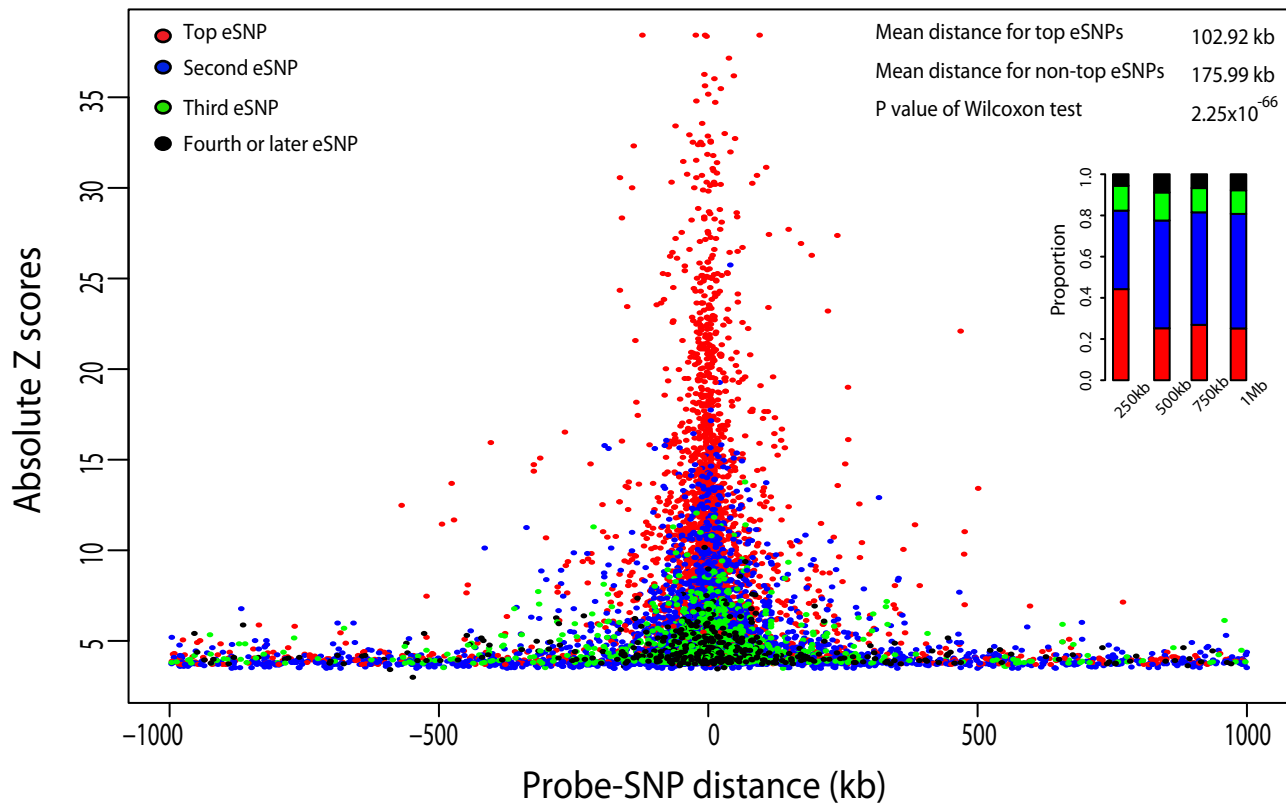

Supplement: Figure S9 — Probe-SNP distance for 2,794 eProbes in blood with multiple independent eSNPs. (PDF) [file pgen.1002431.s009.pdf]

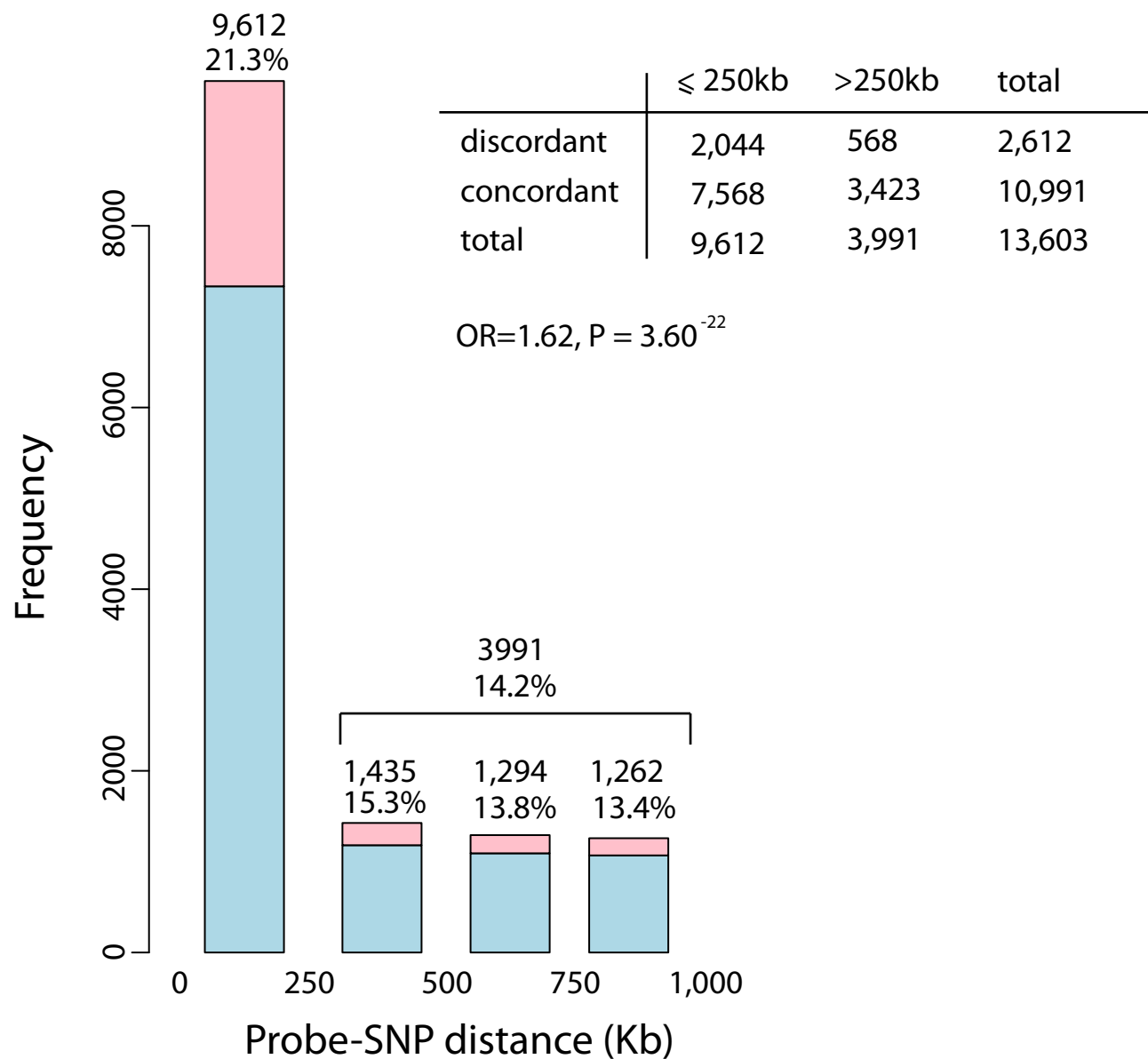

Supplement: Figure S10 — The discordant probe-SNP pairs vs. the probe-SNP distance. The histogram shows the number the probe-SNP pairs with different distance. The numbers on each bar show the total number of probe-SNP pairs and the percentage of pairs with discordant association. The 2×2 table for Fisher's exact test is shown. (PDF) [file pgen.1002431.s010.pdf]

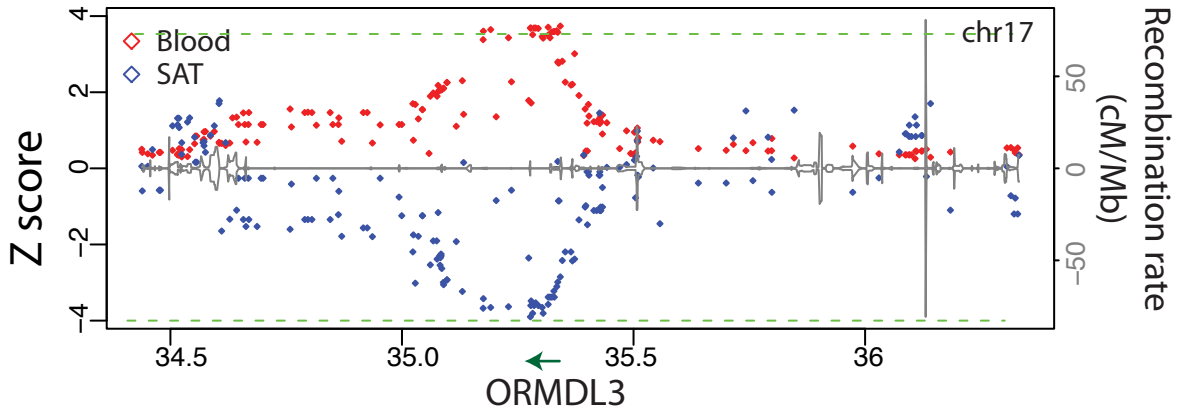

Supplement: Figure S12 — The opposite association of ORMDL3 gene between blood and SAT. The x-axis is the genome position based on genome build 36.3 (in Mb). The y-axis at the left is the association profiles in terms of Z-scores. The Z-scores in blood, represented as the red dots, has been weighted by the square root of the sample size, corresponding to the compared tissue. The blue dots represent the Z-scores in SAT. The dashed green line indicates the significance level at FDR 0.05. For a better illustration of allelic direction, we assigned the association Z-scores in blood a positive value. If the allelic direction in SAT is the same as that in blood, the Z-scores in SAT are positive too; otherwise, the Z-scores in SAT are negative. (PDF) [file pgen.1002431.s012.pdf]

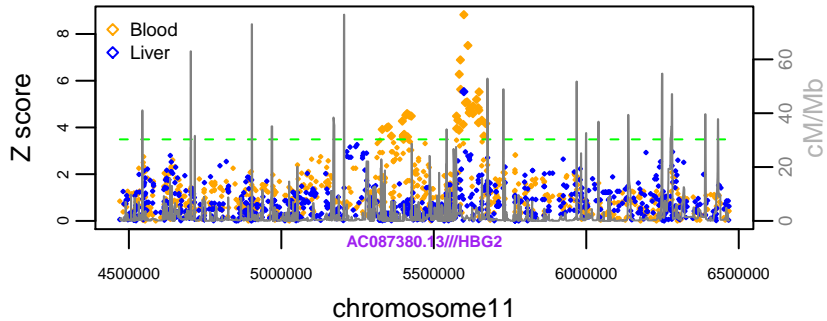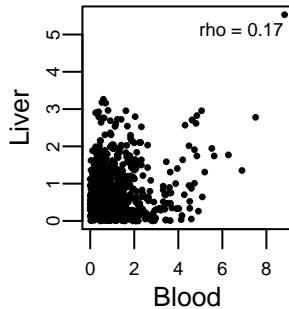

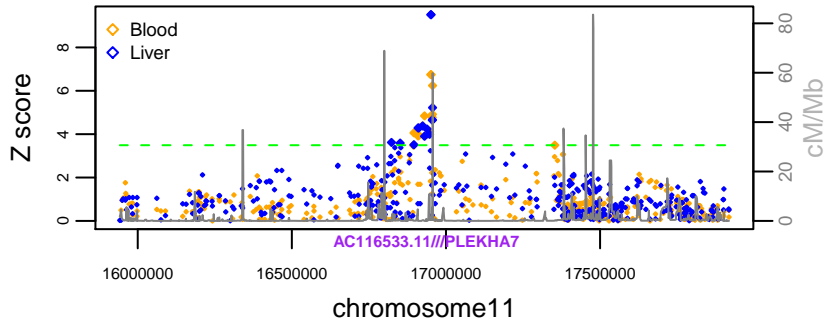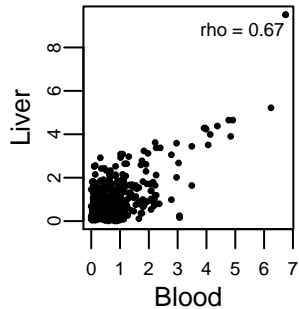

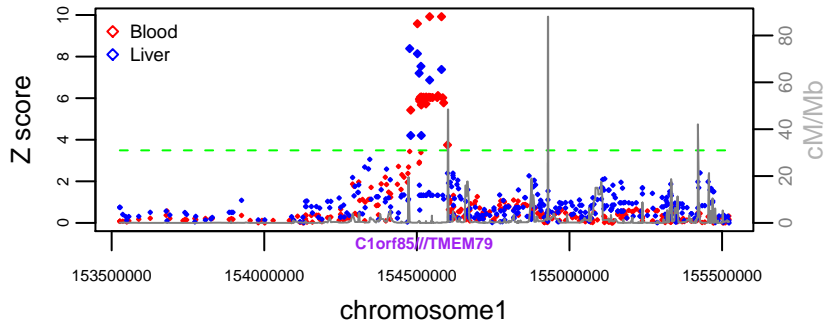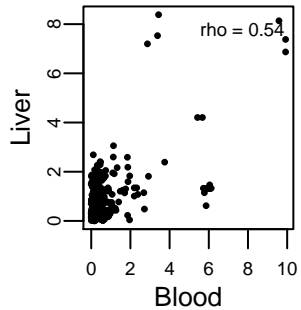

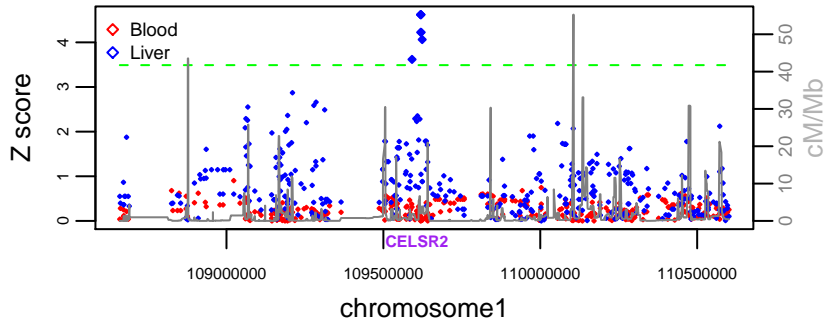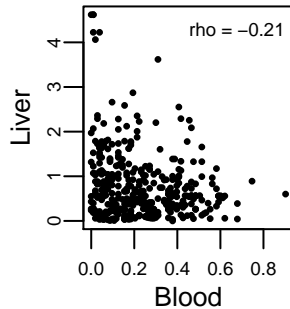

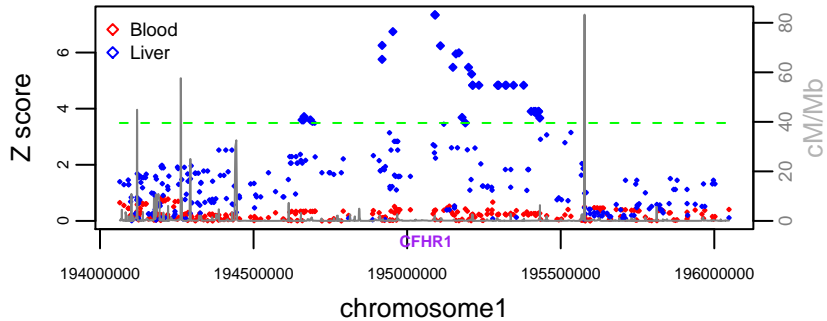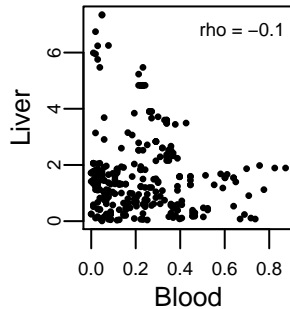

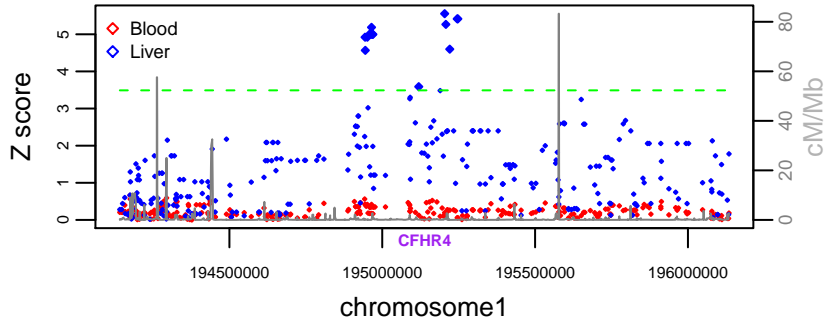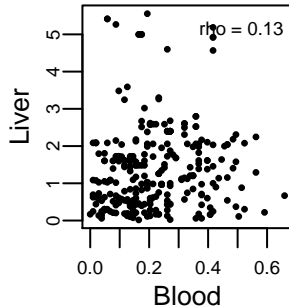

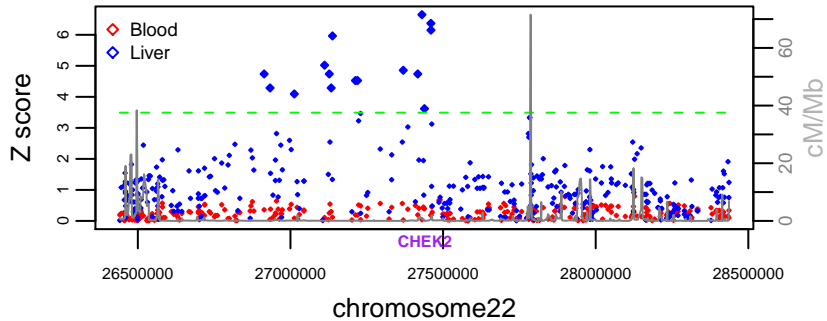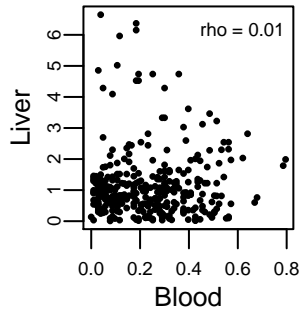

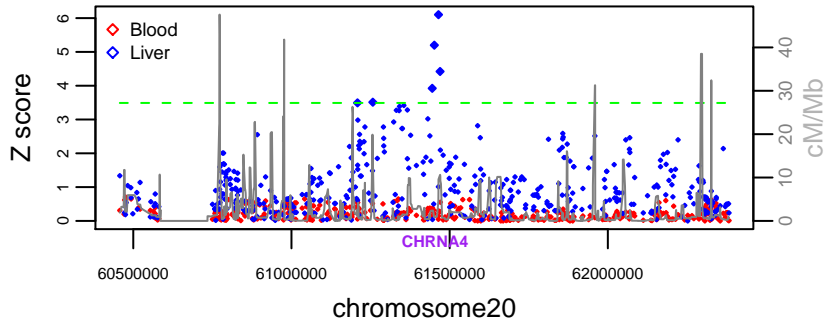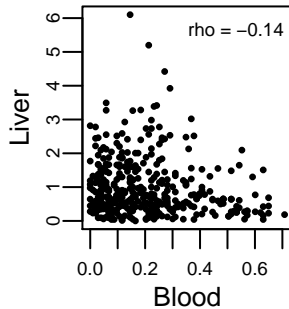

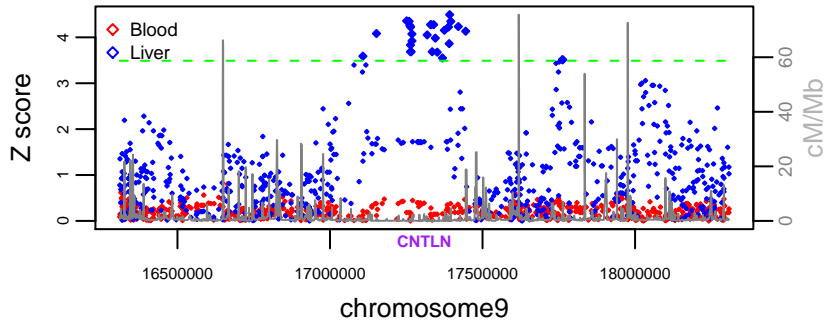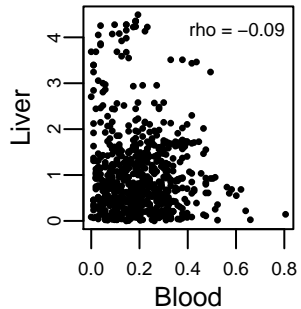

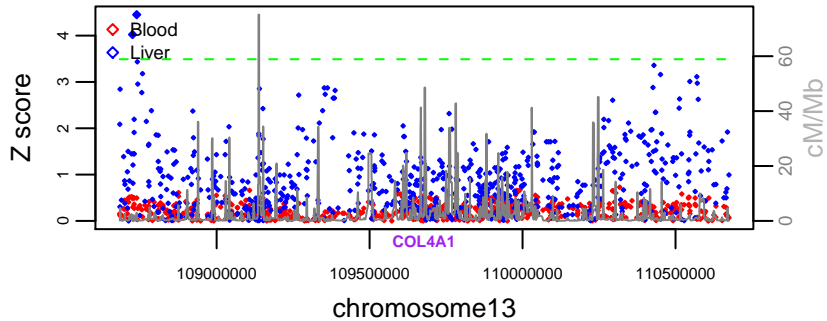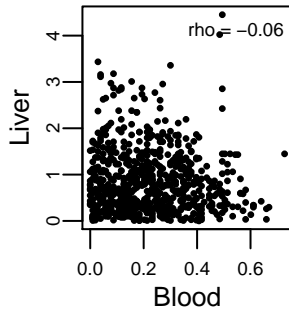

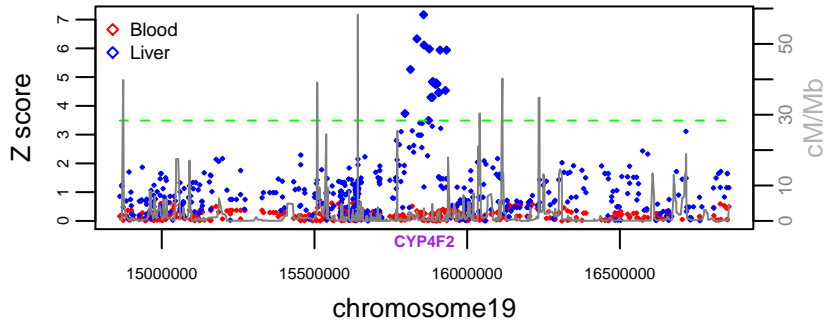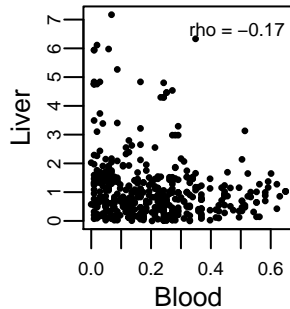

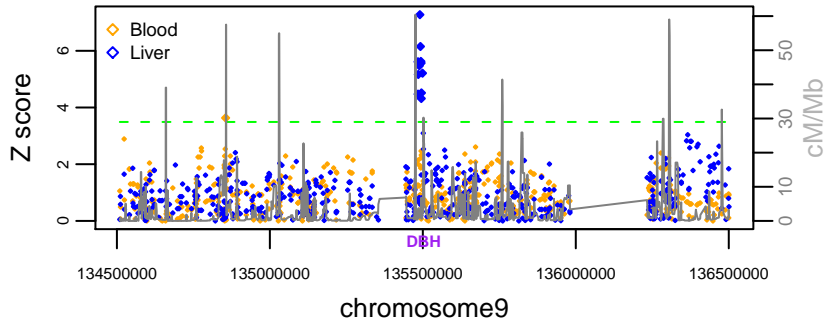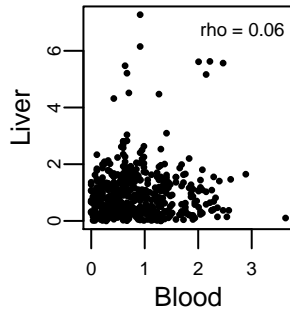

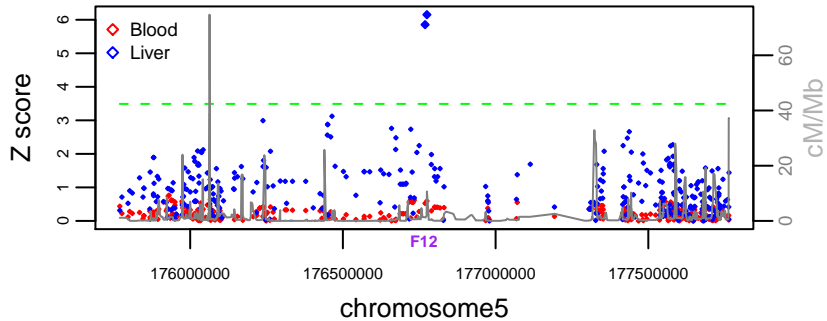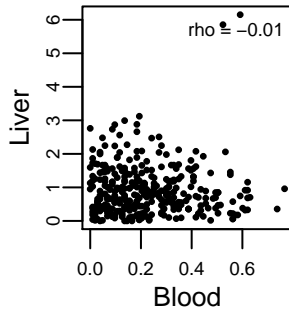

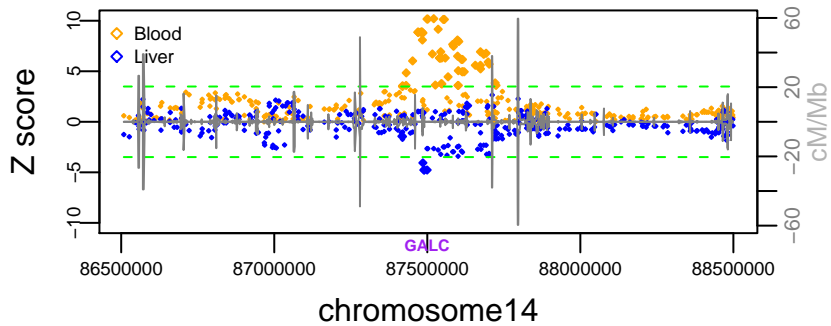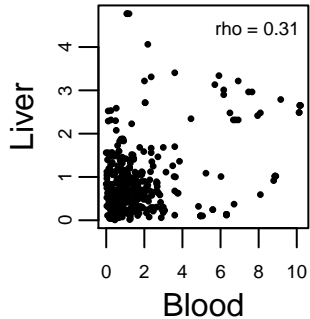

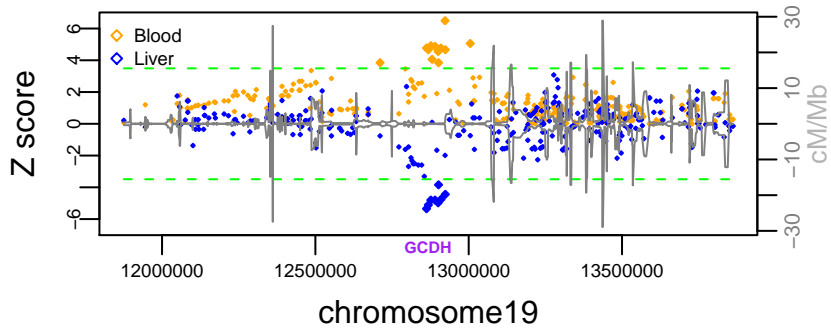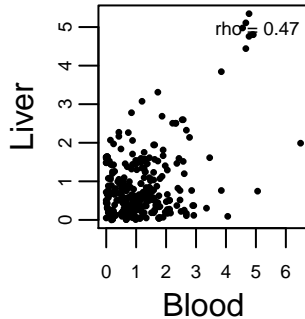

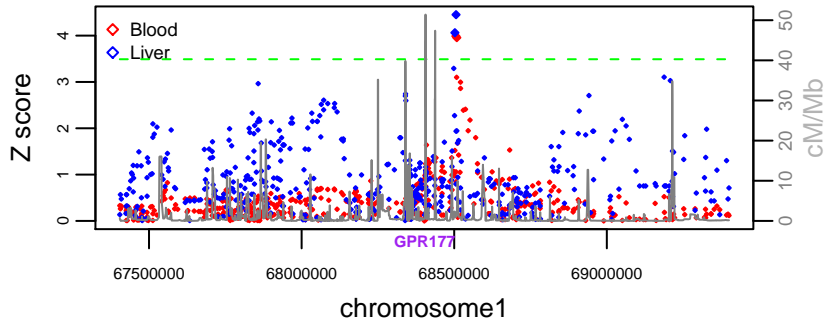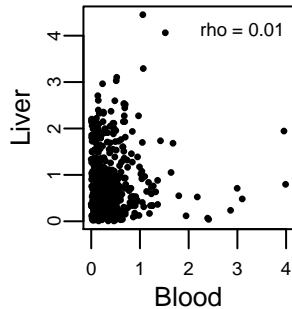

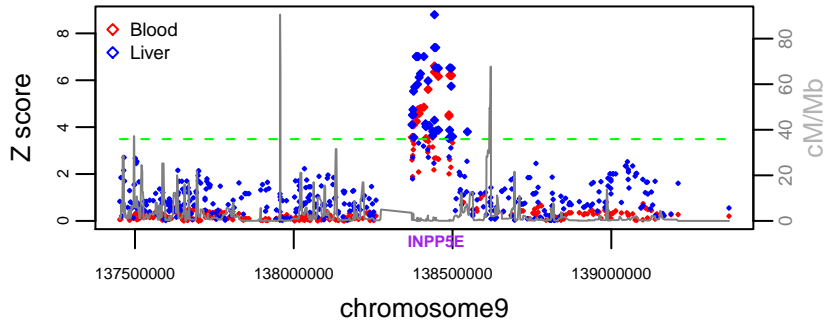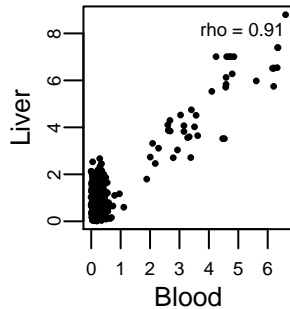

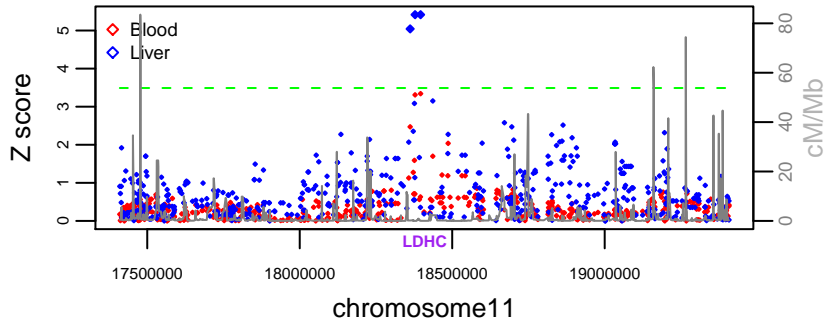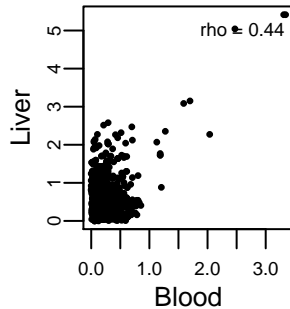

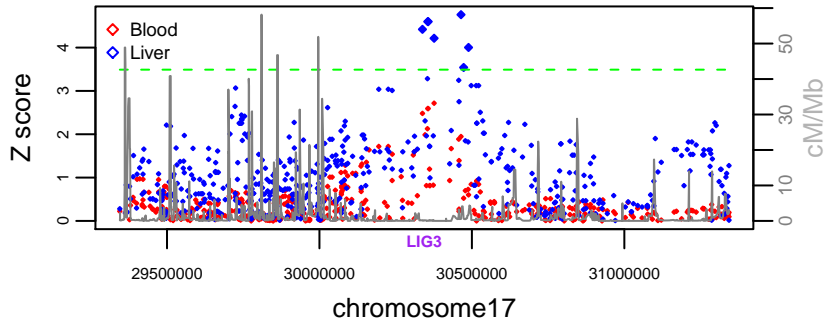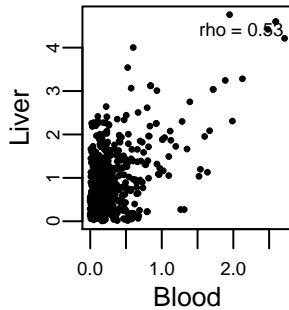

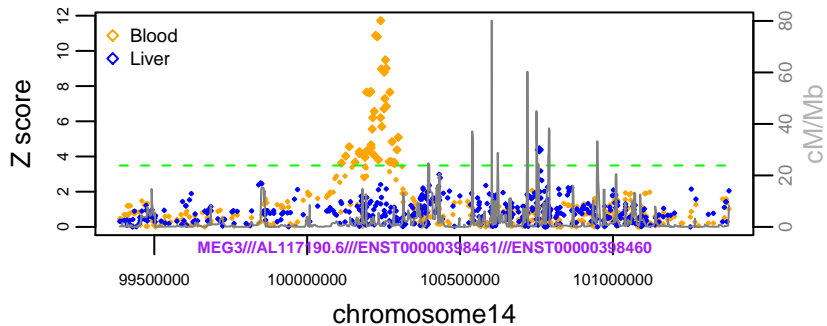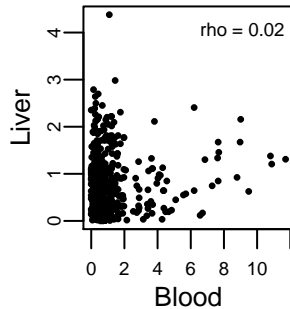

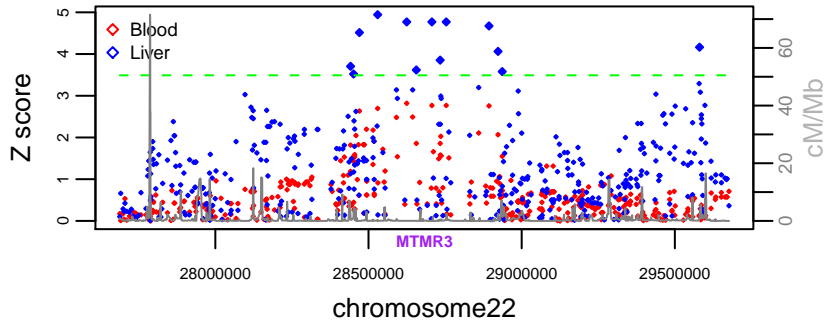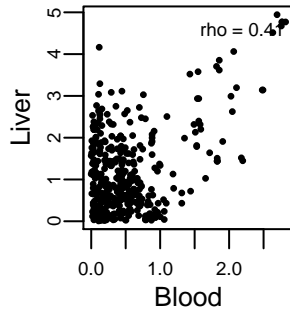

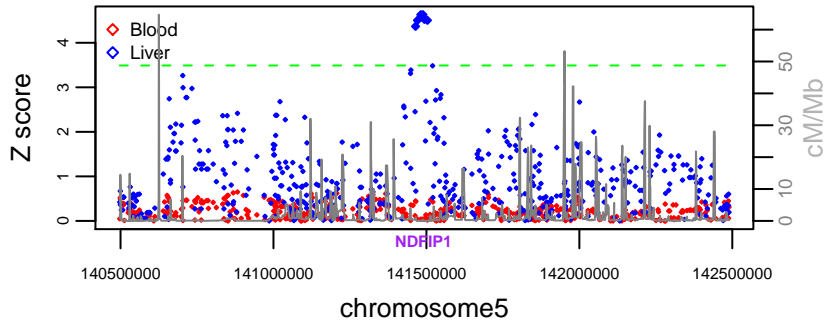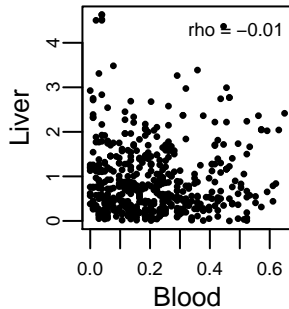

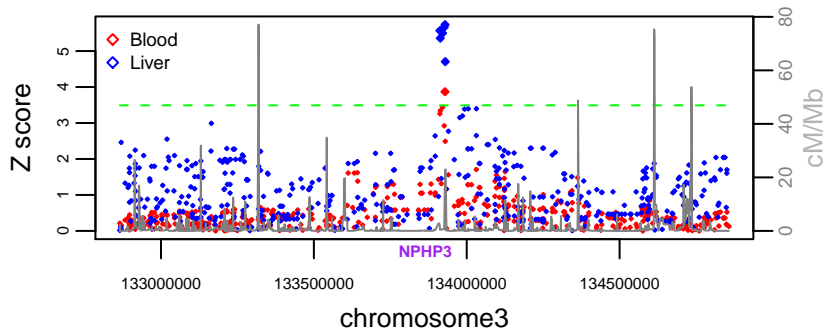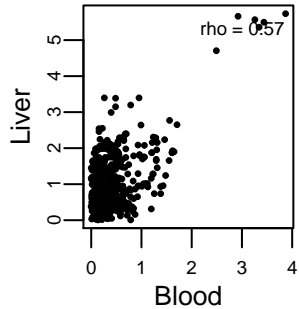

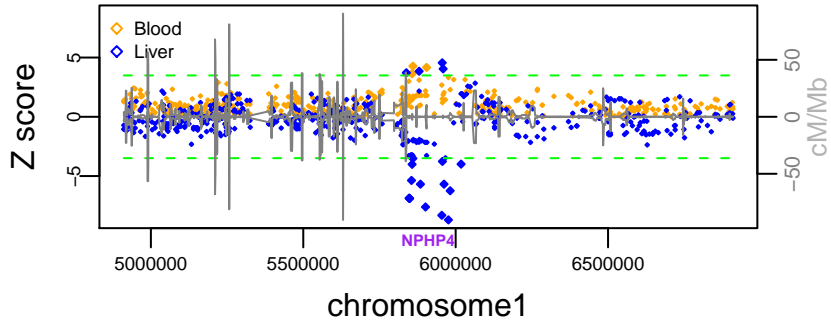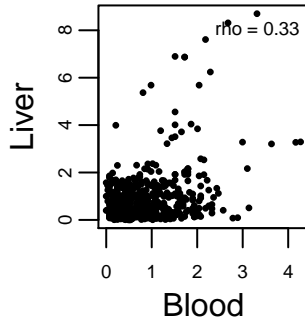

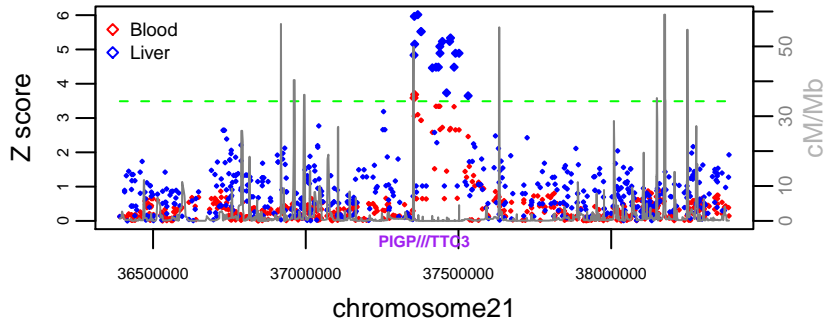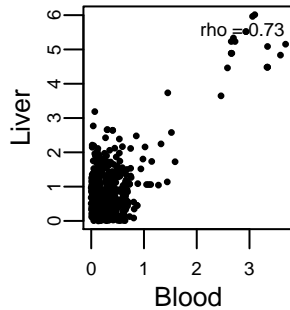

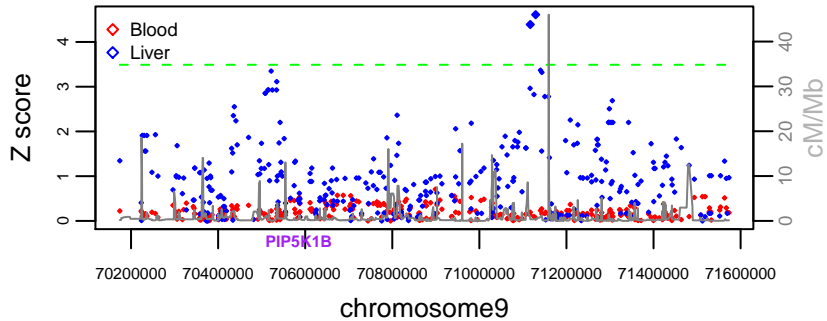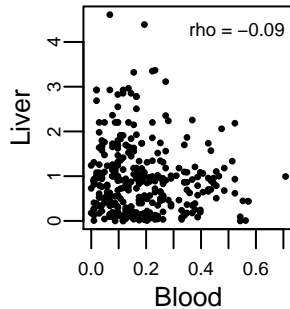

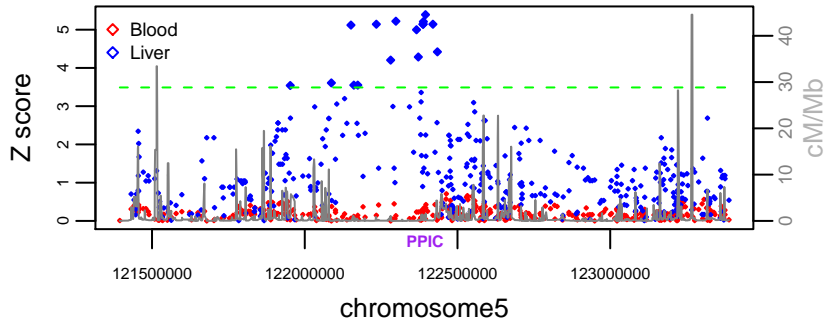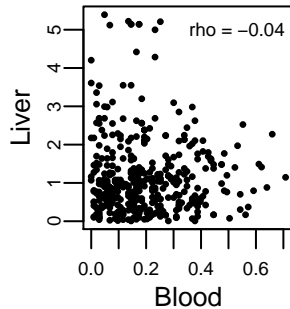

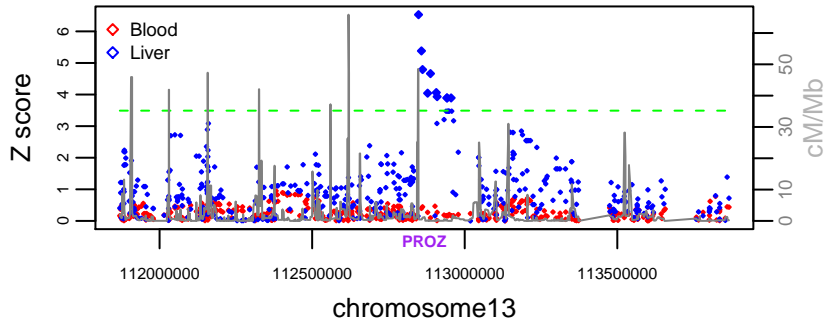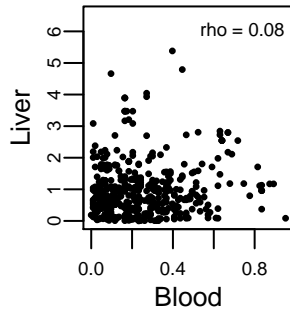

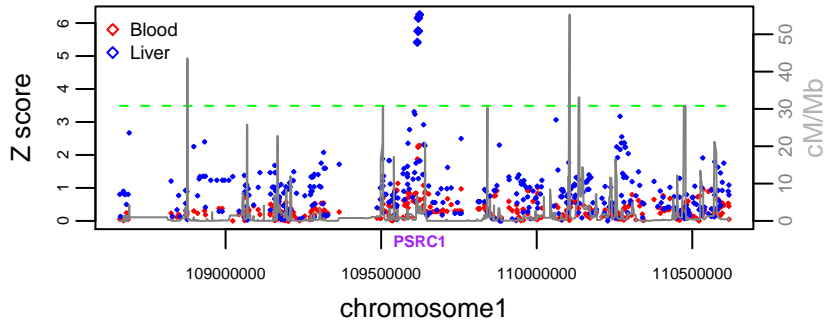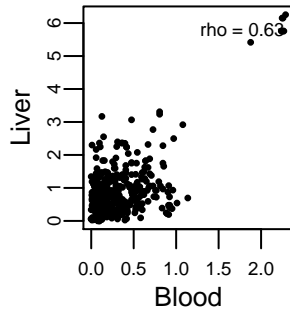

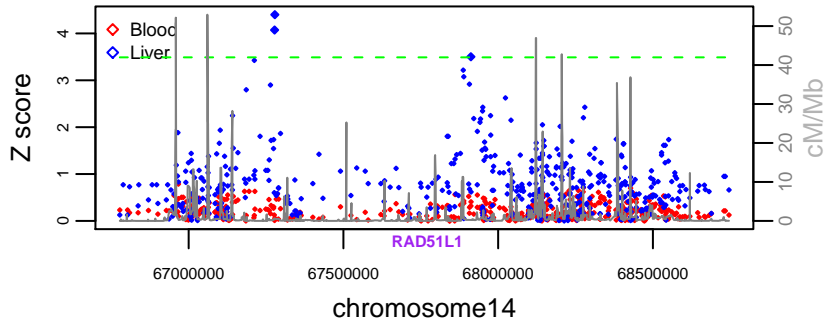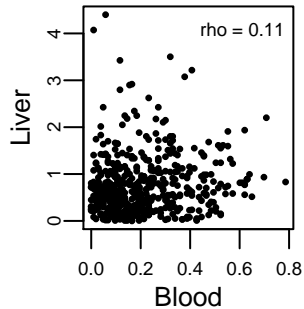

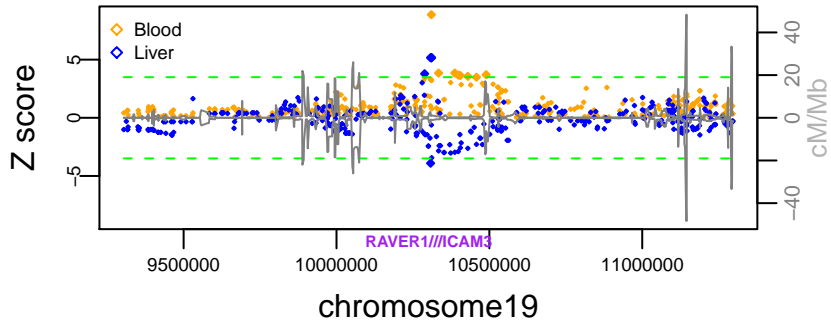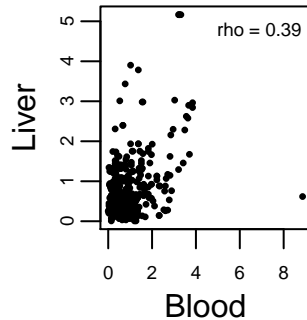

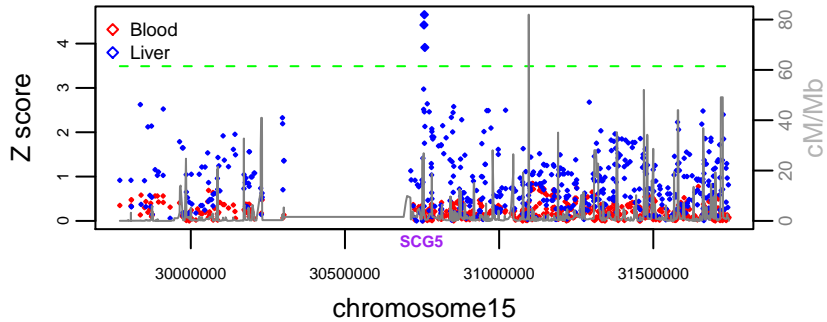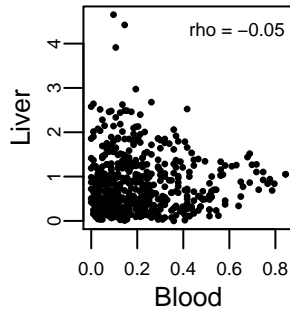

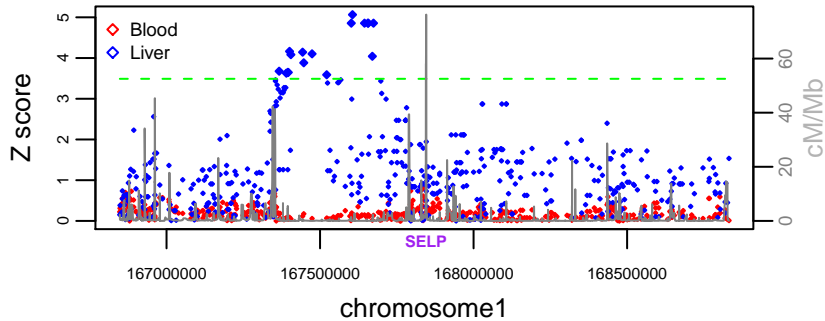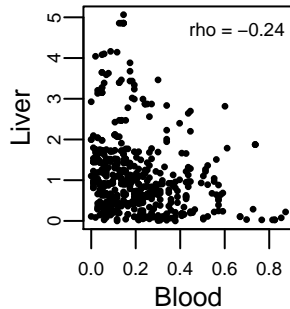

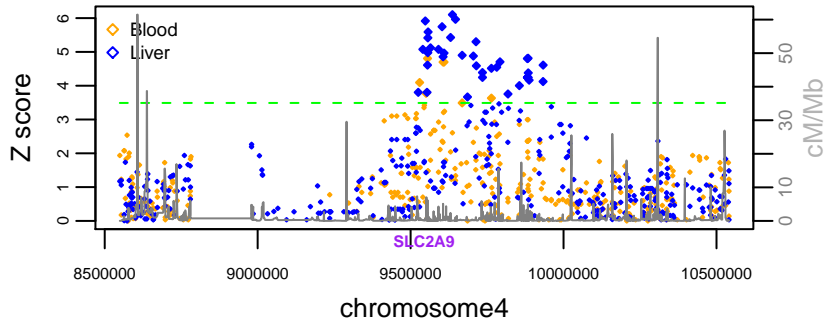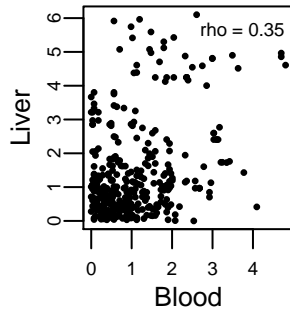

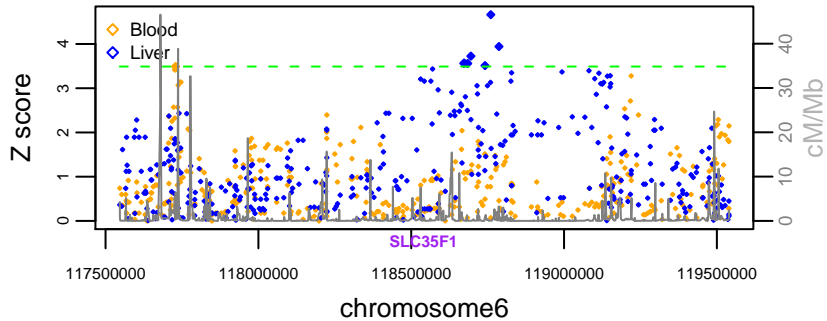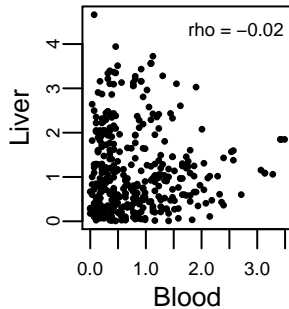

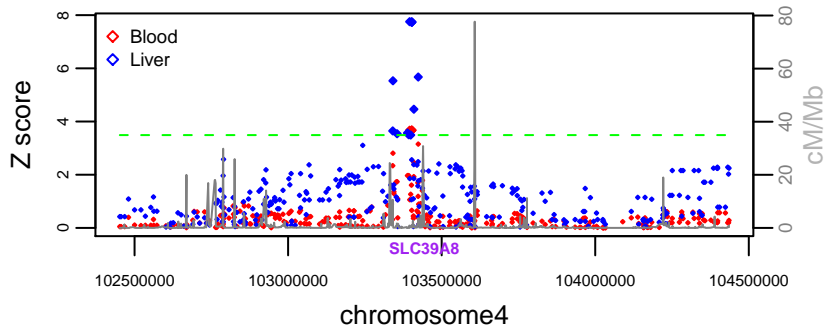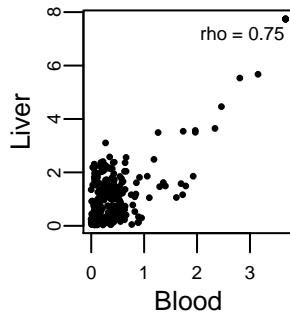

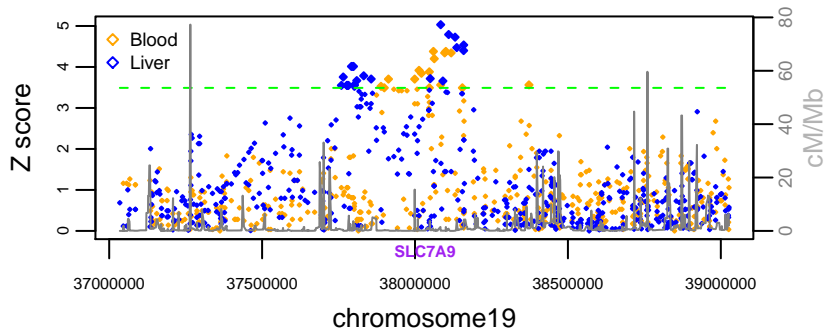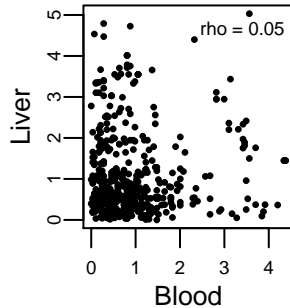

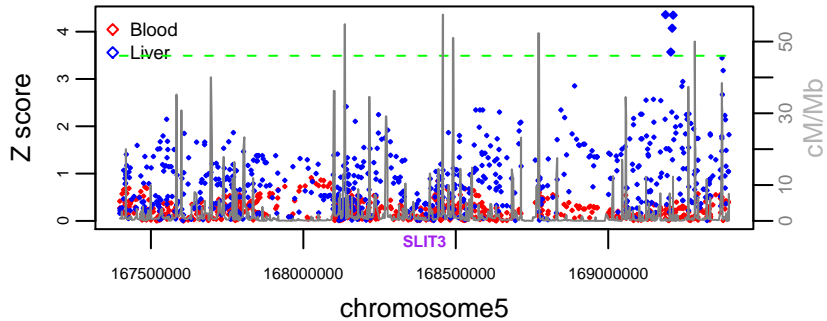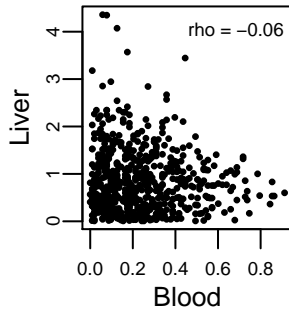

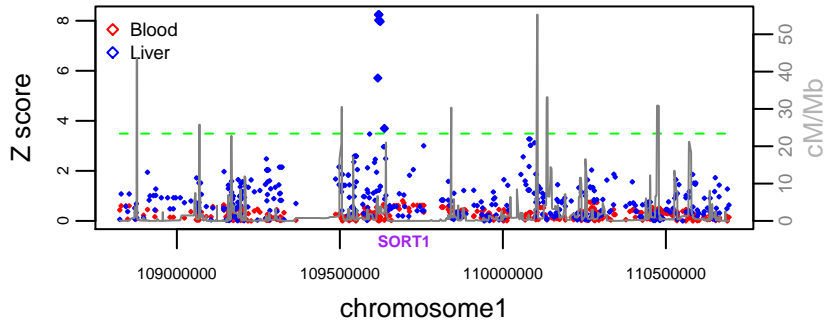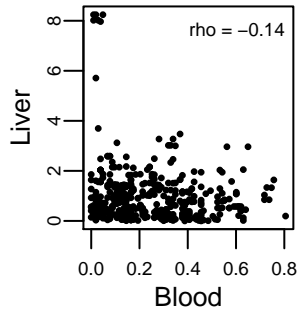

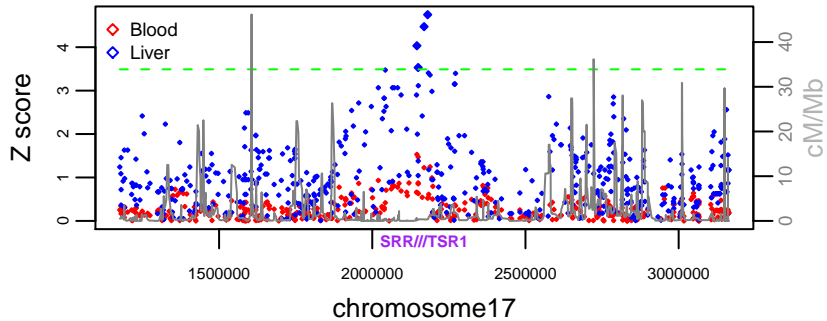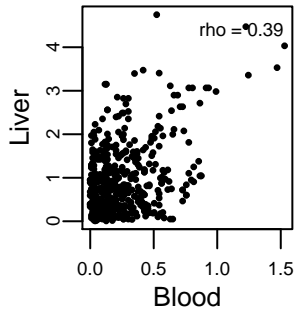

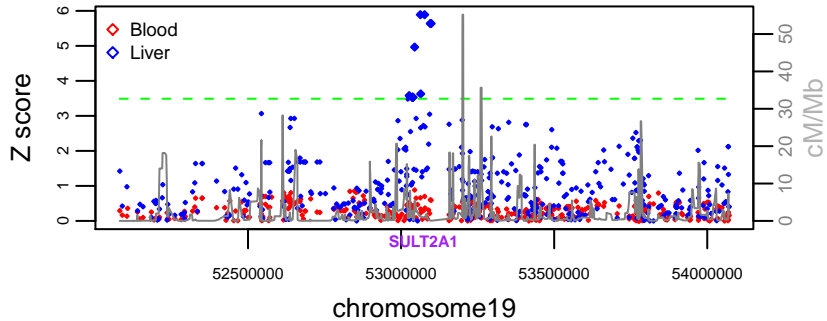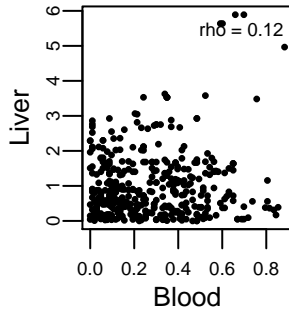

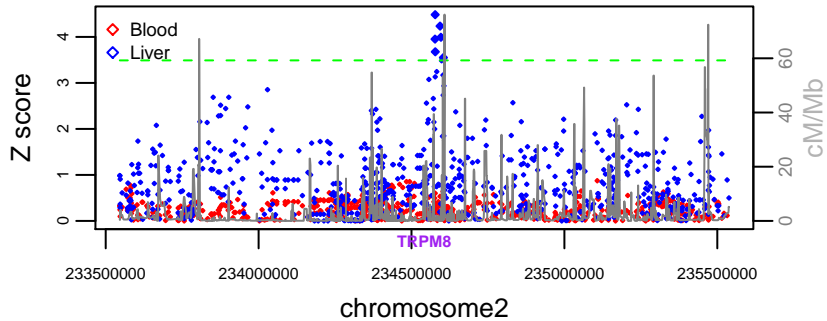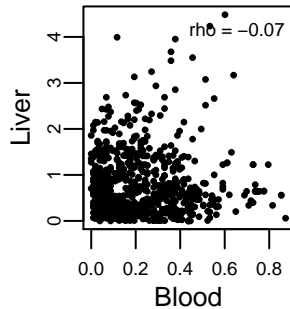

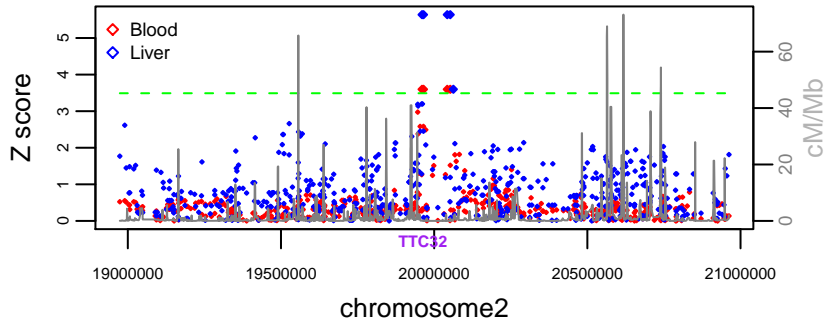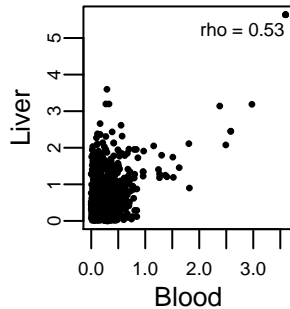

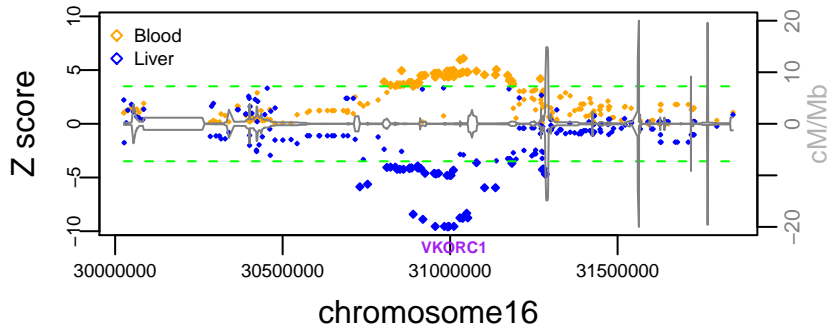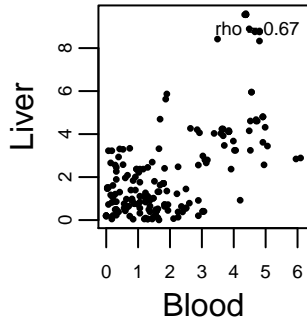

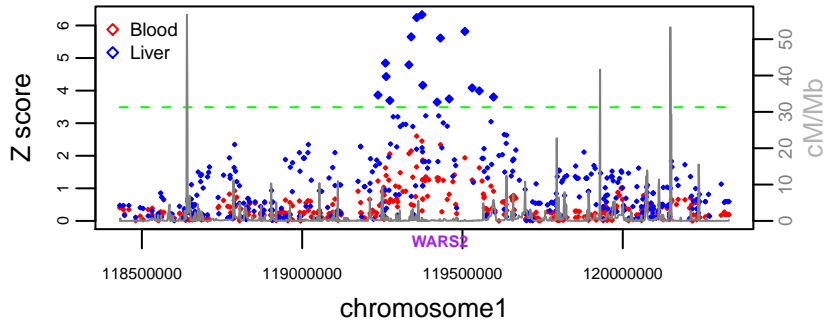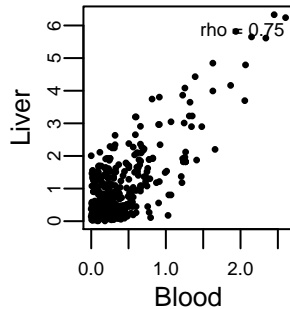

Supplement: Figure S13 — The association profiles of the selected trait-associated genes that show discordant association between blood and liver. The x-axis is the genome position based on genome build 36.3. The y-axis at the left is the association profiles in terms of the Z-score. The Z-score in blood, represented as the red dots or orange dots. The red dots refer to the Z-scores that have been weighted by the square root of the sample sizes, corresponding to the compared tissue. For the clarity of subtle effect in blood, the weak association in blood was shown as orange dots if the Z-scores have not been weighted by the sample size, i.e., the Z-scores reported in 1,240 subjects. The blue dots represent the Z-scores in liver. The dashed green line indicates the Z-score 3.49, representing the significance level in blood at FDR 0.05. The right panel shows the correlation of the absolute association Z-scores between two tissues. The rho-value indicates the correlation coefficient of the Pearson correlation. (PDF) [file pgen.1002431.s013.pdf]

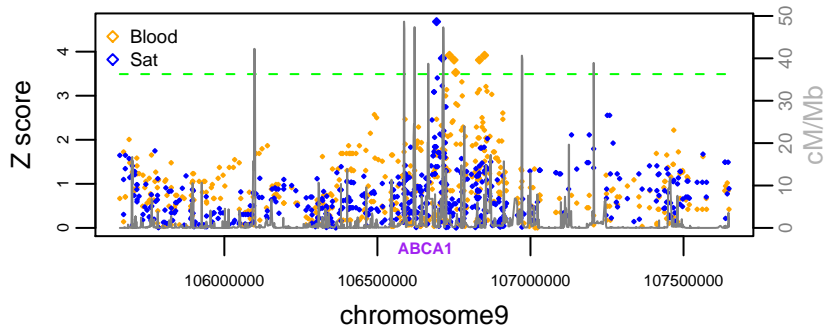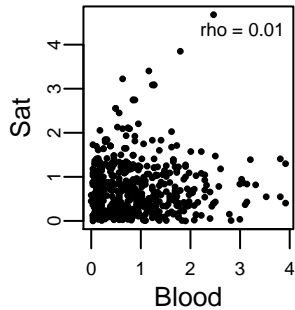

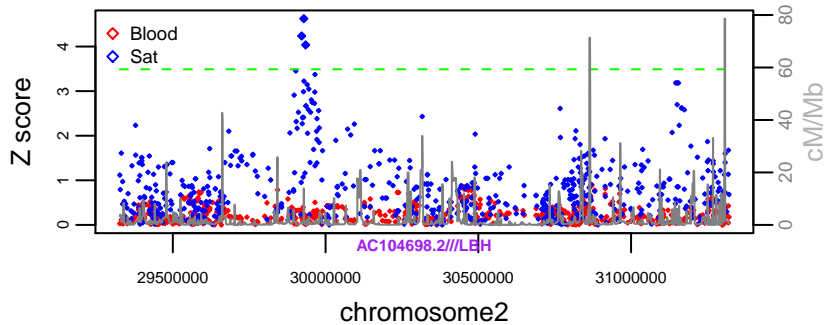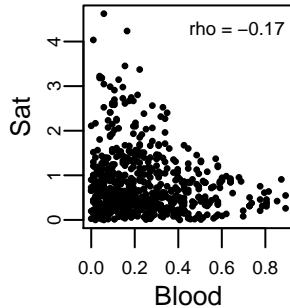

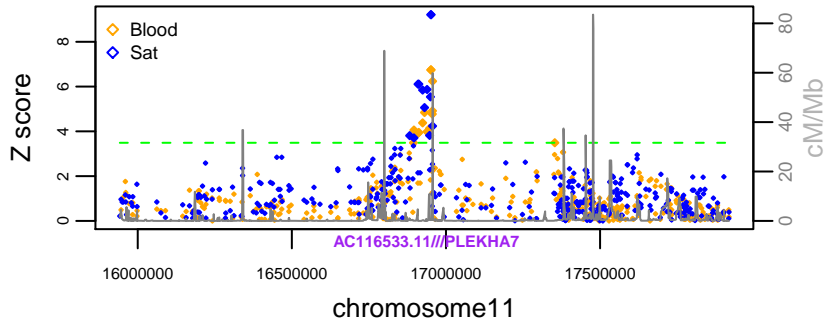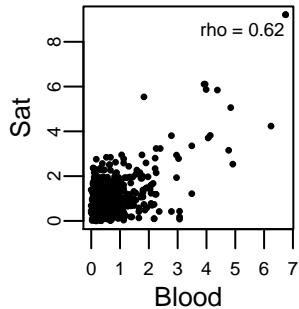

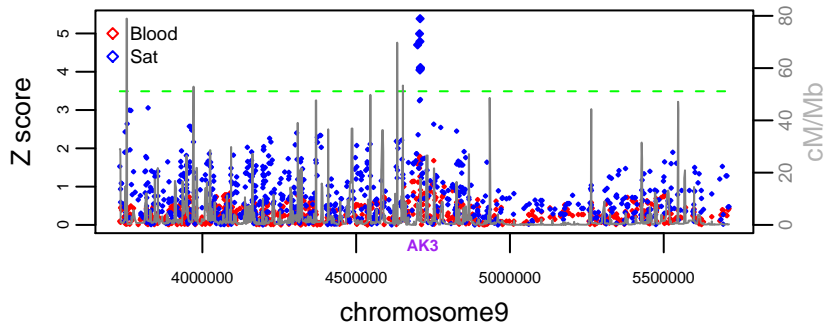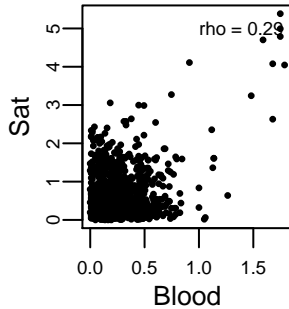

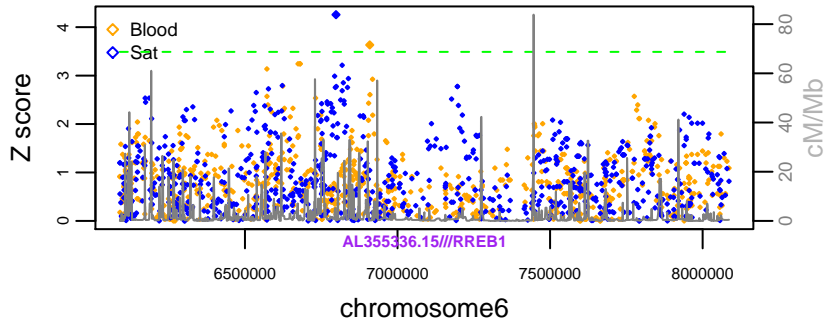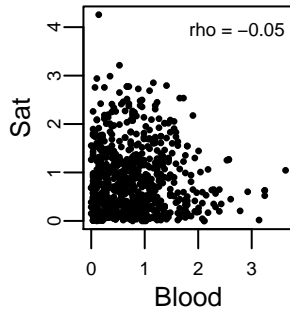

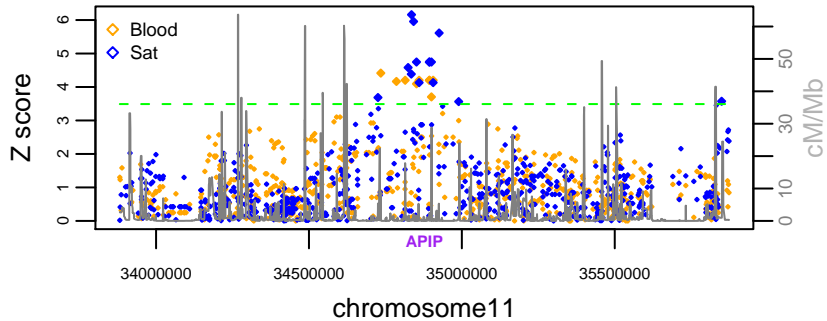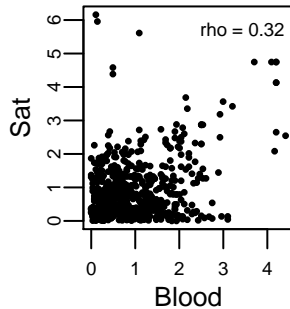

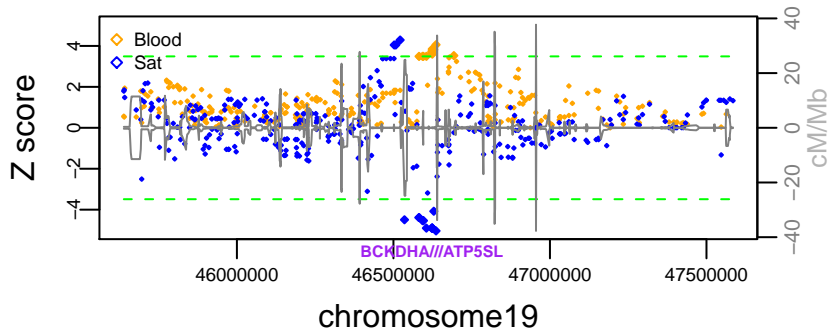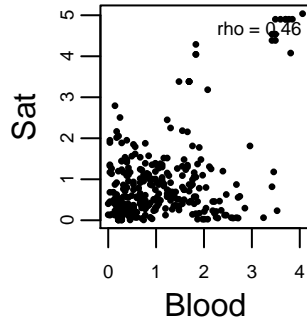

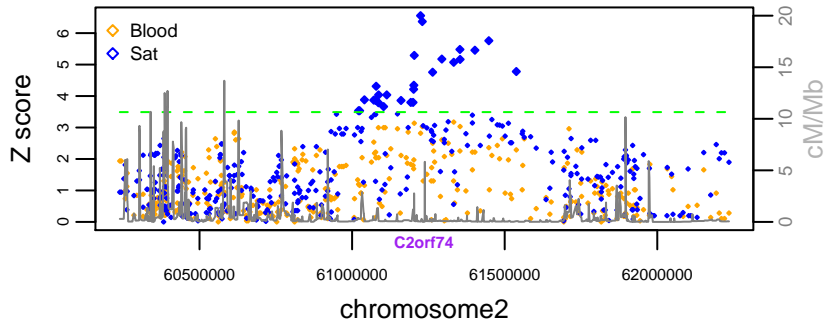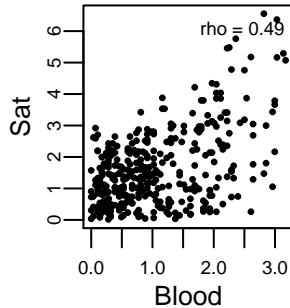

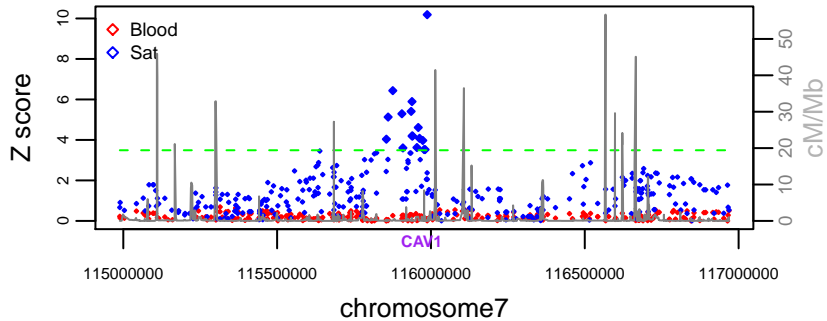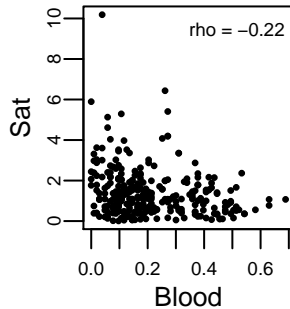

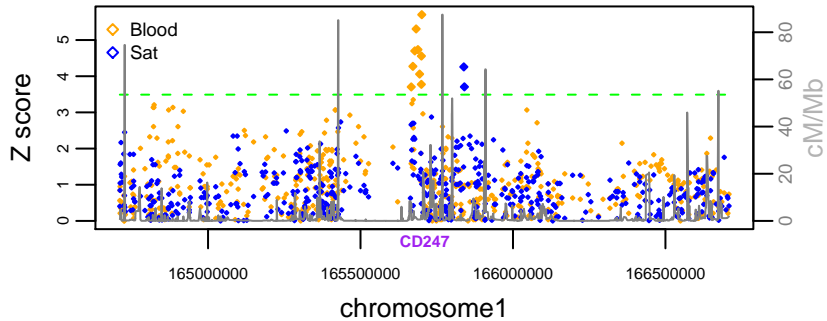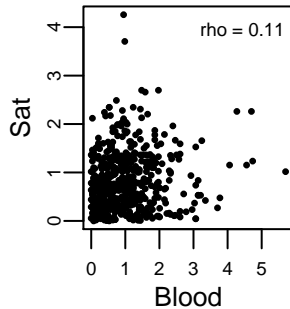

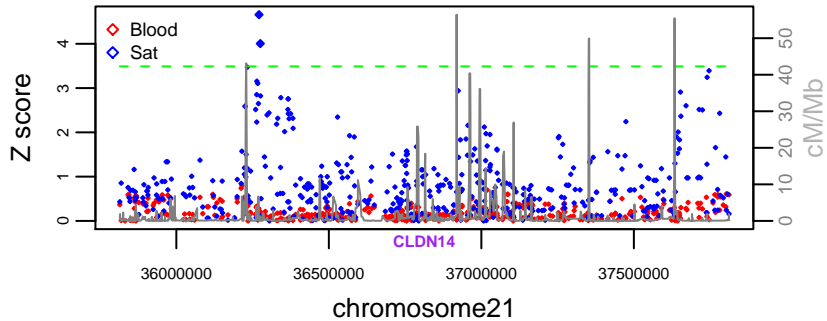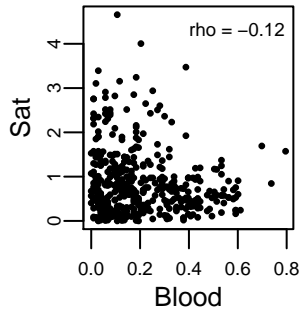

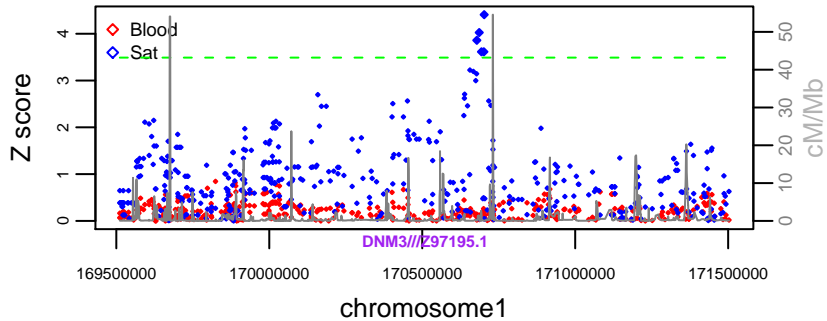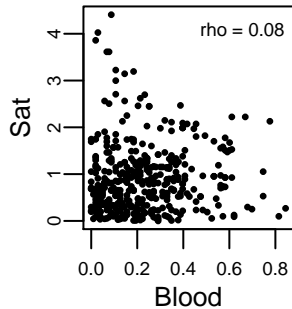

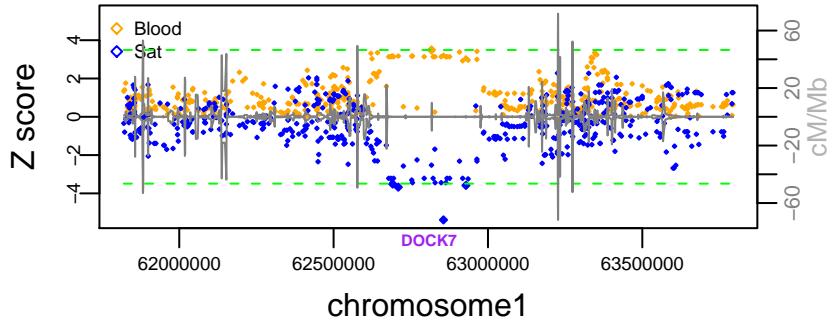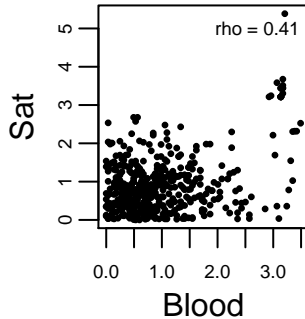

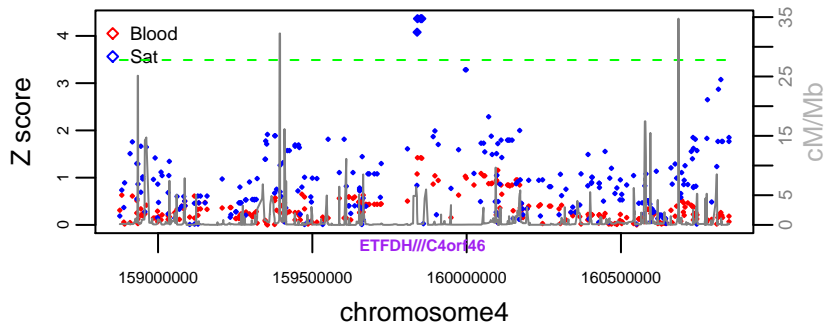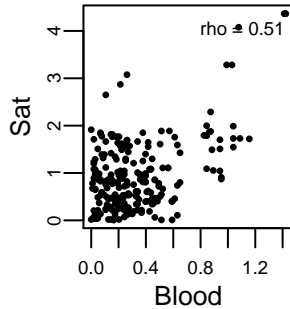

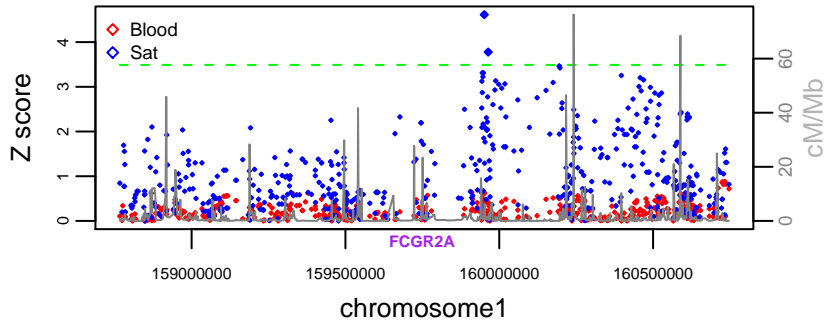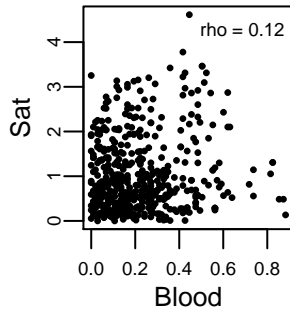

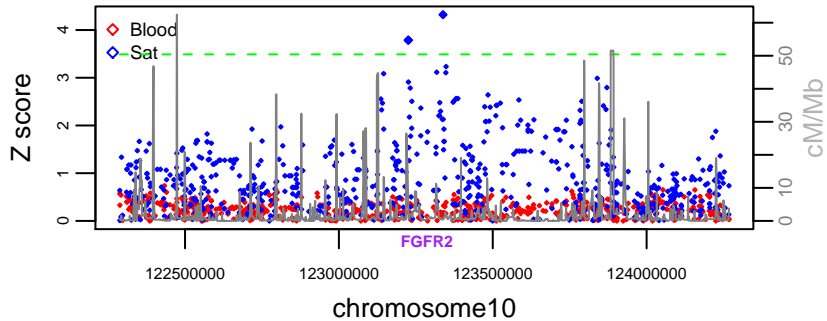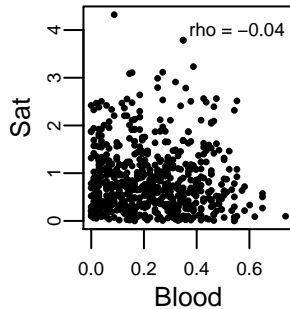

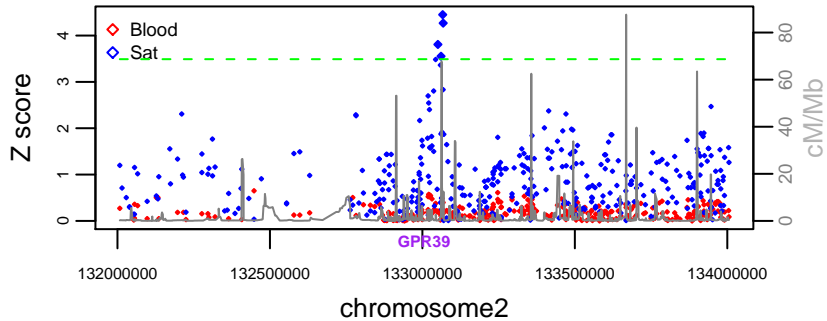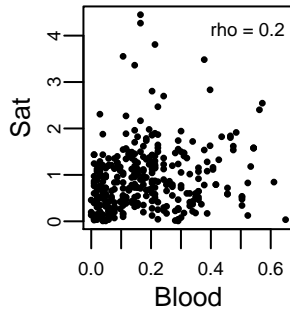

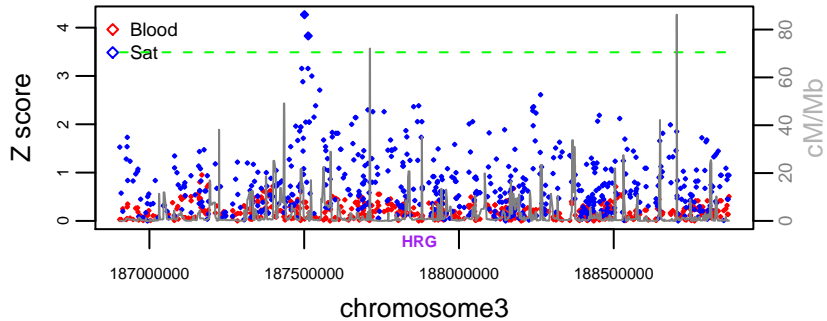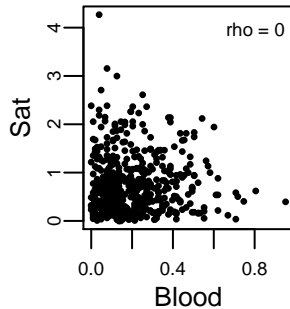

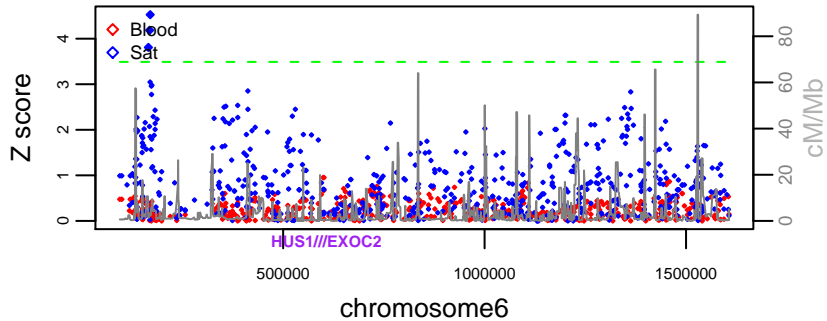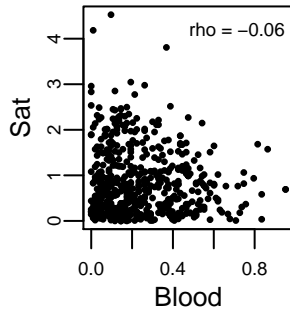

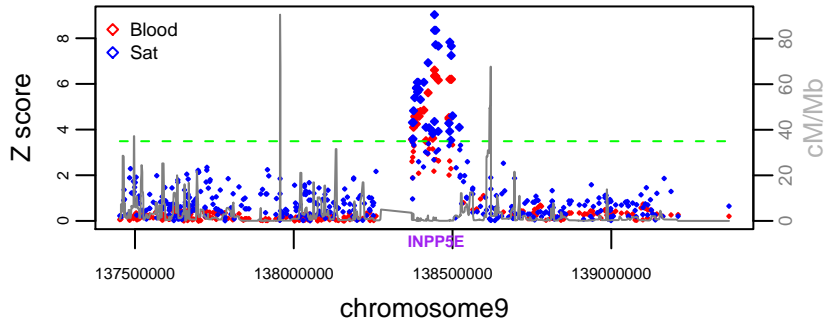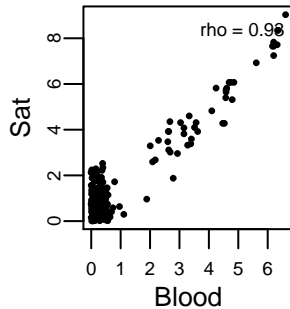

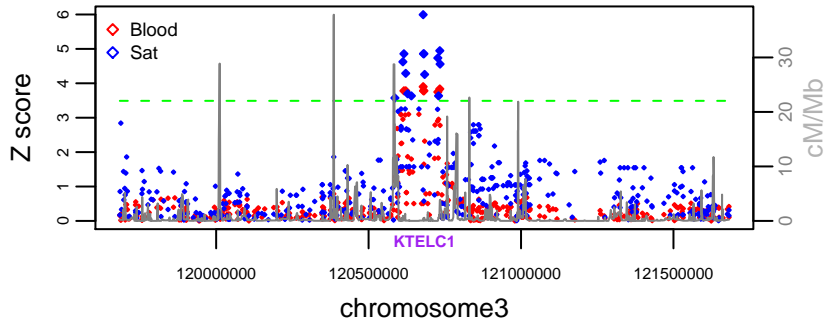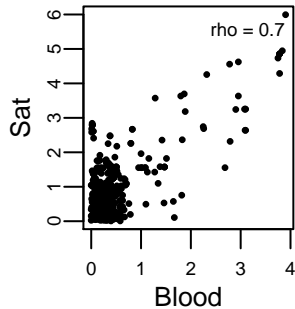

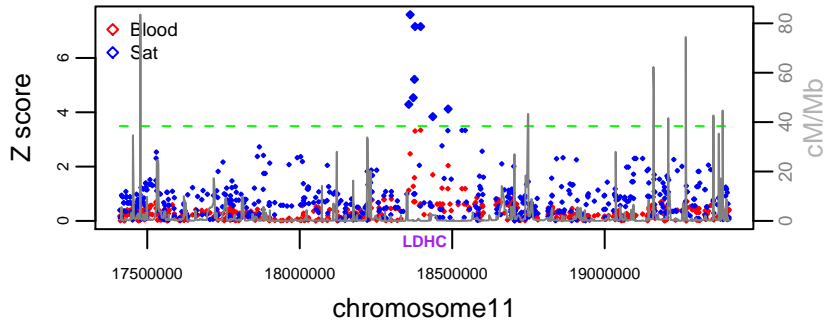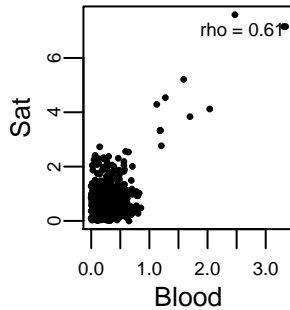

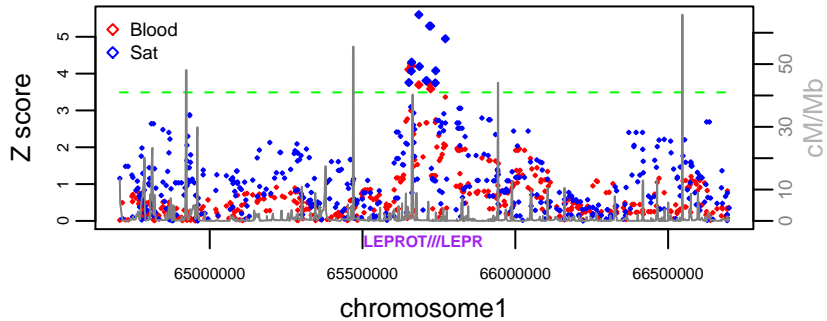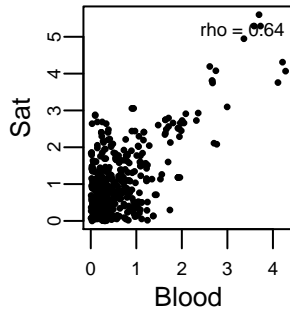

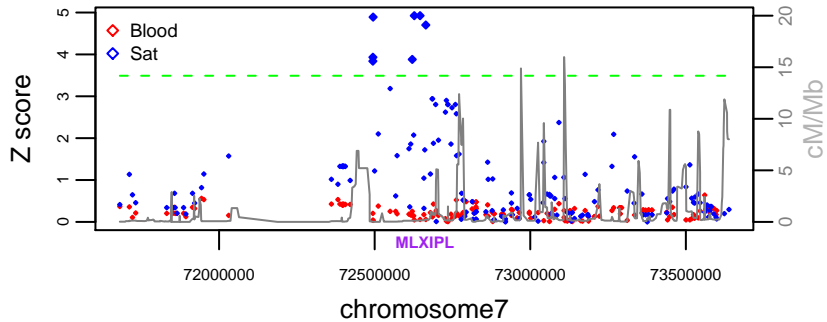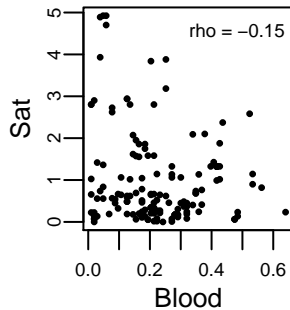

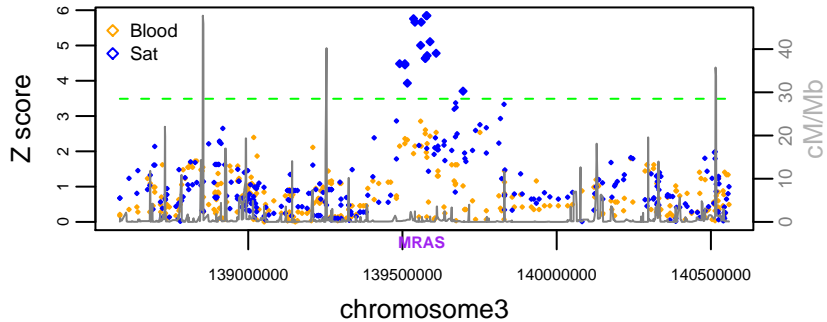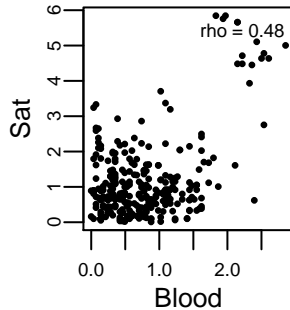

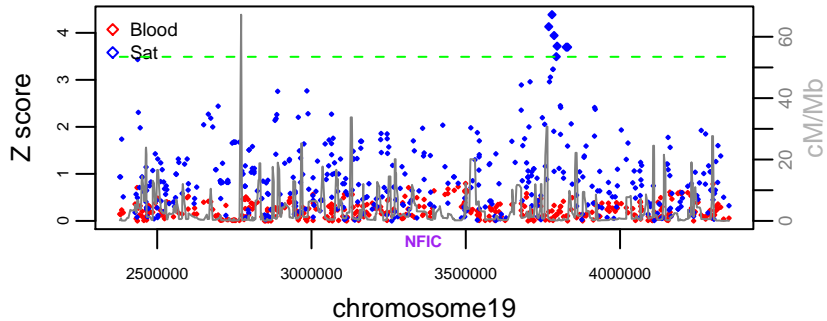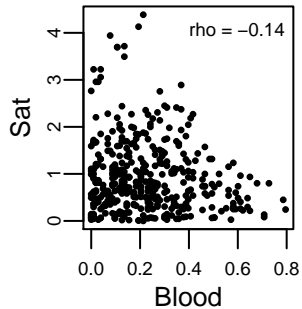

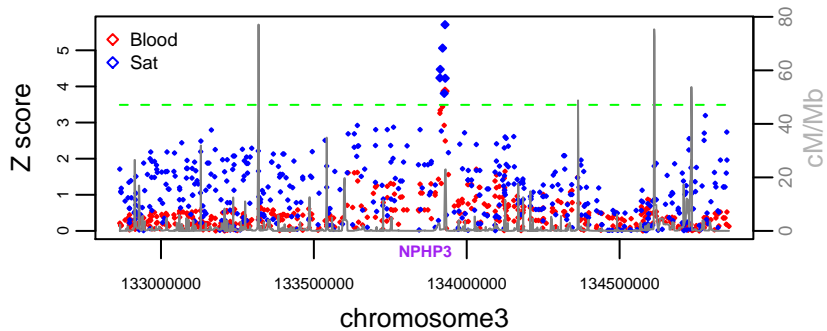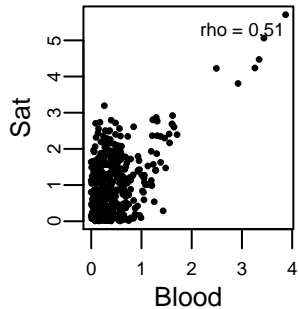

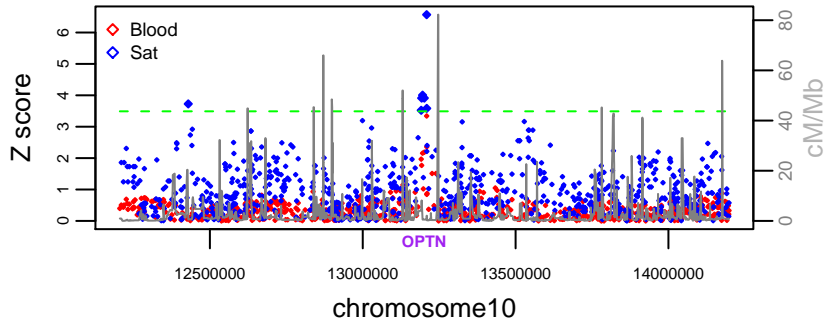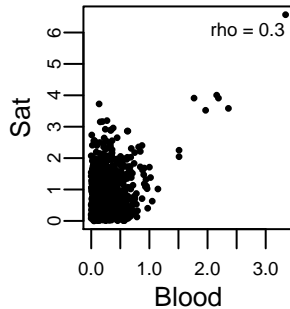

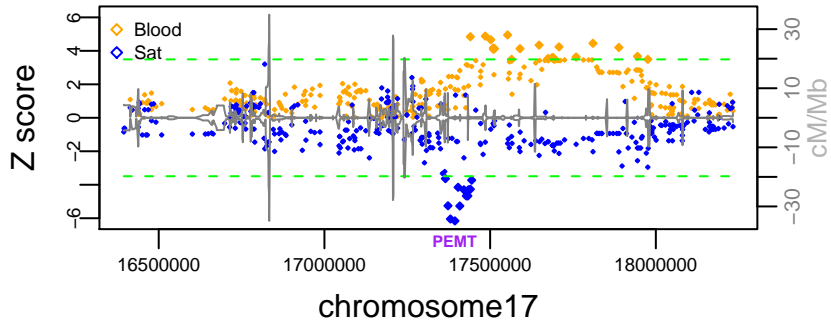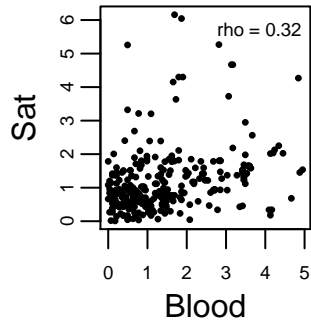

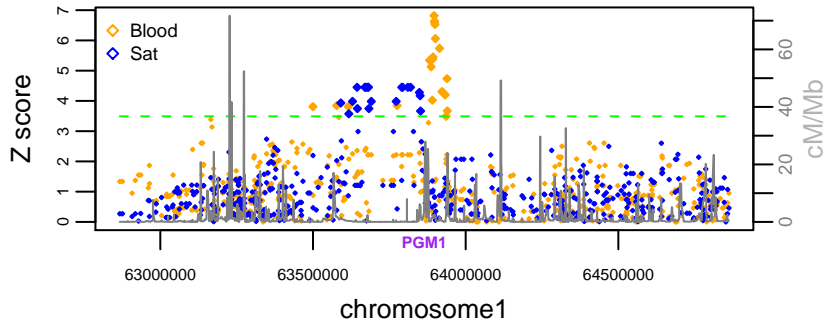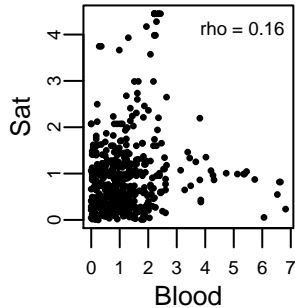

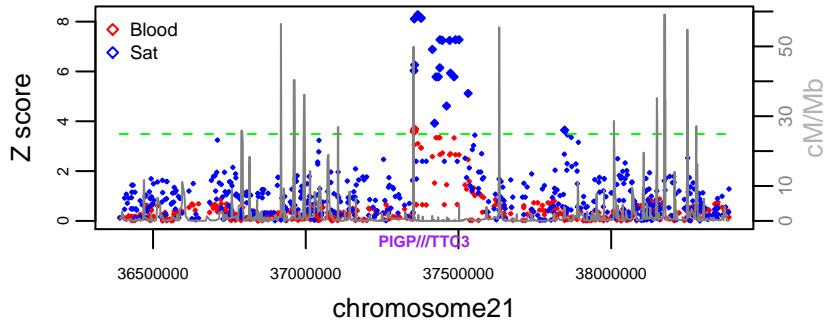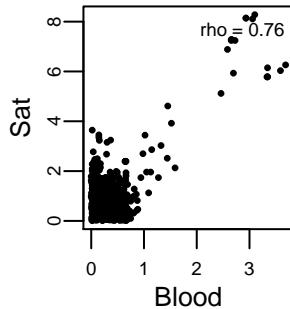

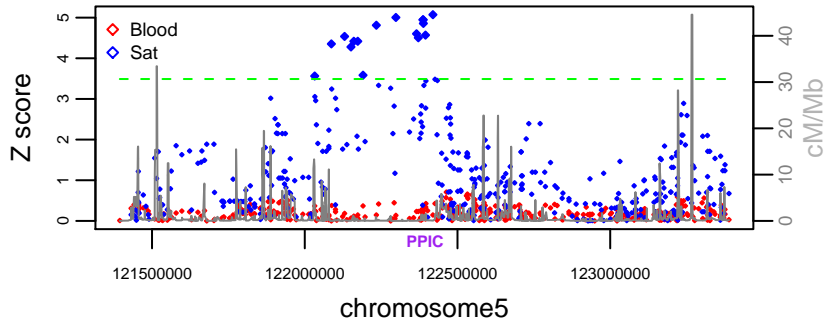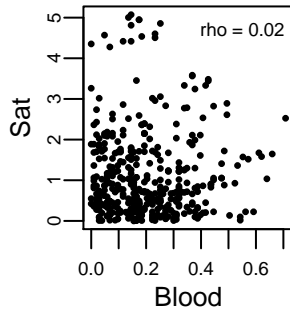

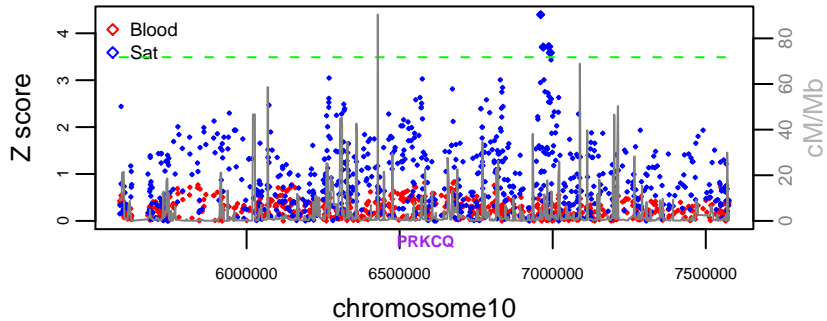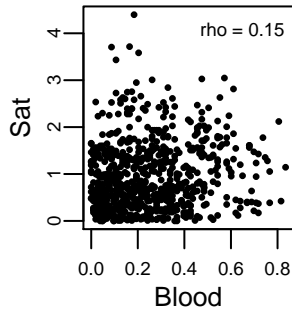

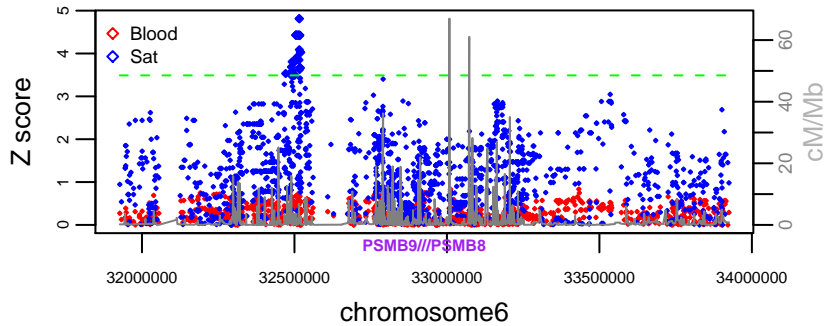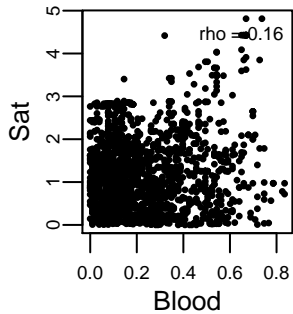

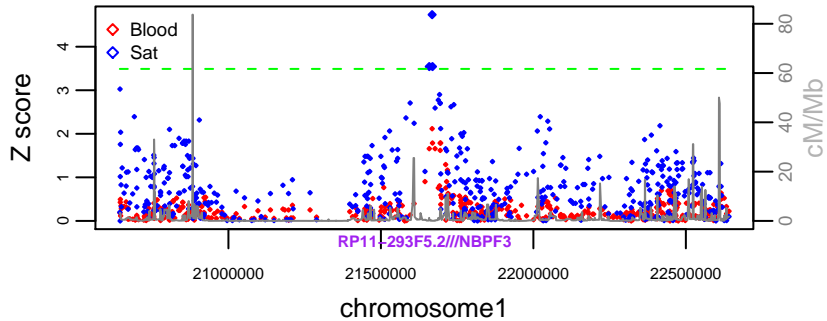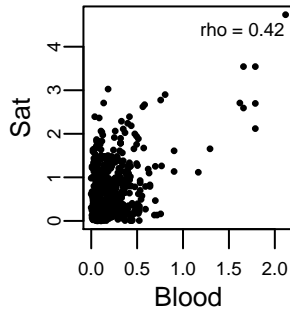

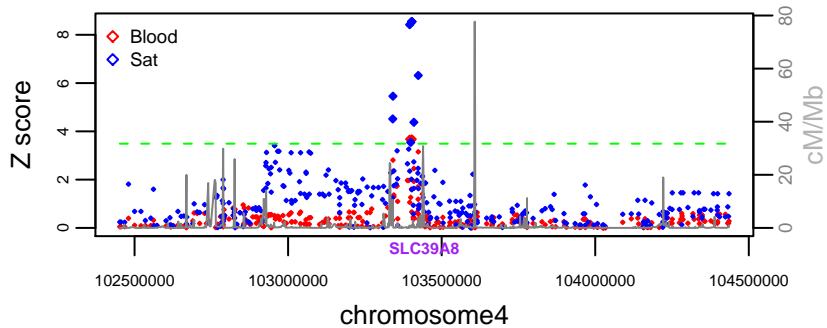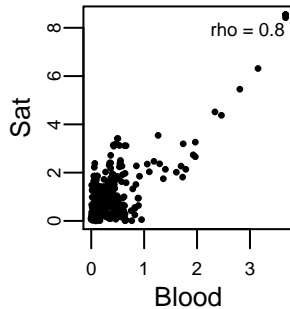

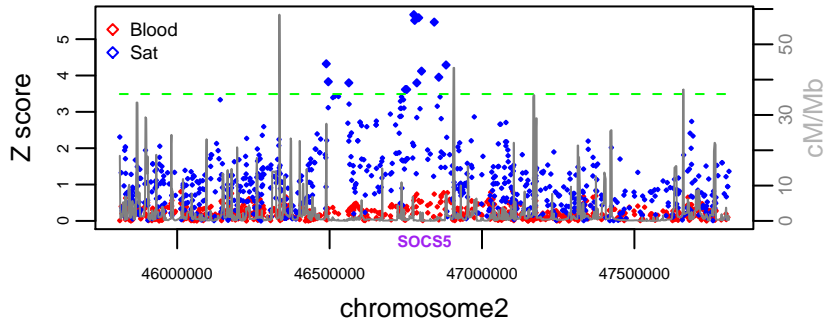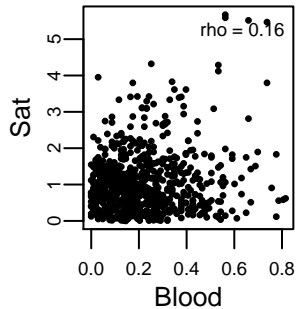

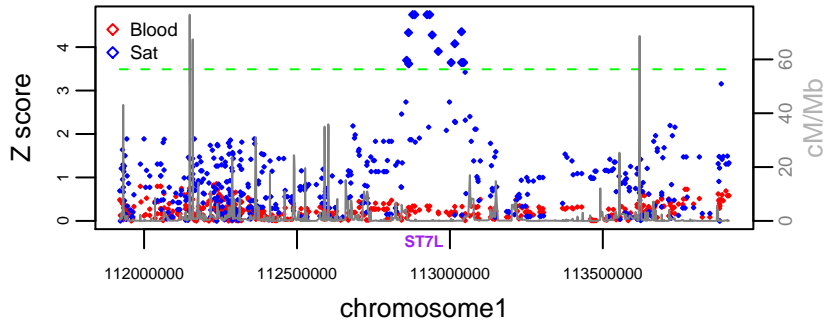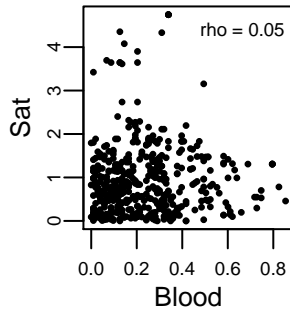

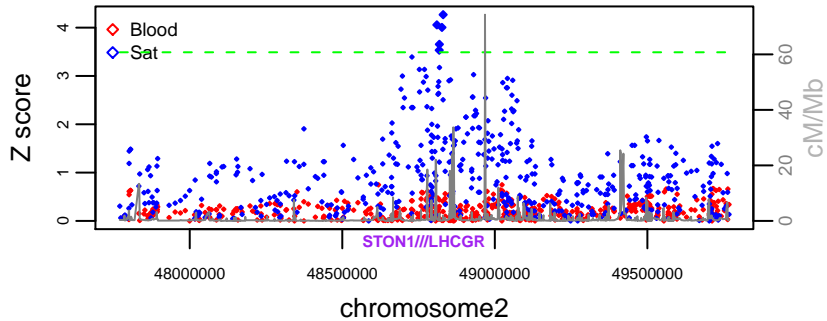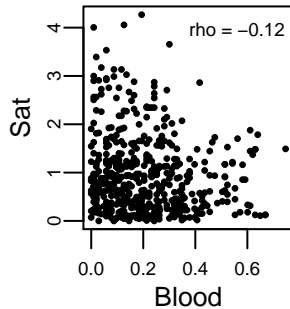

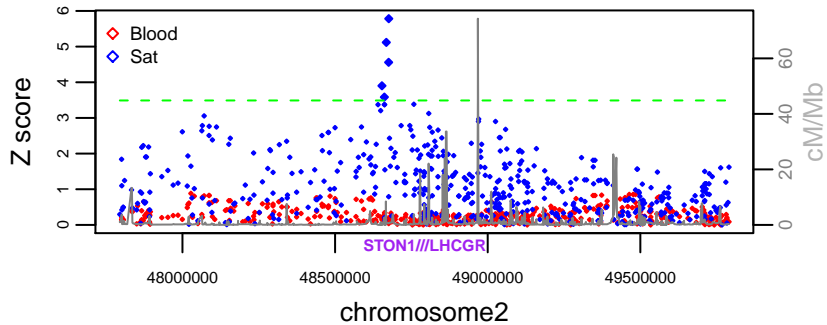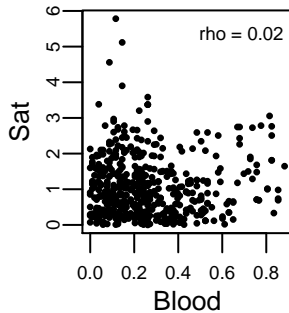

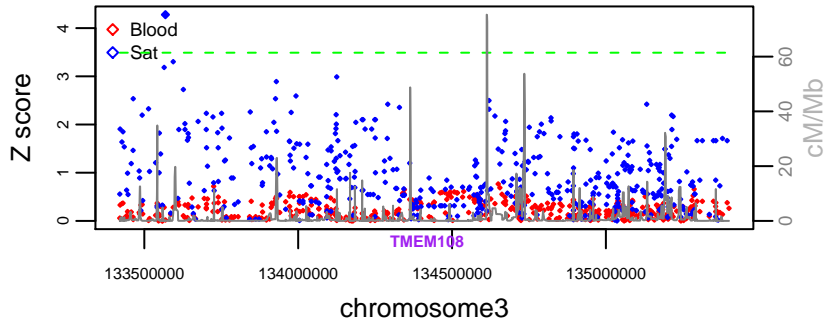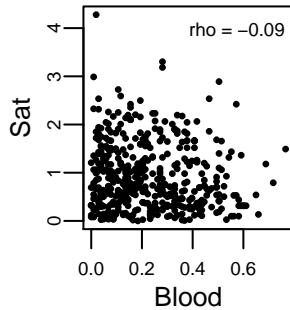

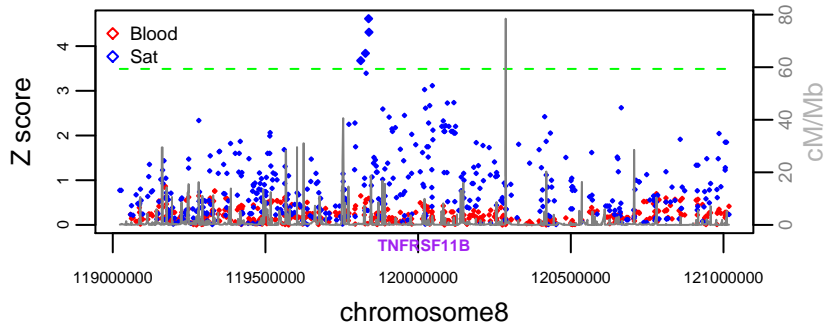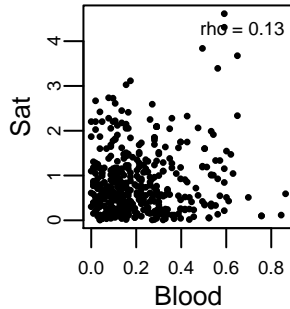

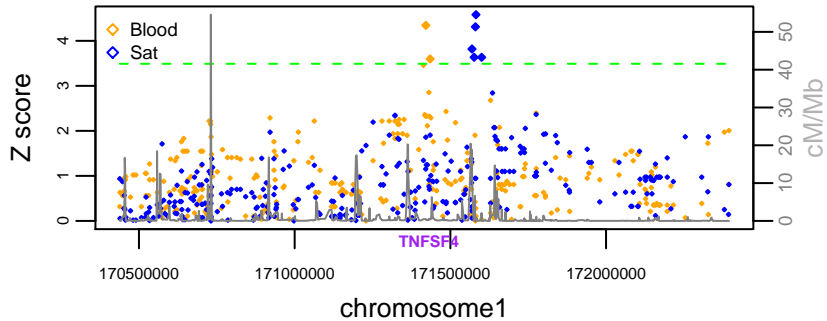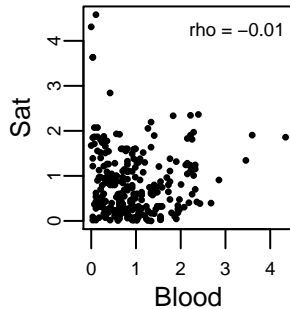

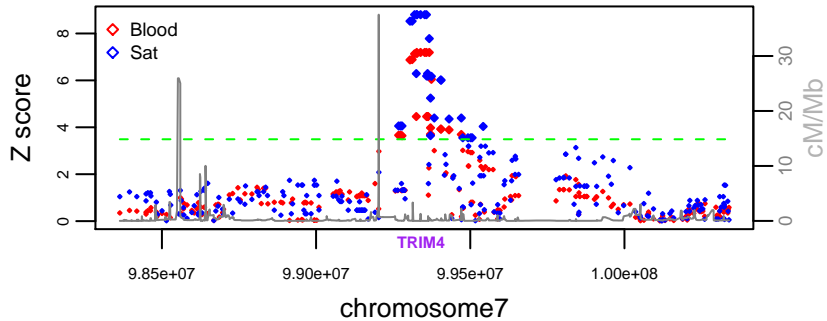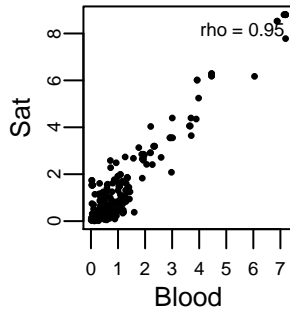

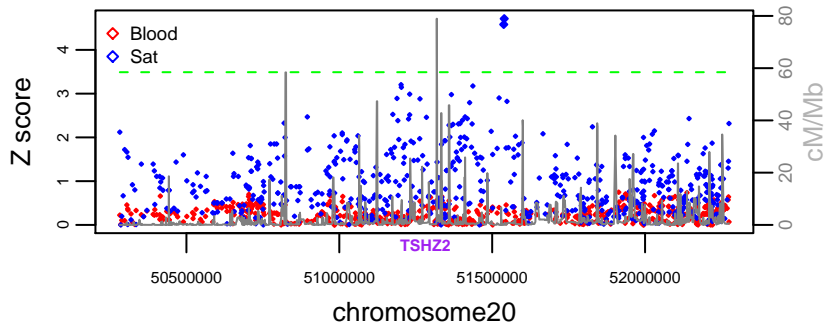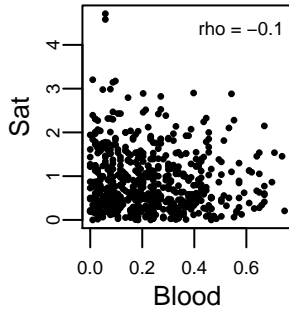

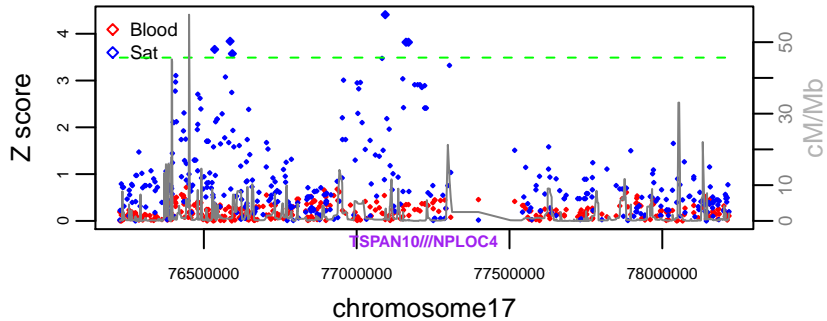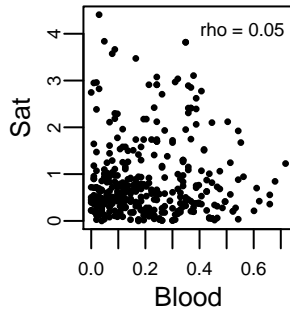

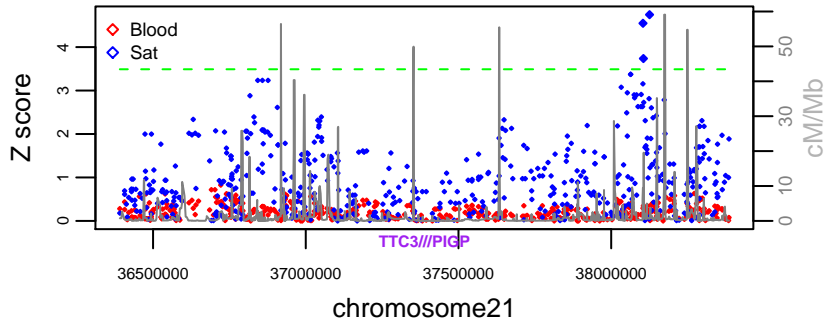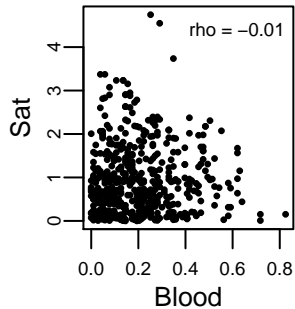

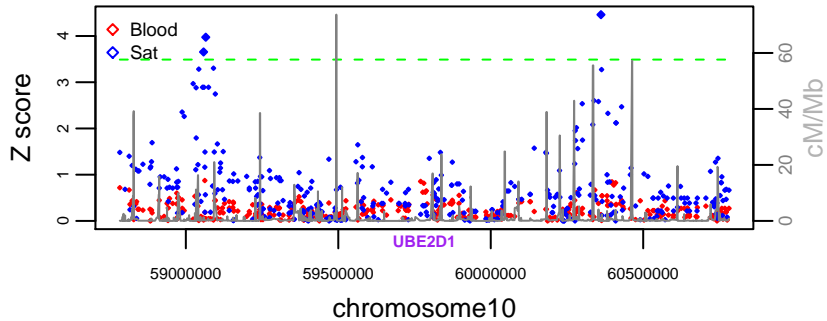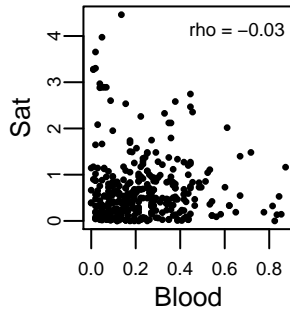

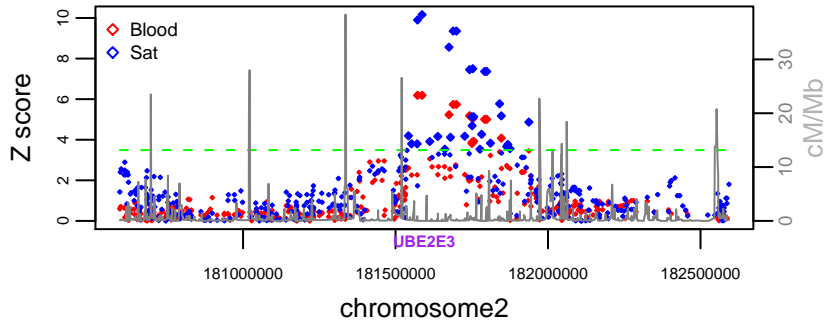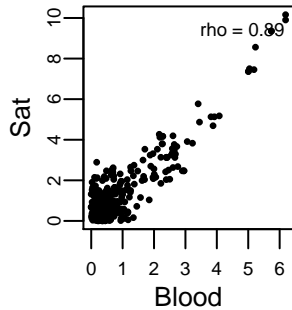

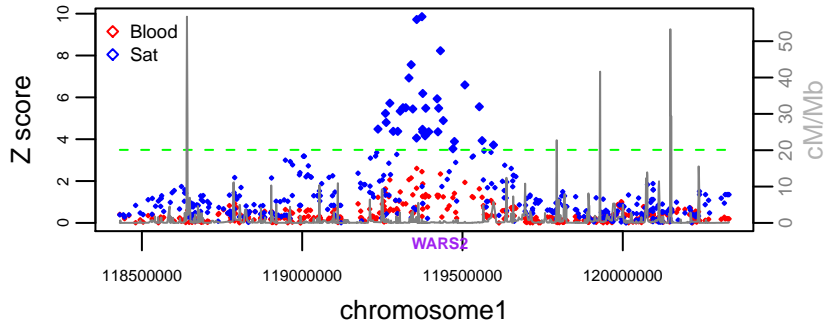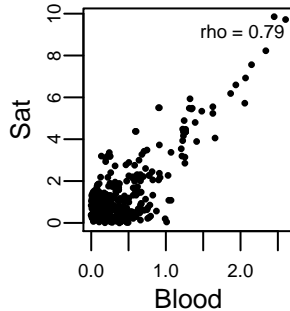

Supplement: Figure S14 — The association profiles of the selected trait-associated genes that show discordant association between blood and SAT. The x-axis is the genome position based on genome build 36.3. The y-axis at the left is the association profiles in terms of the Z-score. The Z-score in blood, represented as the red dots or orange dots. The red dots refer to the Z-scores that have been weighted by the square root of the sample sizes, corresponding to the compared tissue. For the clarity of subtle effect in blood, the weak association in blood was shown as orange dots if the Z-scores have not been weighted by the sample size, i.e., the Z-scores reported in 1,240 subjects. The blue dots represent the Z-scores in SAT. The dashed green line indicates the Z-score 3.49, representing the significance level in blood at FDR 0.05. The right panel shows the correlation of the absolute association Z-scores between two tissues. The rho-value indicates the correlation coefficient of the Pearson correlation. (PDF) [file pgen.1002431.s014.pdf]

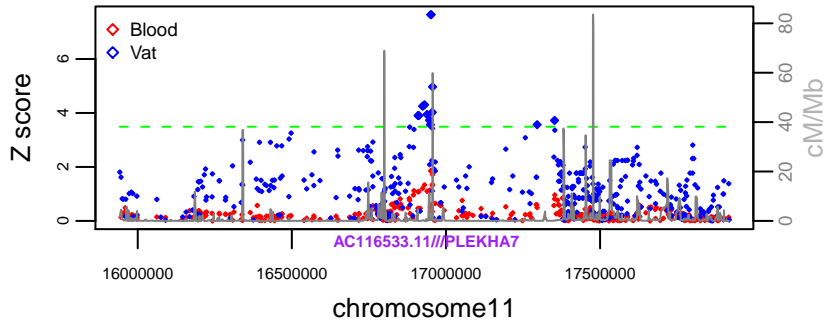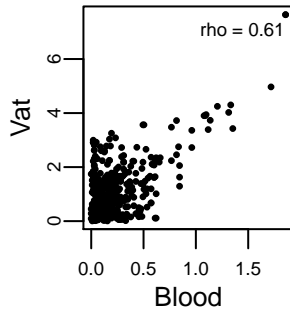

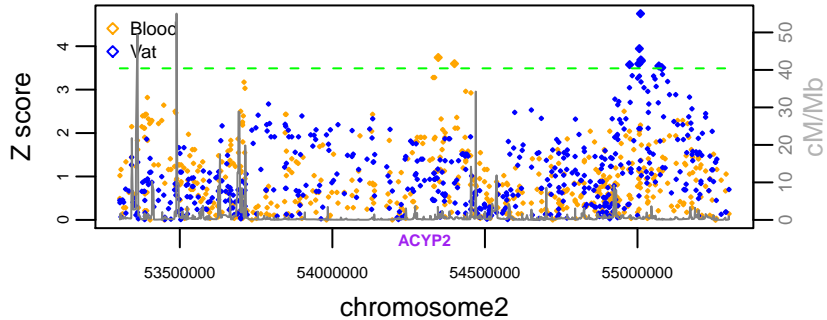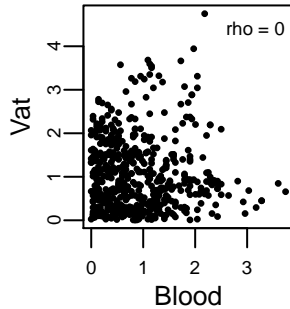

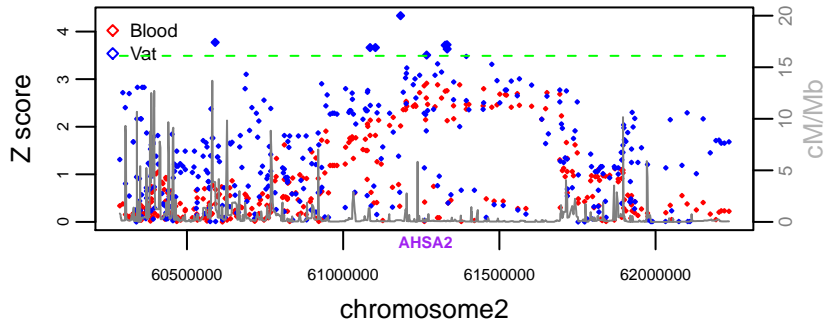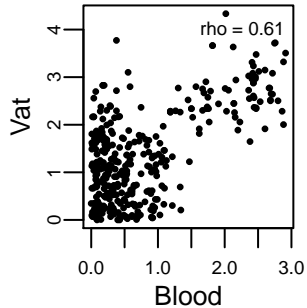

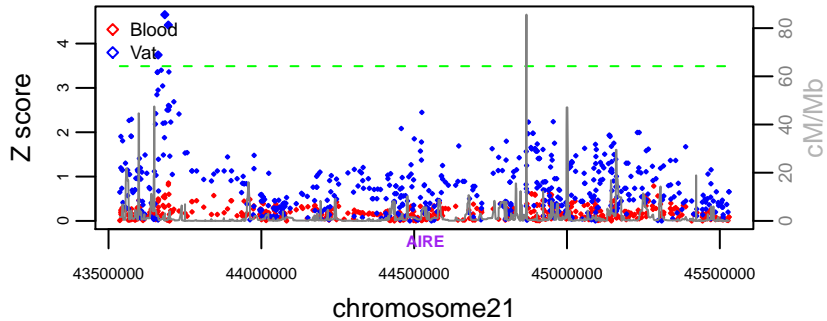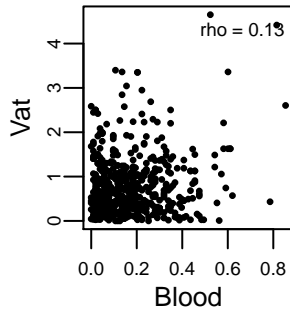

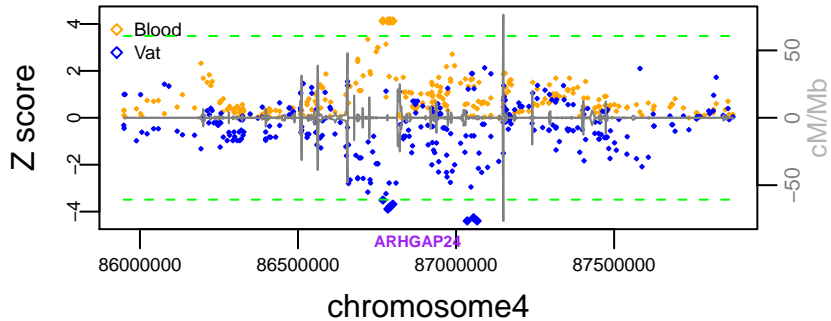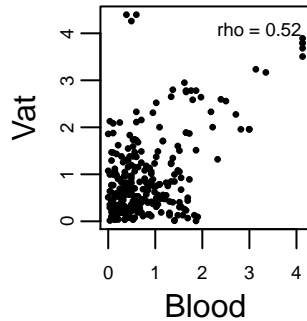

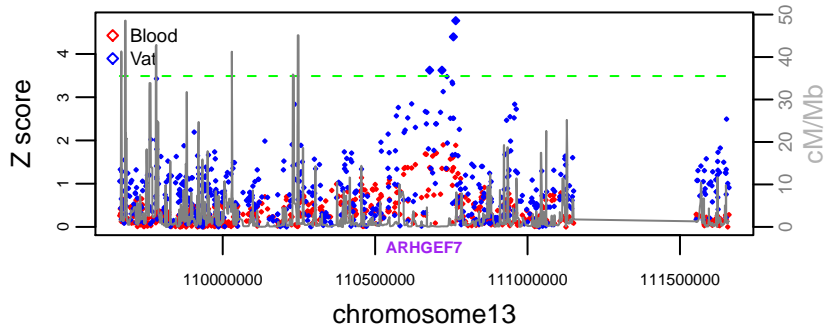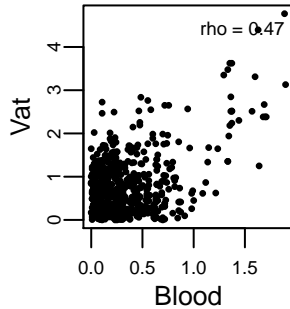

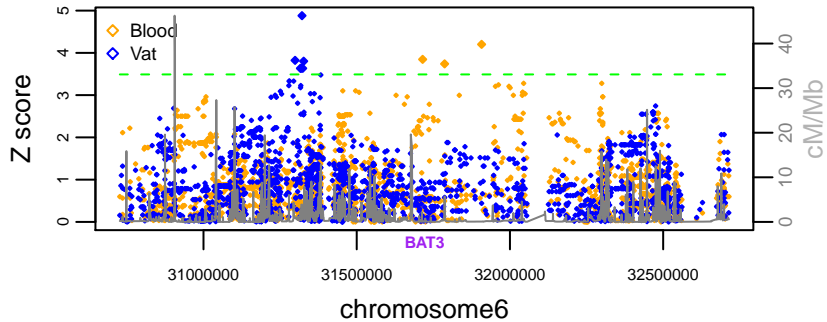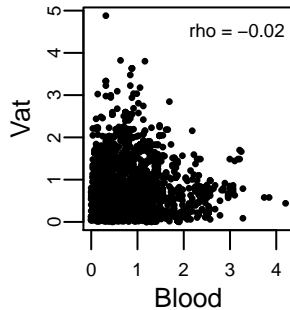

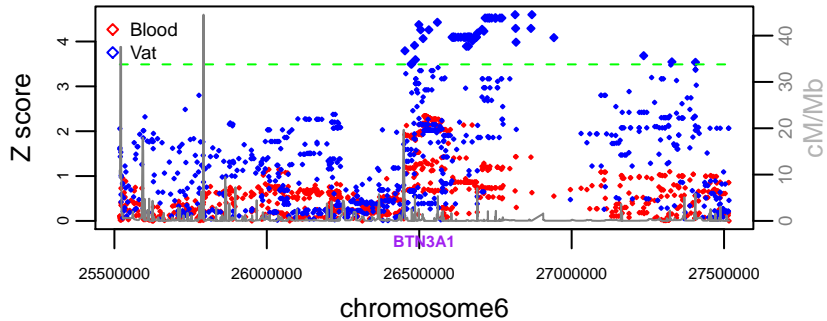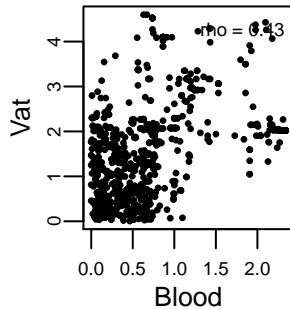

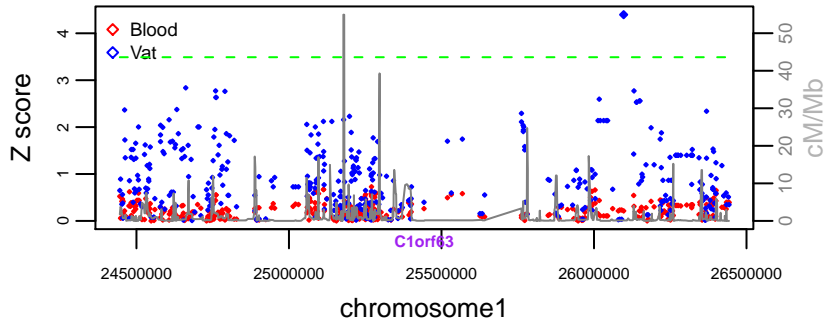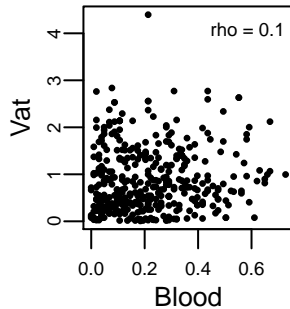

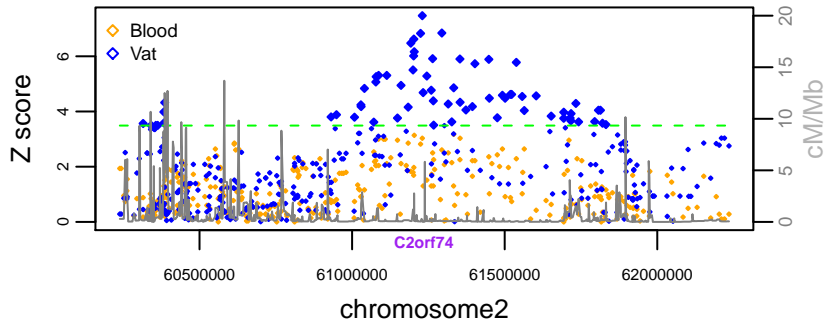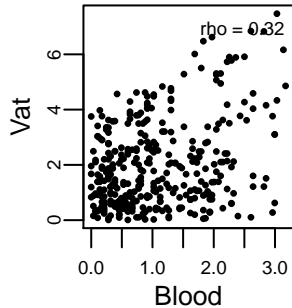

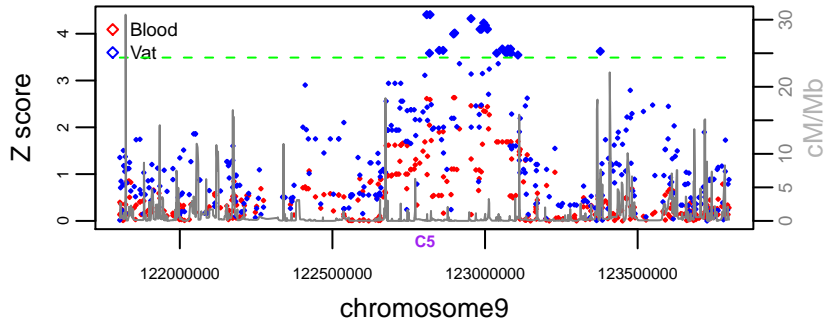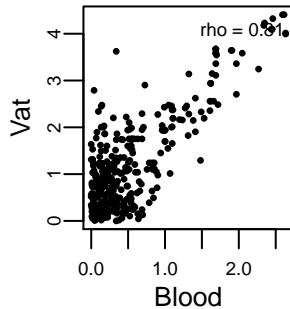

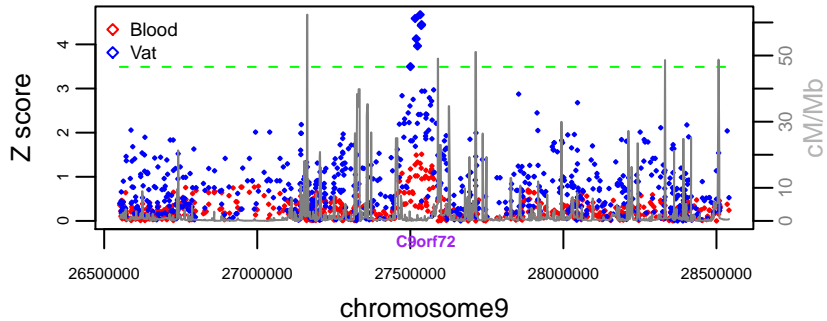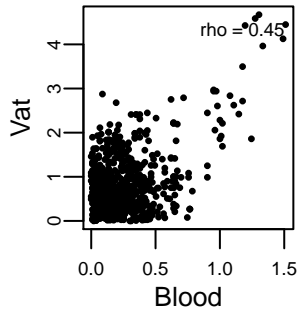

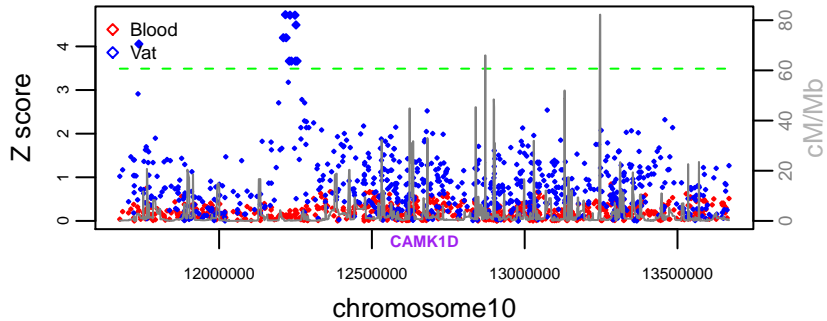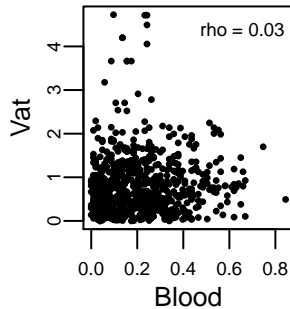

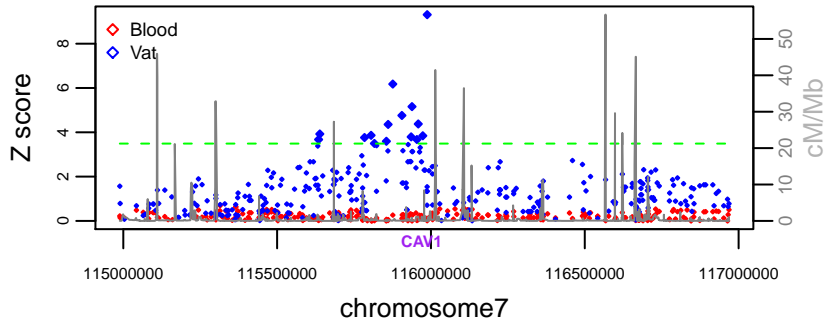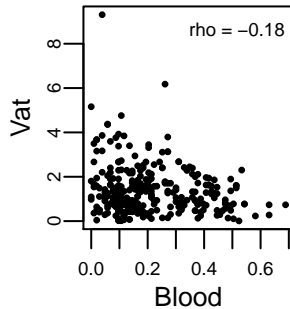

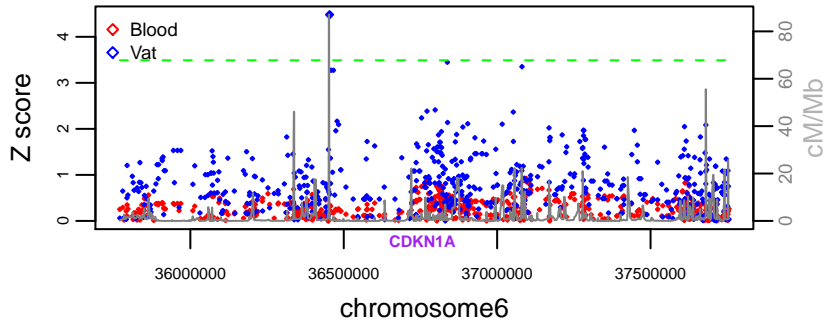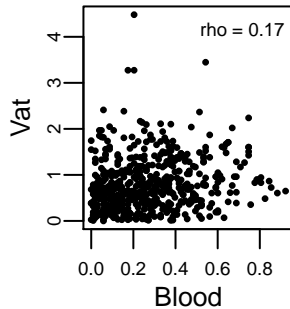

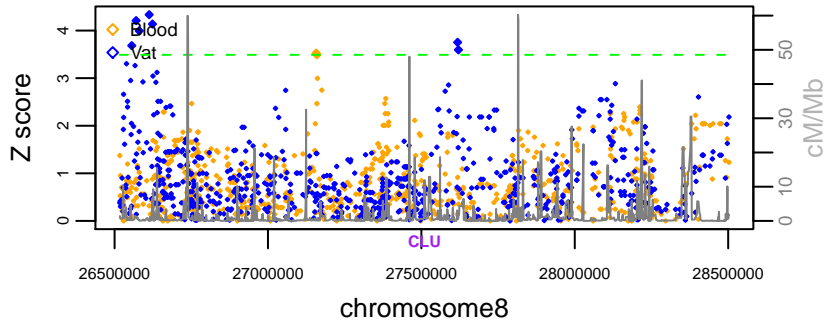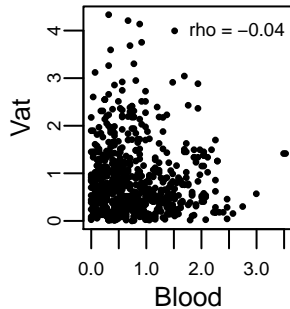

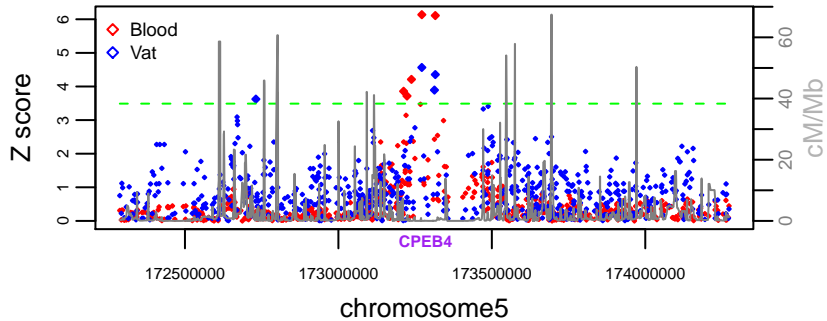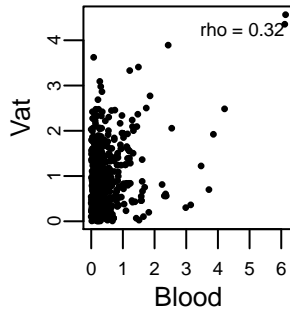

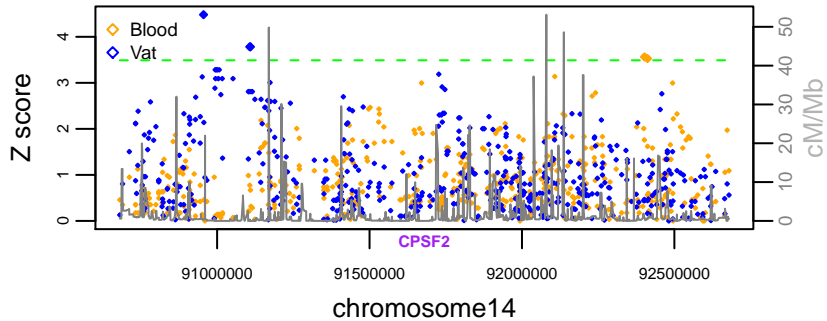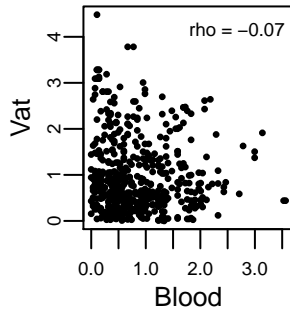

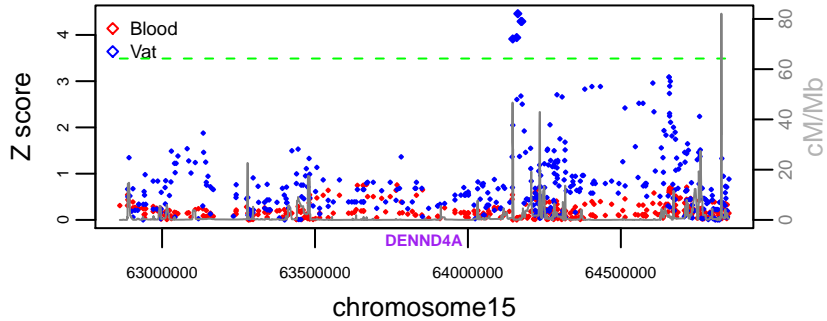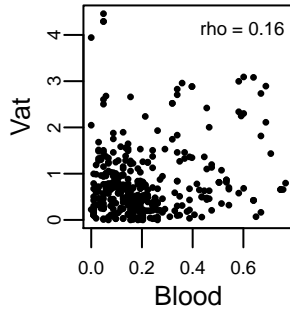

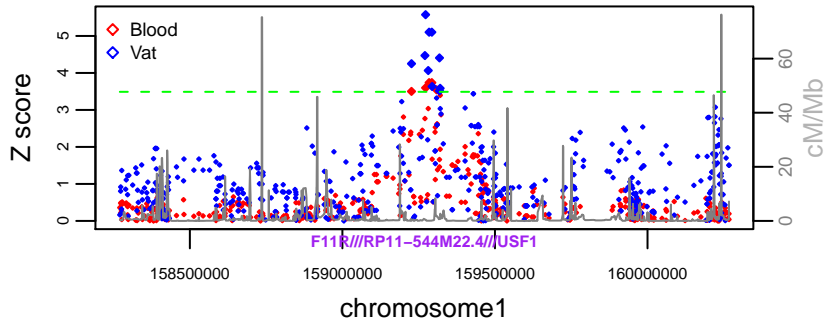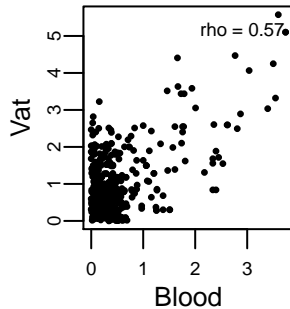

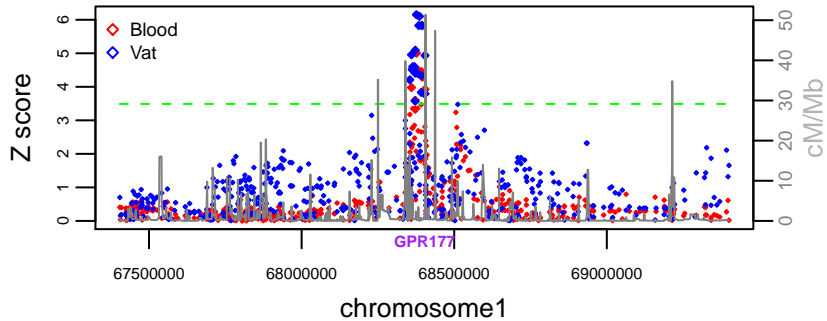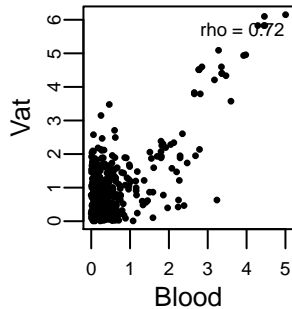

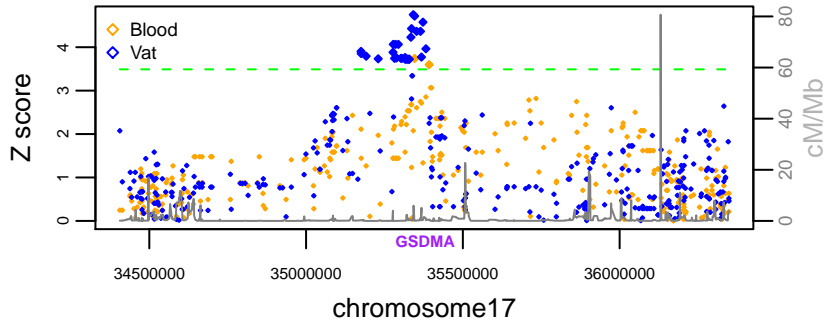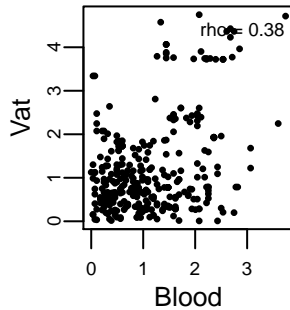

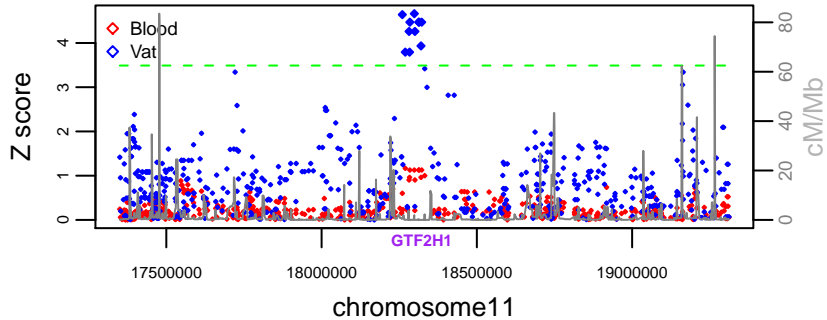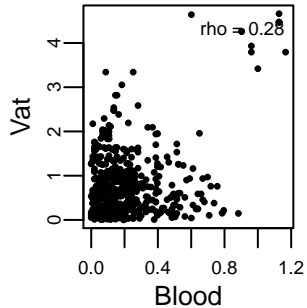

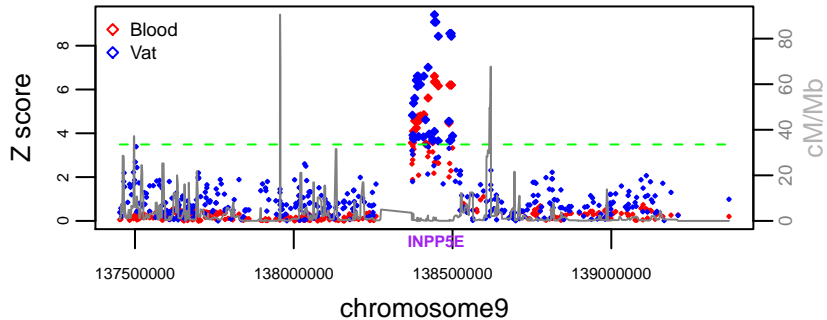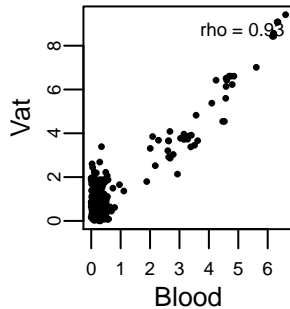

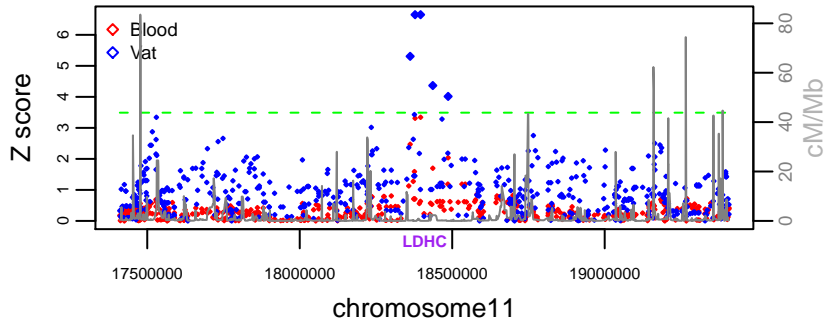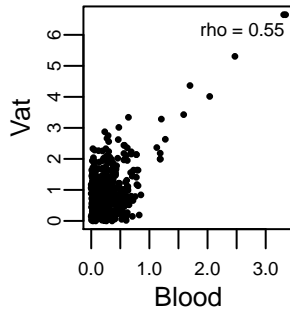

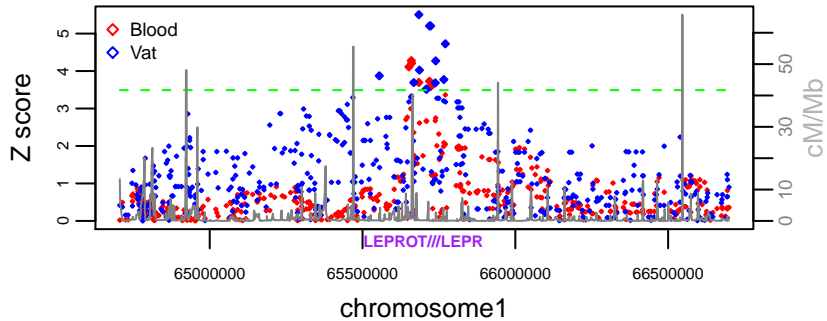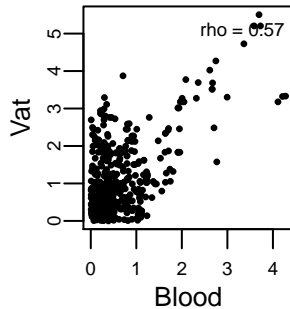

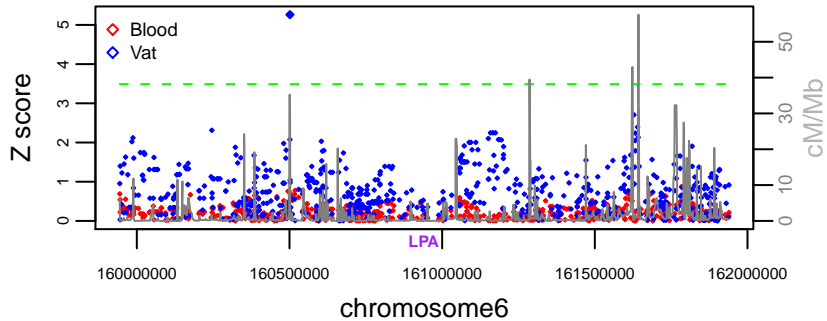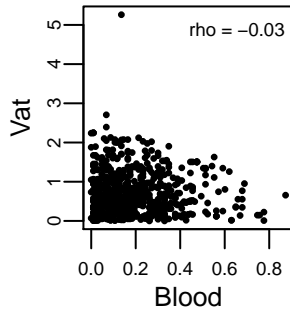

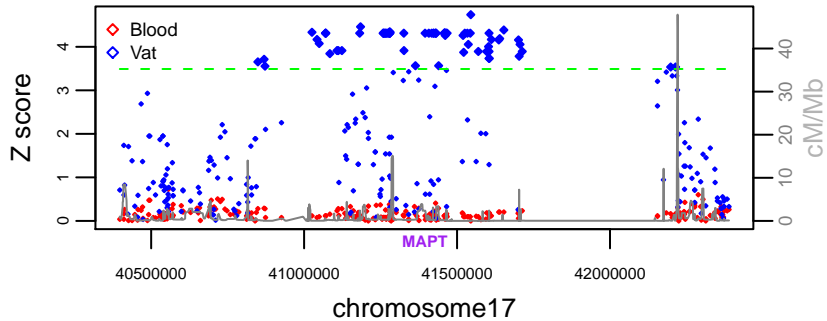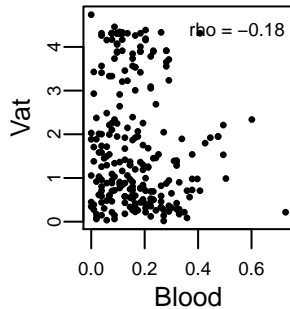

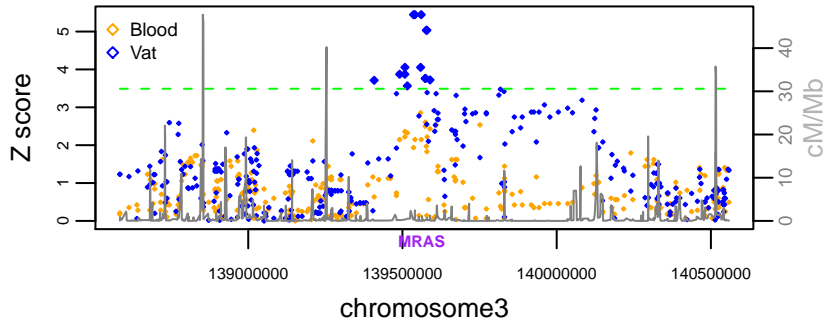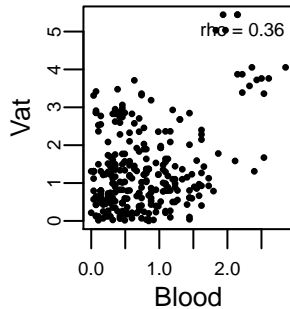

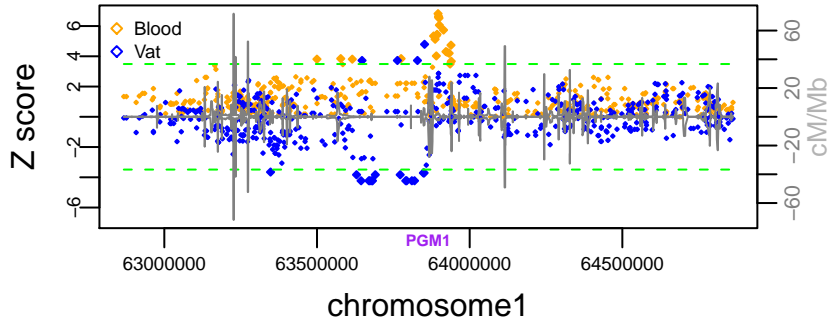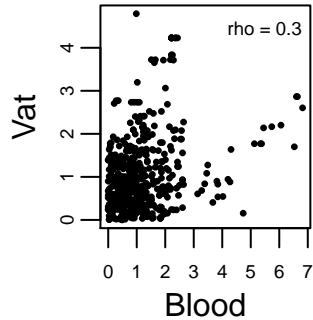

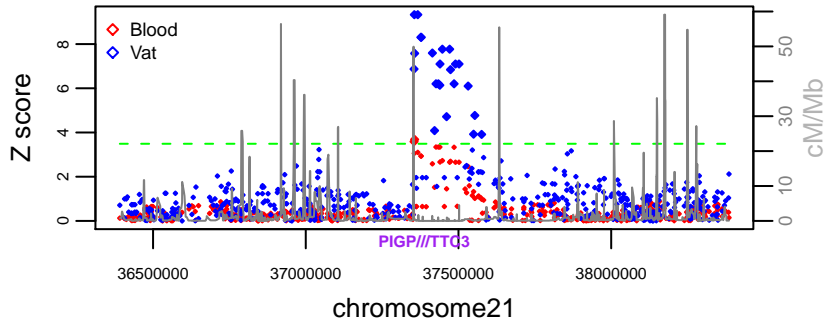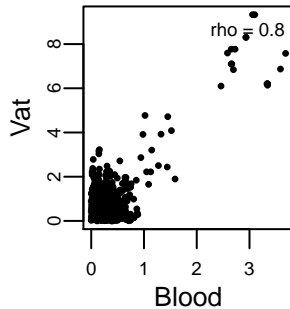

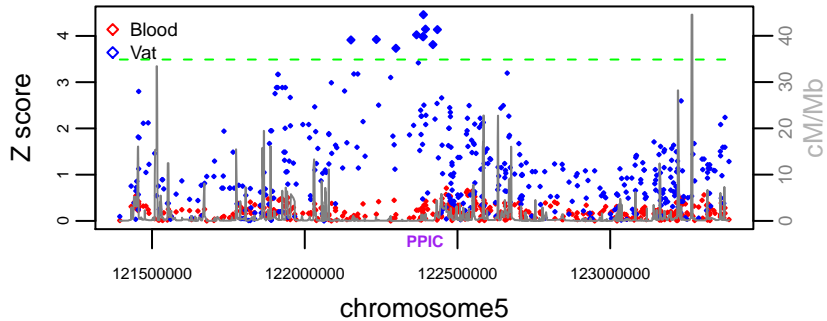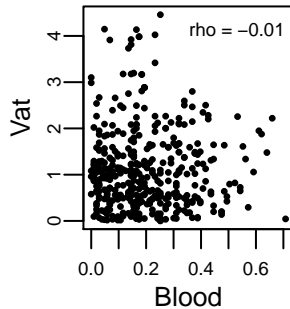

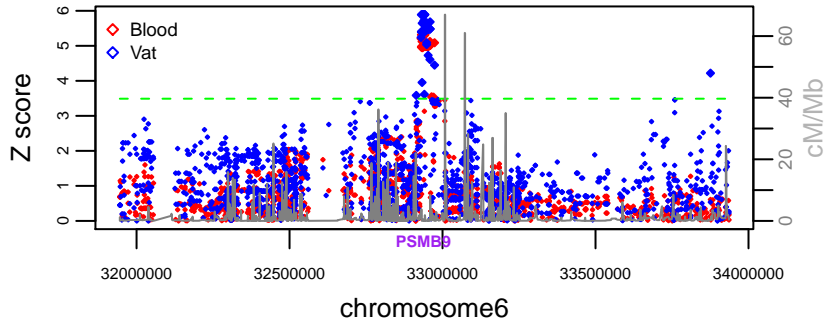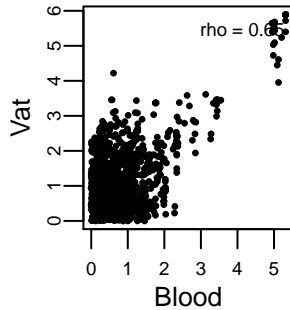

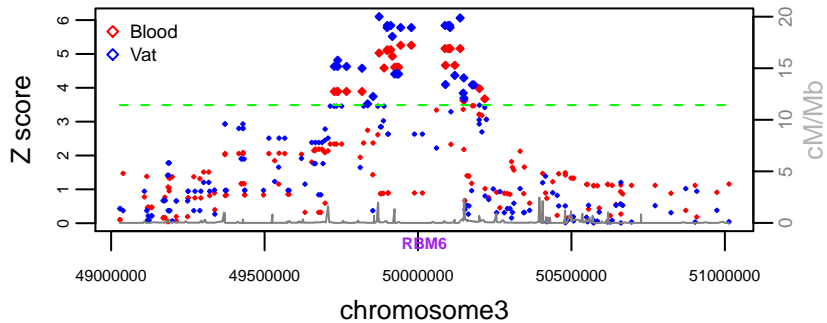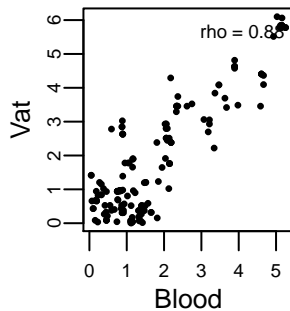

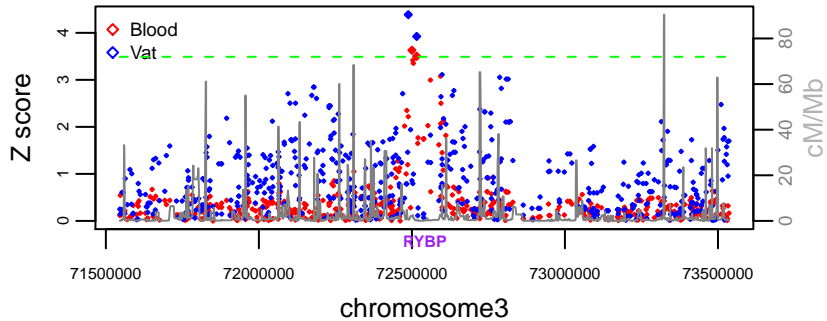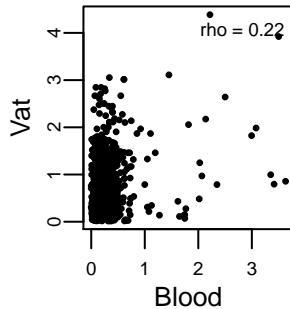

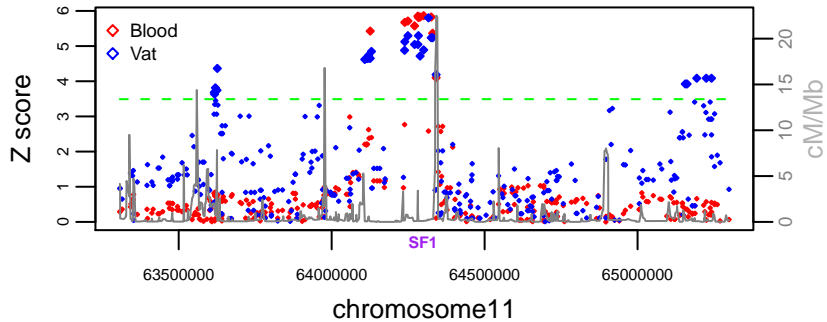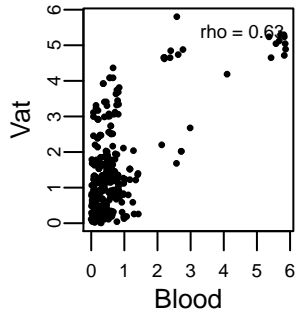

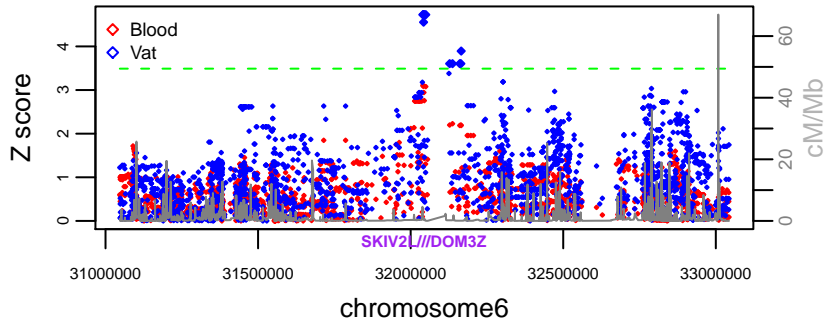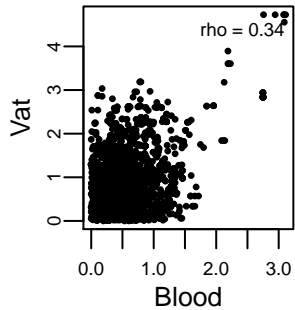

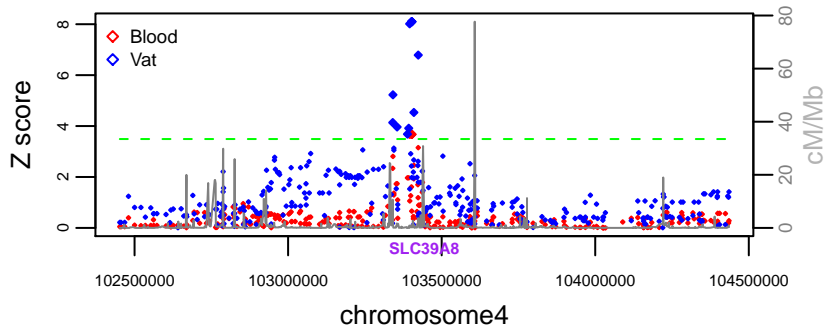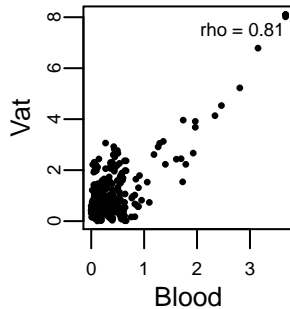

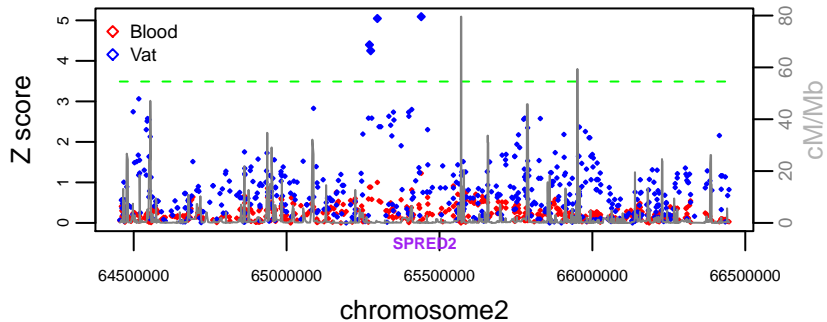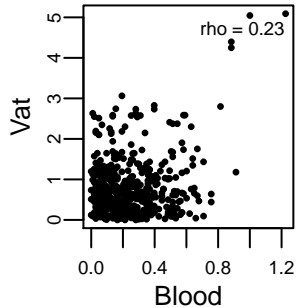

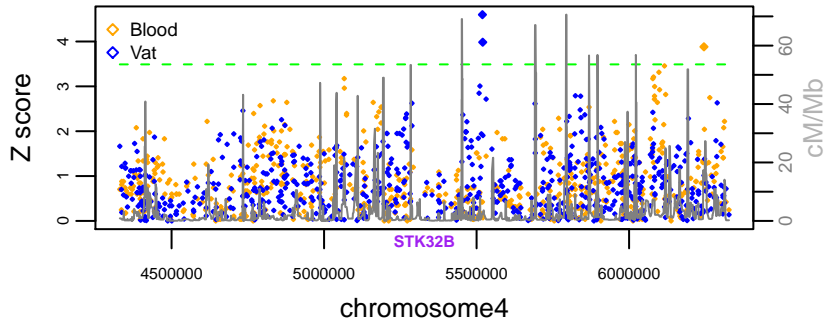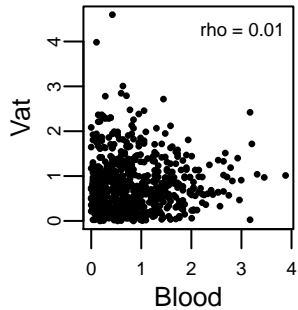

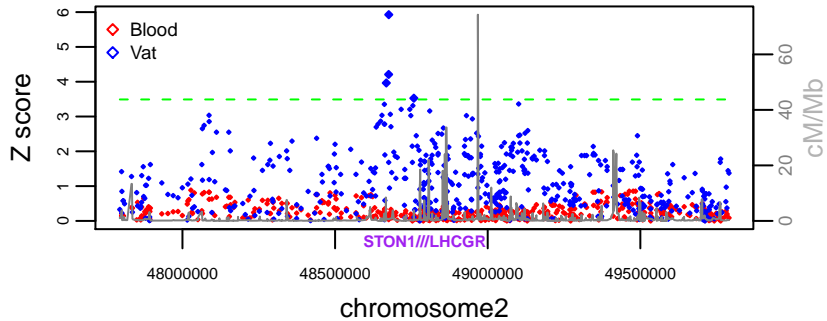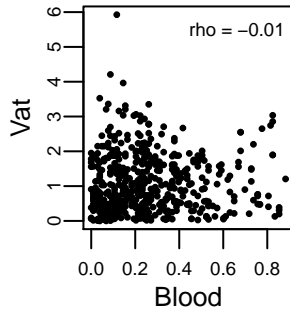

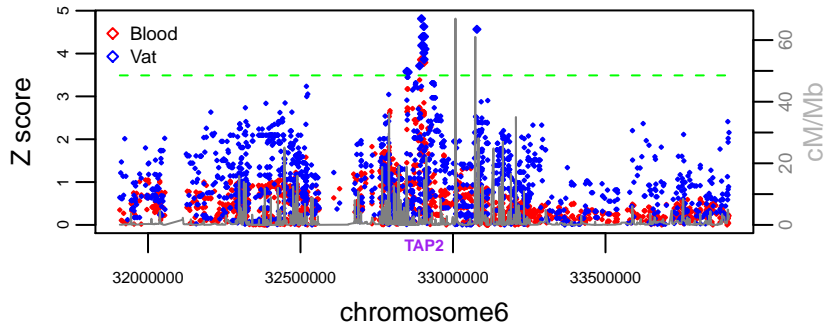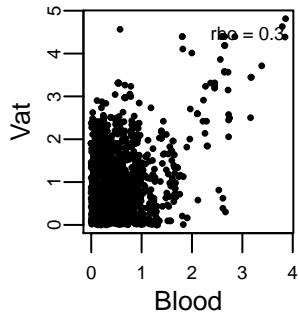

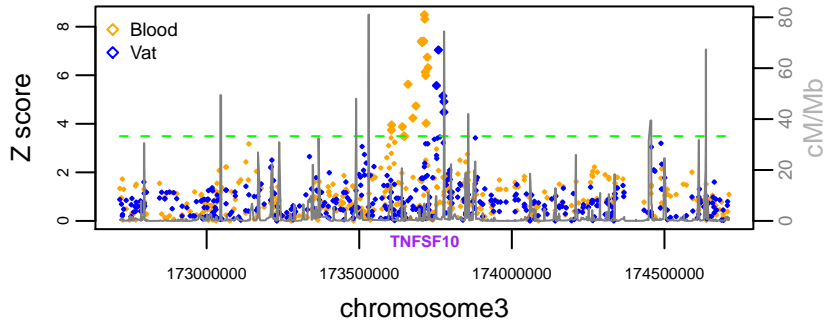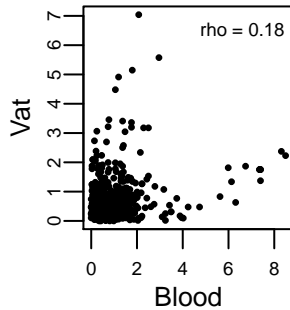

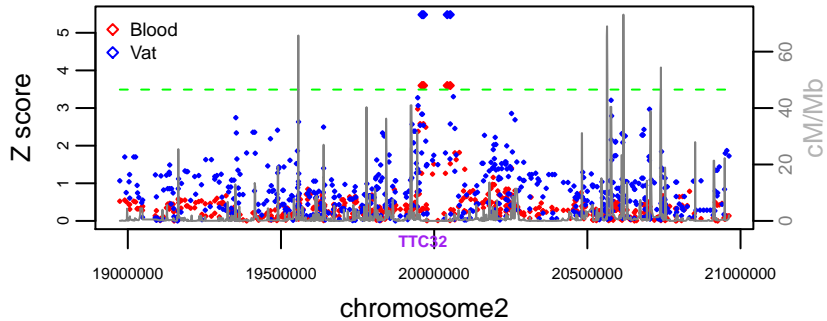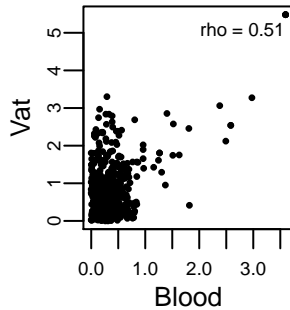

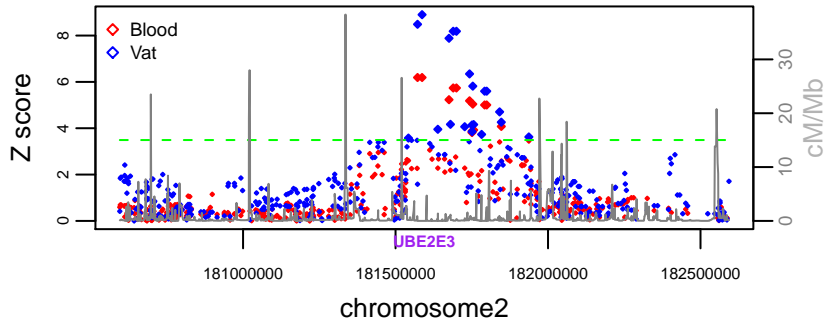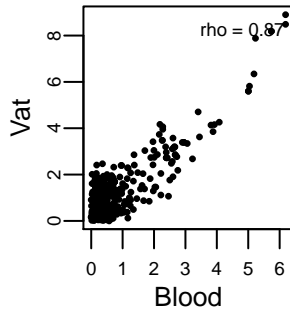

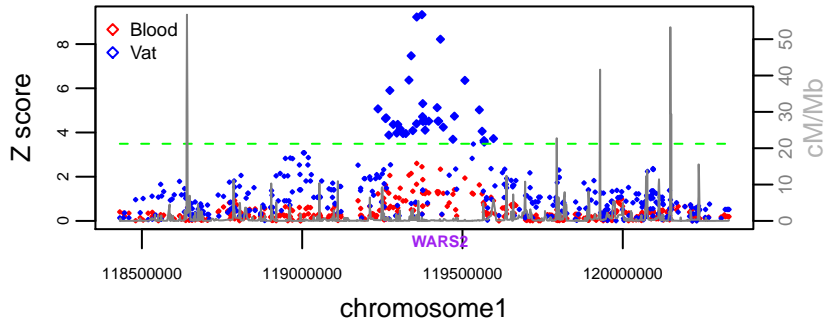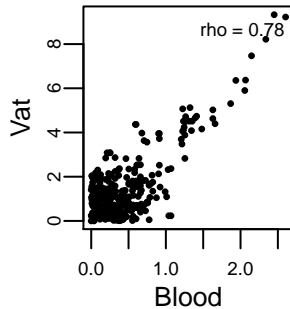

Supplement: Figure S15 — The association profiles of the selected trait-associated genes that show discordant association between blood and VAT. The x-axis is the genome position based on genome build 36.3. The y-axis at the left is the association profiles in terms of the Z-score. The Z-score in blood, represented as the red dots or orange dots. The red dots refer to the Z-scores that have been weighted by the square root of the sample sizes, corresponding to the compared tissue. For the clarity of subtle effect in blood, the weak association in blood was shown as orange dots if the Z-scores have not been weighted by the sample size, i.e., the Z-scores reported in 1,240 subjects. The blue dots represent the Z-scores in VAT. The dashed green line indicates the Z-score 3.49, representing the significance level in blood at FDR 0.05. The right panel shows the correlation of the absolute association Z-scores between two tissues. The rho-value indicates the correlation coefficient of the Pearson correlation. (PDF) [file pgen.1002431.s015.pdf]

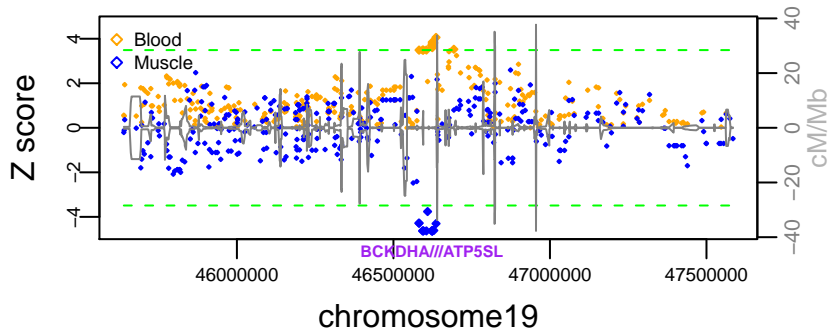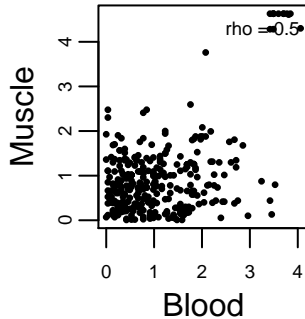

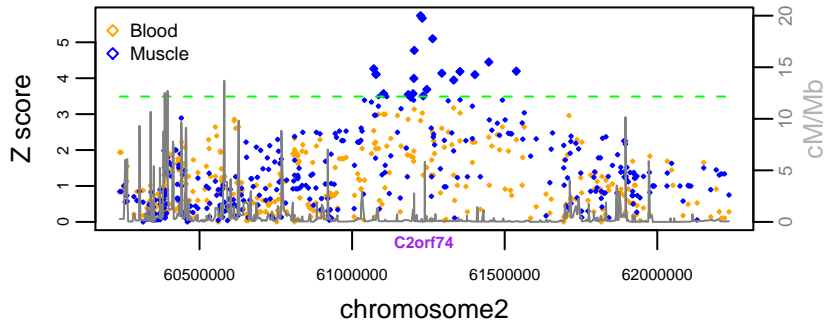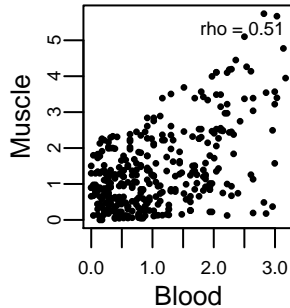

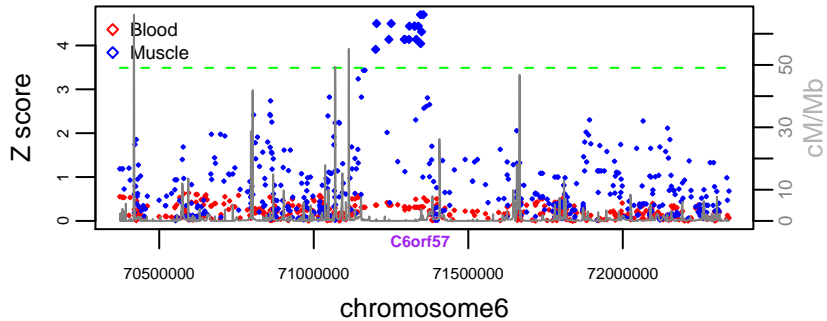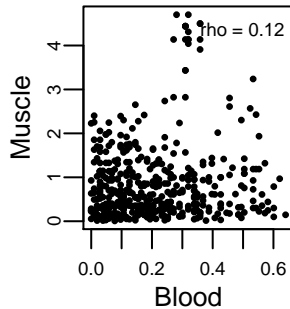

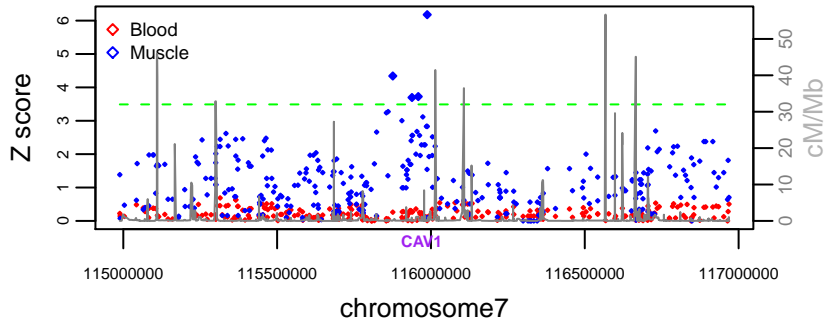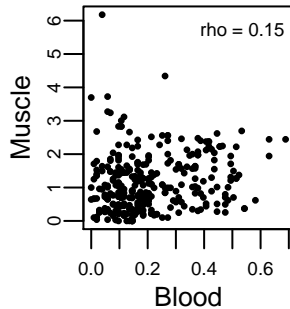

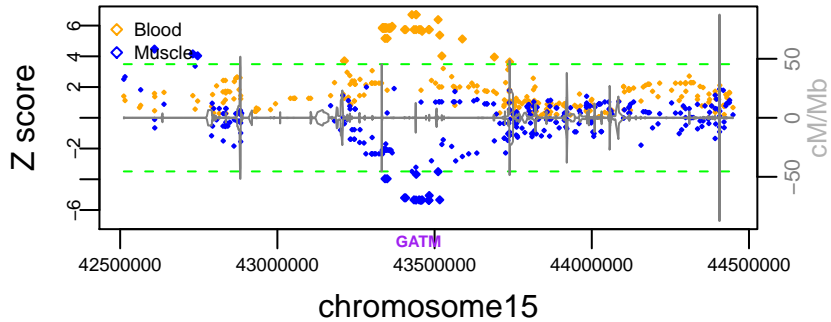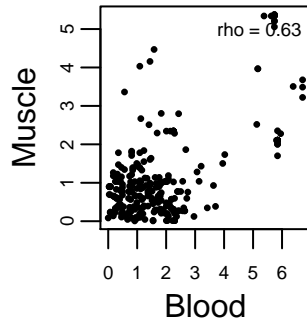

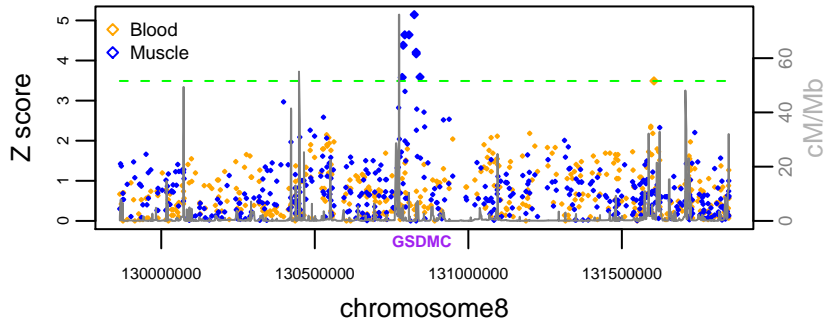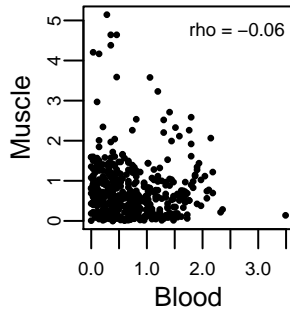

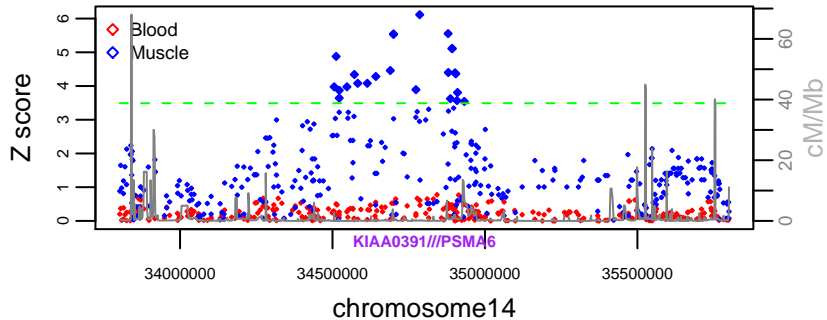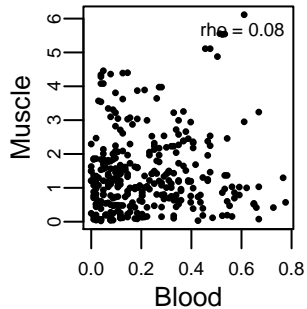

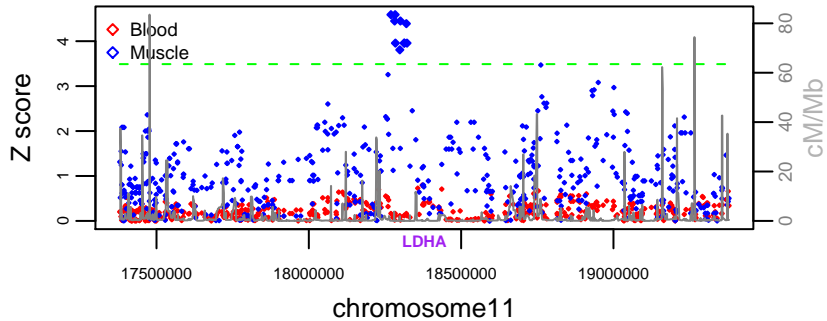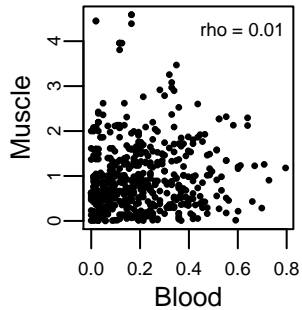

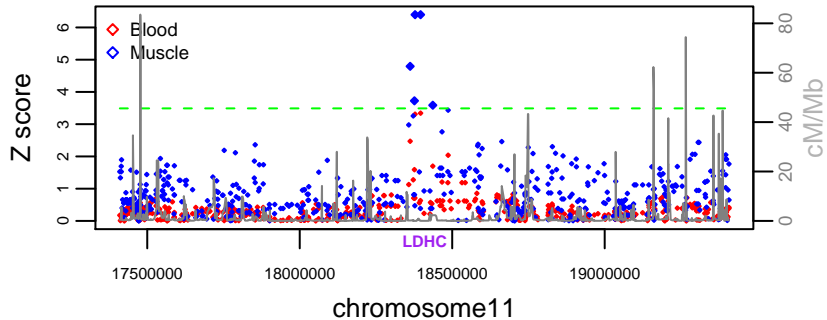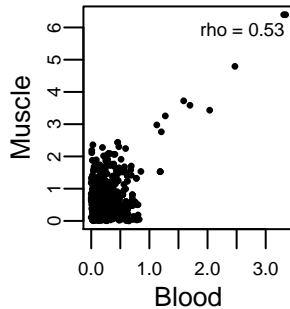

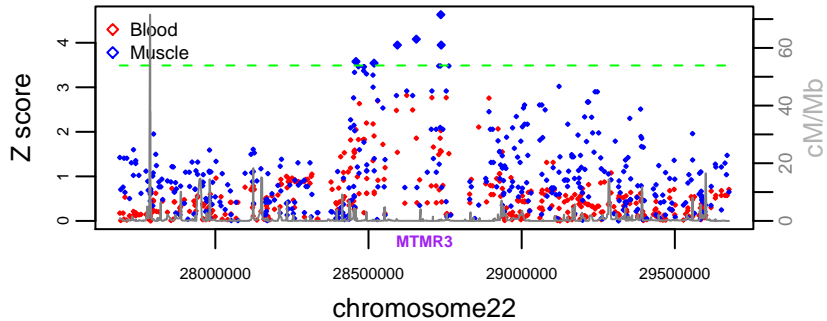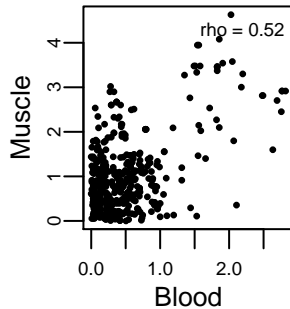

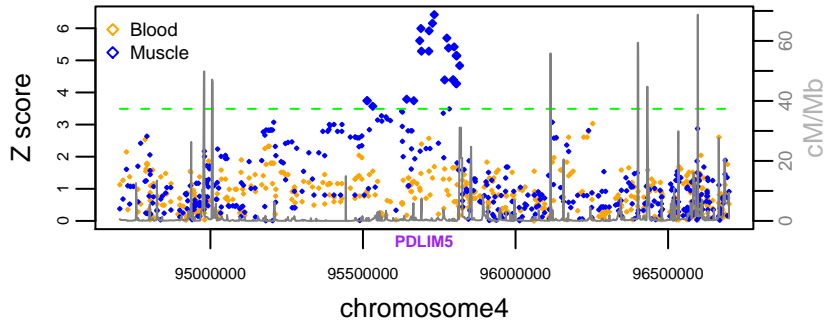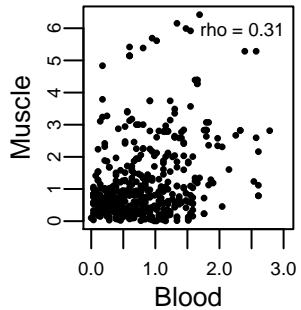

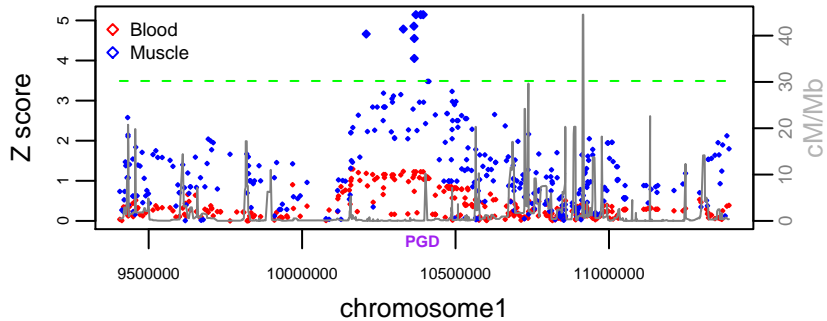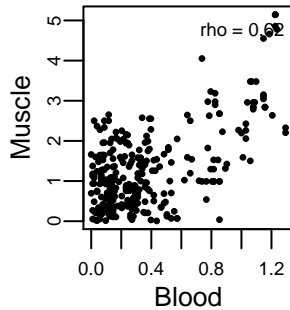

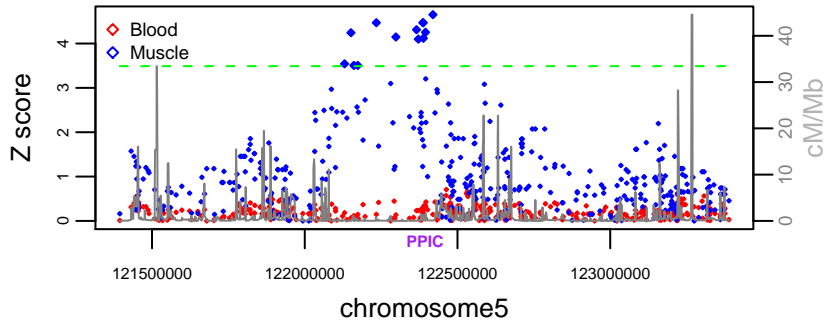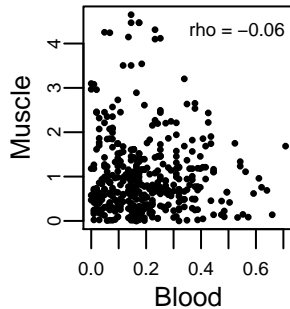

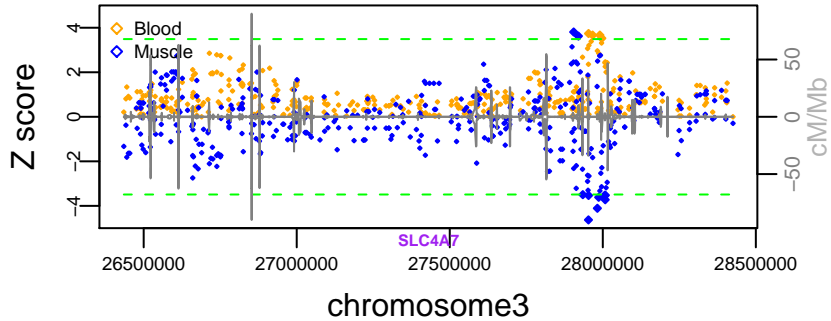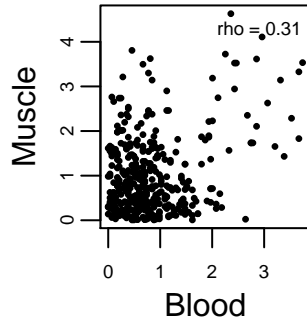

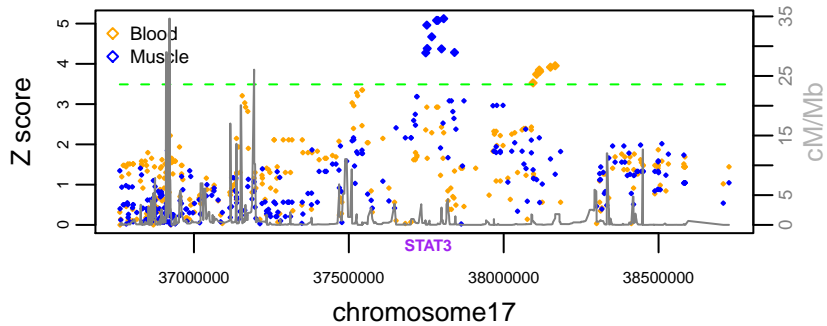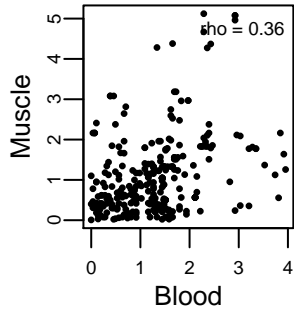

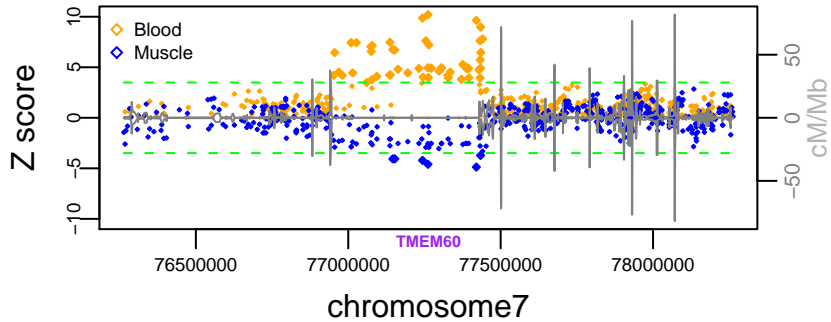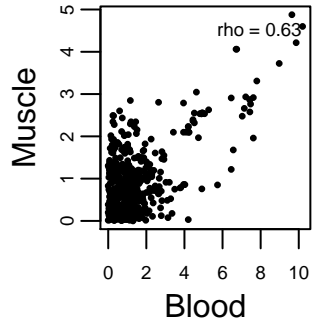

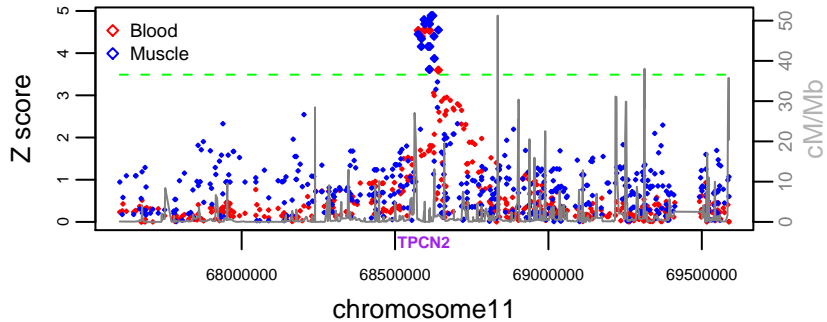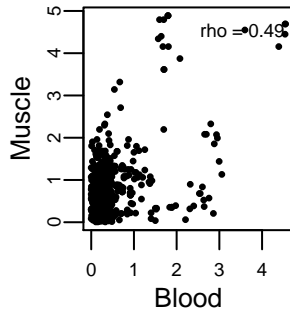

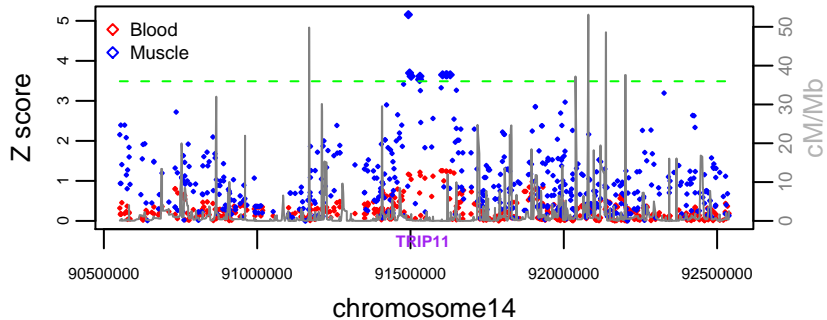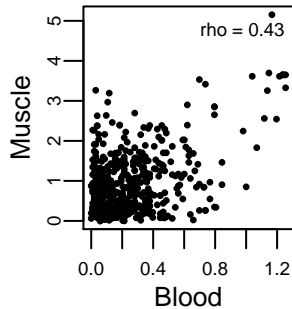

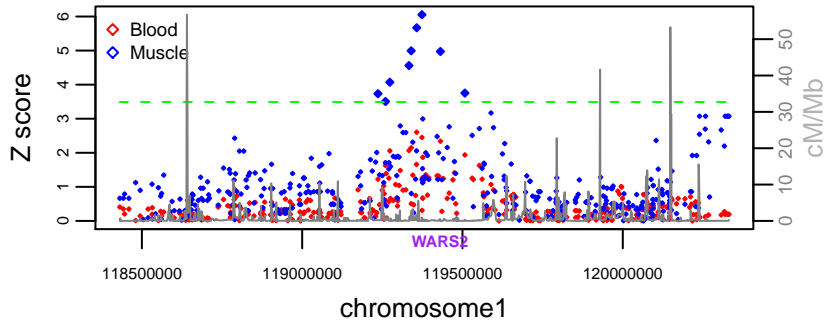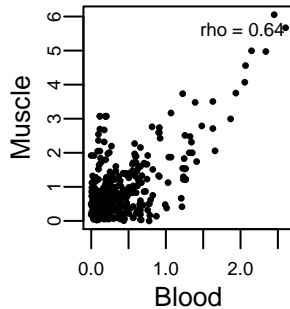

Supplement: Figure S16 — The association profiles of the selected trait-associated genes that show discordant association between blood and muscle. The x-axis is the genome position based on genome build 36.3. The y-axis at the left is the association profiles in terms of the Z-score. The Z-score in blood, represented as the red dots or orange dots. The red dots refer to the Z-scores that have been weighted by the square root of the sample sizes, corresponding to the compared tissue. For the clarity of subtle effect in blood, the weak association in blood was shown as orange dots if the Z-scores have not been weighted by the sample size, i.e., the Z-scores reported in 1,240 subjects. The blue dots represent the Z-scores in muscle. The dashed green line indicates the Z-score 3.49, representing the significance level in blood at FDR 0.05. The right panel shows the correlation of the absolute association Z-scores between two tissues. The rho-value indicates the correlation coefficient of the Pearson correlation. (PDF) [file pgen.1002431.s016.pdf]

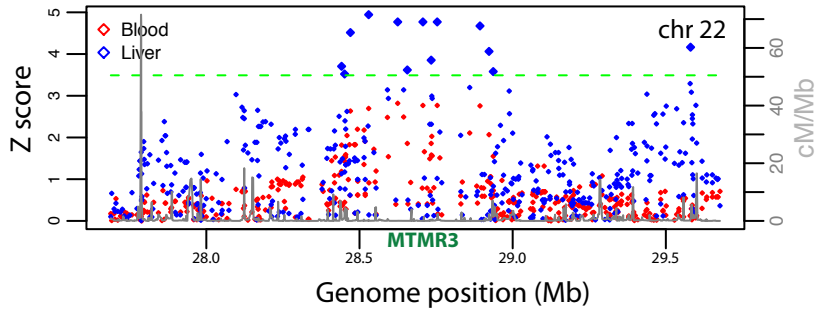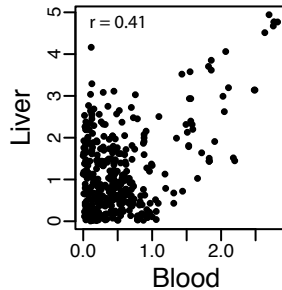

Supplement: Figure S17 — Association profiles of MTMR3 in blood and liver. The x-axis is the genome position based on genome build 36.3 (in Mb). The y-axis at the left indicates the association Z-score. The Z-scores in blood, represented as the red dots, have been weighted by the square root of the sample size, corresponding to the compared tissue. The blue dots represent the Z-scores in SAT. The dashed green line indicates the Z-scores 3.49, representing the significance level in blood at FDR 0.05. The right panel shows the correlation of the absolute association Z-scores between two tissues. The r-value indicates the correlation coefficient of the Pearson correlation. (PDF) [file pgen.1002431.s017.pdf]
