# Supplementary material for: Unraveling the Regulatory Mechanisms Underlying Tissue-Dependent Genetic Variation of Gene Expression
Source: PLoS Genet. 2012 Jan 19;8(1):e1002431. doi: 10.1371/journal.pgen.1002431 (PMC3261927; doi:10.1371/journal.pgen.1002431)
Supplement: Figure S11 — The direction of allelic effect of rs5751777 on DDT expression. The correlation between the genotype of rs5751777 and the expression intensity of DDT gene (residual variance after 50 PCs removed) in five tissues. Each dot represents one subject, red for females and blue for males. The X-axis represents the genotypes and the Y-axis represents the expression rank of the probes. (PDF) [file pgen.1002431.s011.pdf]

SNP rs5751777 - Chr: 22 (22597047)
Probe 2360593 - DDTL///DDT, Chr: 22 (22640290 - 22649703)
P-Value 1.54E-106 **P-Value Abs.** 1.74E-150

Blood

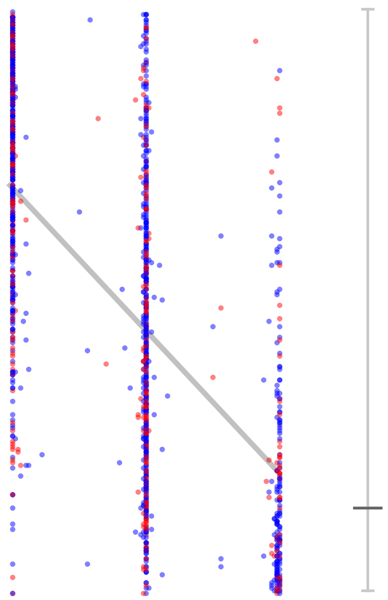

TT (444) TC (583) CC (213)

Corr: -0.594 **R2:** 0.353
Z-Score: -23.206 **P-Value:** 3.98E-119

SAT

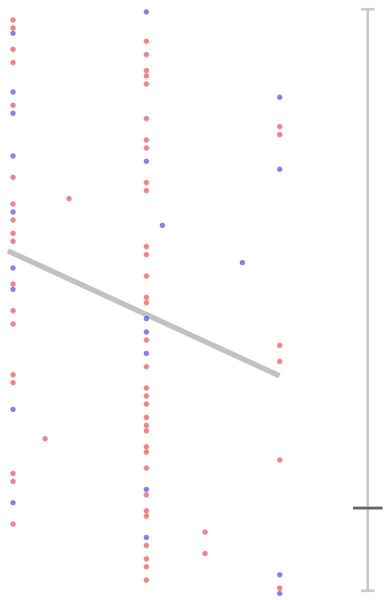

TT (29) TC (43) CC (11)

Corr: -0.236 **R2:** 0.056
Z-Score: -2.148 **P-Value:** 0.032

VAT

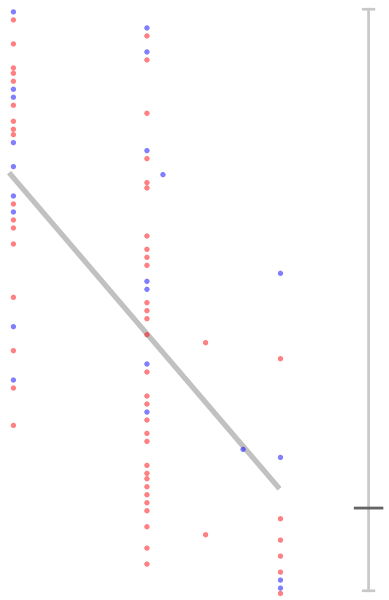

TT (26) TC (40) CC (11)

Corr: -0.612 **R2:** 0.375
Z-Score: -5.914 **P-Value:** 3.35E-9

Muscle

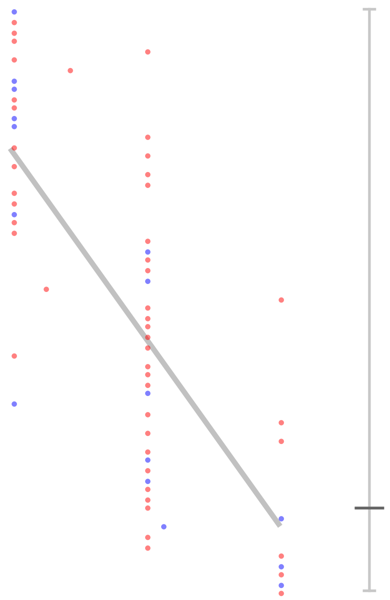

TT (22) TC (31) CC (9)

Corr: -0.73 **R2:** 0.533
Z-Score: -6.732 **P-Value:** 1.68E-11

Liver

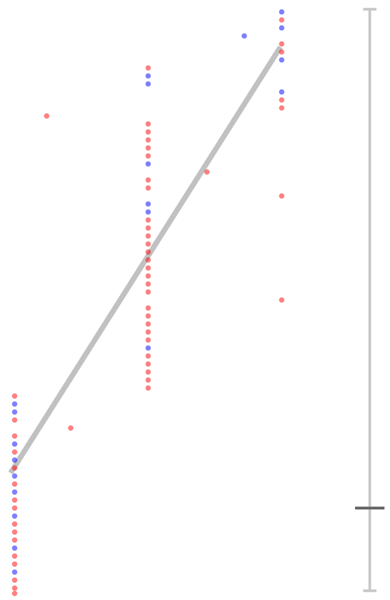

TT (27) TC (35) CC (12)

Corr: 0.85 **R2:** 0.723
Z-Score: 9.577 **P-Value:** 9.95E-22
