# Supplementary material for: Unraveling the Regulatory Mechanisms Underlying Tissue-Dependent Genetic Variation of Gene Expression
Source: PLoS Genet. 2012 Jan 19;8(1):e1002431. doi: 10.1371/journal.pgen.1002431 (PMC3261927; doi:10.1371/journal.pgen.1002431)
Supplement: Table S2 — The number of discordant cis-eQTL between blood and non-blood tissues. (DOC) [file pgen.1002431.s019.doc]

## Table S2. The number of discordant cis-eQTL between blood and non-blood tissues.

|  | Number of | | | |
| --- | --- | --- | --- | --- |
|  | Probe-SNP pairs | SNPs | Probes | Genes |
| Total *cis*-eQTL | 200,629 | 103,968 | 11,618 | 8,561 |
| Discordant *cis*-eQTLs (percentage) | 18,456 (9.2%) | 15,974 (15.4%) | 3,330 (28.7%) | 2,919 (34.1%) |
| Liver-blood discordant | 6,109 | 5,474 | 1,284 | 1,213 |
| SAT-blood discordant | 7,091 | 6,566 | 1,473 | 1,368 |
| VAT-blood discordant | 5,836 | 5,371 | 1,309 | 1,219 |
| Muscle-blood discordant | 4,671 | 4,359 | 1,022 | 1,013 |

SAT subcutaneous adipose tissue, VAT visceral adipose tissue
