# Supplementary material for: Unraveling the Regulatory Mechanisms Underlying Tissue-Dependent Genetic Variation of Gene Expression
Source: PLoS Genet. 2012 Jan 19;8(1):e1002431. doi: 10.1371/journal.pgen.1002431 (PMC3261927; doi:10.1371/journal.pgen.1002431)
Supplement: Table S3 — The Number of independent eSNPs per probe. (DOC) [file pgen.1002431.s020.doc]

**Table S3. The Number of independent eSNPs per probe.**

| **Tissue (Number of *eProbes*)** | **Single eSNPs** | **Multiple eSNPs** | | | |
| --- | --- | --- | --- | --- | --- |
|  | N=1 | N=2 | N=3 | N>3 | Total (N>1) |
| Blood (10,443) | 7,649 (73.2%) | 1,962 (18.8%) | 495 (4.7%) | 337 (3.2%) | 2,794 (26.8%) |
| Liver (713) | 704 (98.7%) | 9 (1.3%) | 0 | 0 | 9 (1.3%) |
| SAT (1,034) | 997 (96.4%) | 35 (3.4%) | 2 (0.2%) | 0 | 37 (3.6%) |
| VAT (808) | 773 (95.7%) | 30 (3.7%) | 4 (0.5%) | 1 (0.1%) | 35 (4.3%) |
| Muscle (352) | 345 (98.0%) | 6 (1.7%) | 0 | 1 (0.3%) | 7 (2.0%) |
