# Supplementary material for: Unraveling the Regulatory Mechanisms Underlying Tissue-Dependent Genetic Variation of Gene Expression
Source: PLoS Genet. 2012 Jan 19;8(1):e1002431. doi: 10.1371/journal.pgen.1002431 (PMC3261927; doi:10.1371/journal.pgen.1002431)
Supplement: Table S4 — Replication of tissue-alternative cis-eQTL of TMEM176A. (DOC) [file pgen.1002431.s021.doc]

**Table S4. *Replication of tissue-alternative cis-eQTL of TMEM176A.***

|  | *cis*-eQTL in liver rs714885 | *cis*-eQTL in blood rs6464104 |
| --- | --- | --- |
| *Discovery set* |  |  |
| Liver (*N* = 74) | *P* = 5.7 x 10^-6^ | *P* = 0.74 |
| Blood (*N* = 1,240) | *P* = 0.09 | *P* = 5.07 x 10^-132^ |
| *Independent validation set* | | |
| Liver (*N* = 427) | *P* = 1.8 x 10^-24^ | No significance reported |
| Blood (*N* = 229) | *P* = 0.3 | *P* = 1.3 x 10^-13^ |
