# Supplementary material for: Unraveling the Regulatory Mechanisms Underlying Tissue-Dependent Genetic Variation of Gene Expression
Source: PLoS Genet. 2012 Jan 19;8(1):e1002431. doi: 10.1371/journal.pgen.1002431 (PMC3261927; doi:10.1371/journal.pgen.1002431)
Supplement: Table S5 — Replication of cis-eQTL of DDT in blood and liver that show opposite allelic direction. (DOC) [file pgen.1002431.s022.doc]

## Table S5. Replication of cis-eQTL of DDT in blood and liver that show opposite allelic direction.

The allelic direction of eSNP rs5751777 that affected the expression of the *DDT* gene was replicated in independent blood and liver eQTL data. The rs5751777-T was coded as 1 and the rs5751777-C was coded as 2. The Spearman correlation coefficient between *DDT* expression and rs5751777 is listed below. The positive correlation coefficient means that allele C (the minor allele) was highly expressed. The negative correlation coefficient means that allele T (the major allele) was highly expressed.

|  | Correlation coefficient | *P* value |
| --- | --- | --- |
| *Discovery set* | | |
| Liver (*N* = 74) | 0.85 | 9.95 x 10^-22^ |
| Blood (*N* = 1,240) | -0.59 | 3.98 x 10^-119^ |
| *Independent validation set* | | |
| Liver (*N* = 427) | 0.66 | 2.86 x 10^-211^ |
| Blood (*N* = 229) | -0.61 | 4.37 x 10^-24^ |
