# Supplementary material for: Unraveling the Regulatory Mechanisms Underlying Tissue-Dependent Genetic Variation of Gene Expression
Source: PLoS Genet. 2012 Jan 19;8(1):e1002431. doi: 10.1371/journal.pgen.1002431 (PMC3261927; doi:10.1371/journal.pgen.1002431)
Supplement: Table S6 — Allelic effect of disease-associated SNPs on the expression of ORMLD3. (DOC) [file pgen.1002431.s023.doc]

## Table S6. Allelic effect of disease-associated SNPs on the expression of ORMLD3.

The expression variation of *ORMLD3* genes was associated with seven disease-associated SNPs. The identified over-expression haplotypes in lymphoblastoid cell lines (LCLs) were included and we assigned the allelic direction of their association in five primary tissues based on the over-expressed alleles in LCLs: positive *Z* values refer to the same allelic direction as in LCLs and negative *Z* values refer to the opposite allelic direction.

| SNP | Risk alleles (disease) | Genome position* | Haplo^#^ | *Cis*-effect on gene expression *Z-scores*s | | | | |
| --- | --- | --- | --- | --- | --- | --- | --- | --- |
|  |  |  |  | Blood (1,240) | SAT (83) | Liver (74) | VAT (77) | Muscle (62) |
| rs9303277 | T (primary biliary cirrhosis) | 35229995 | C | 13.26 | -3.63 | -0.79 | -2.46 | 2.37 |
| rs12936231 | C (asthma) | 35282646 | C | 13.48 | -3.81 | -0.92 | -2.45 | 2.29 |
| rs2872507 | A (Crohn's disease) | 35294289 | G | 14.21 | -3.6 | -1.2 | -2.48 | 1.49 |
| rs2305480 | A (Ulcerative colitis); G (Asthma) | 35315722 | G | 14.33 | -3.57 | -1.16 | -1.73 | 1.89 |
| rs2290400 | G (Type 1 diabetes) | 35319766 | T | 13.26 | -3.38 | -0.93 | -2.03 | 2.62 |
| rs7216389 | T (asthma) | 35323475 | T | 13.85 | -3.38 | -0.9 | -2.03 | -0.93 |
| rs6503525 | C (asthma) | 35348700 | C | 10.88 | -2.43 | -1.53 | -2.18 | 2.06 |

* The genome position in bp at chromosome 17, based on genome build 36.3

^#^The reported over-expressed haplotype in LCLs.
