# Supplementary material for: Unraveling the Regulatory Mechanisms Underlying Tissue-Dependent Genetic Variation of Gene Expression
Source: PLoS Genet. 2012 Jan 19;8(1):e1002431. doi: 10.1371/journal.pgen.1002431 (PMC3261927; doi:10.1371/journal.pgen.1002431)
Supplement: Table S11 — The number of the differentially expressed eProbes. (DOC) [file pgen.1002431.s028.doc]

Table S11: The number of the differentially expressed eProbes

| **Compared tissues (Number of *eProbes*, P value threshold)** | **Specific regulation** | **Alternative regulation** | **Different effect size** | **Opposite direction** | **Concordant regulation** |
| --- | --- | --- | --- | --- | --- |
| Liver vs. Blood (n=521, P<9.6x10^-5^) | 126/190 (66.3%) | 61/74 (82.4%) | 175/222 (78.9%) | 30/35 (85.7%) | 287/521 (55.1%) |
| SAT vs. Blood (n=708, P<7.1x10^-5^) | 157/232 (67.7%) | 81/99 (81.9%) | 272/347 (78.4%) | 18/27 (66.7%) | 457/708 (64.5%) |
| Vat vs. Blood (n=526, P<9.5x10^-5^) | 110/163 (67.5%) | 55/74 (74.3%) | 226/284 (79.6%) | 4/5 (80%) | 345/526 (65.6%) |
| Muscle vs. Blood (n=252, P<2.0x10^-4^) | 50/84 (59.5%) | 35/40 (87.5%) | 90/108 (83.3%) | 16/20 (80.0%) | 140/252 (55.6%) |
